# How host mobility formulations shape estimates of pathogen dispersal and epidemic risk in non endemic regions

**DOI:** 10.1371/journal.pcbi.1014646

**Published:** 2026-08-24

**Authors:** Charley Presigny, Piero Poletti, Stefano Merler, Manlio De Domenico

**Affiliations:** 1 Department of Physics & Astronomy ‘Galileo Galilei’, University of Padua, Padua, Italy; 2 Istituto Nazionale di Fisica Nucleare, Sez. Padova, Padua, Italy; 3 Center for Health Emergencies, Fondazione Bruno Kessler, Trento, Italy; 4 Padua Center for Network Medicine, University of Padua, Padua, Italy; University of Zaragoza: Universidad de Zaragoza, SPAIN

## Abstract

The global warming effects of climate change favor the stable residence of invasive vectors, such as the mosquito *Aedes albopictus*, in temperate areas, like the Mediterranean basin or north America. Thus, favorable weather conditions, together with human mobility, increase the likelihood of large-scale vector-borne disease epidemics in areas where they were historically not endemic. While mathematical modeling acknowledges the relevance of human mobility in the persistence or prevalence of vector-borne diseases in endemic areas, the combined role of host-mediated vector transport and long-range airline travel in the emergence of epidemics in non-endemic areas is poorly understood. In this context, the impact of specific modeling formulations of human mobility on the epidemic predictions remains elusive. To bridge this gap, we compare two alternative models - one based on the force of infection from different source locations and one based on the explicit physical diffusion of individuals - that incorporate intra- and inter-country human mobility (considering air traffic flows), vector dispersal, and human-mediated vector mobility (e.g., vectors in cars/trains), with applications to diseases such as chikungunya, dengue and Zika. Through extensive computational and analytical analysis, we assess how implicit assumptions and the inclusion of different mobility components influence model estimates of epidemic emergence risk, the timing of potential epidemics and the risk of epidemic spread. The comparison between the two frameworks is illustrated using synthetic networks and Italy as a realistic case study. Our findings show that different model formulations of human mobility yield divergent risk assessment, affecting predictions of large scale outbreaks and importation times driven by explained mechanistic difference. Despite these differences, both models show consistent qualitative patterns in the spatial risk and the likelihood of concurrent local outbreaks. Integrating multiple mobility components and carefully identifying assumptions that reflect observed mechanisms are essential for understanding epidemics in currently non-endemic areas.

## Author summary

Climate change is making temperate regions more suitable for mosquitoes, allowing them to survive longer and bite more frequently. This increases the chance that diseases such as dengue, chikungunya, and Zika can spread locally after infected travelers arrive. Human movement plays a key role in introducing and spreading these infections, sometimes across continents. However, epidemiological models represent mobility in different ways, and the consequences of these choices are not well understood. In this study, we propose two mathematical models integrating multiple mobility components; one in which the human movement is implicit, the other one where it is explicit. Using simulations and mathematical analysis, we examine how these approaches influence predictions of outbreak risk and timing. We show that despite showing similarities in the spatial spread of diseases, the two formulations yield to different predictions of vector-borne outbreaks due to mechanistic differences that we make explicit. Our results highlight the importance of carefully selecting how mobility is represented in models. Choosing an approach that matches the study context and goals can improve predictions and support more effective strategies to prevent or control mosquito-borne diseases in temperate regions that are increasingly at risk.

## Introduction

Mosquito-borne diseases such as chikungunya, Zika or dengue pose a growing threat to global health. Dengue alone places nearly half of the world’s population at risk, with an estimated 390 million infections occurring each year in tropical regions [1]. Climate change and human mobility are now extending this risk to new temperate areas [2,3]. Invasive species such as *Aedes albopictus* (the tiger mosquito) have spread widely through international trade, colonizing large areas of Europe (particularly the Mediterranean basin) and North America [4,5]. Imported cases from endemic regions, facilitated by long-distance human travel, have already triggered outbreaks in southern France [6], Italy [7,8], Spain [9] or Florida [10]. Understanding the interplay between mobility and transmission has therefore become a central challenge in epidemic modeling, supported by the growing availability of detailed mobility data over the past two decades [11–15]. For instance, mobile phone data [16] or GPS-based data [17] are now routinely used to parametrize epidemiological models and improve predictive accuracy. Metapopulation models provide a widely adopted framework for integrating human mobility and epidemic dynamics [13,18–21]. In these models, populations are structured as networks: nodes represent a spatial position, i.e., patches (e.g., neighborhoods, cities, regions etc..) where subpopulations interact homogeneously and links capture the mobility of subpopulation between nodes. This framework is highly scalable, from schematic models [22,23] to global data-driven simulations [24,25].

Different formulations exist for modeling mobility within this framework. One of the widely adopted formulation incorporates it into a force of infection representing the rate at which infected individuals become infected [25–29]. Here, each node exerts an effective force on the others that convert susceptible individual into infected ones according to their connectivity, without explicitly simulating individual movements [30]. A notable example is provided by vector-borne disease models incorporating residence times, where host mobility is represented by the fraction of time individuals from a given patch spend in other patches [31,32]. This formulation captures the resulting effective population in each patch and has been applied to derive epidemiological metrics such as risk indices and epidemic final sizes. [33–35] Another popular formulation is *diffusion* where populations effectively move from nodes to nodes according to a mobility pattern [19,36–38]. Although this approach introduces explicit movement, it can produce unrealistic dynamics for short-term mobility, as it effectively relocates infected individuals permanently across locations, amplifying the seeding of new outbreaks and altering local transmission dynamics. In contrast, the force-of-infection formulation represents mobility as an effective mixing process, rather than as the explicit displacement of individuals, thereby neglecting movement-driven mechanisms such as the recurrent and preferential visitation patterns of infected hosts to specific locations [39–41]. To address this, more recent models incorporate realistic mobility patterns [42–44] which have been shown to generate epidemic dynamics that are not equivalent to those produced by diffusion both in general settings [45] and in vector-borne disease transmission [46]. Additional studies explored heterogeneity in mobility, such as differences by socio-economic class [20] or the role of higher order memory effects [47].

However, most studies on vector-borne diseases focus on endemic regions, examining how human mobility or vector dispersal modulate disease persistence or prevalence [42,46,48]). In this work, we hypothesize that in non-endemic regions, the combined effects of human and vector mobility must be jointly modeled to accurately predict outbreak emergence. We therefore develop a metapopulation framework that integrates four aspects of mobility: (i) host mobility within the country, (ii) host-mediated vector transport, (iii) autonomous vector dispersal, and (iv) long-range airline travel.

**Fig 1:**
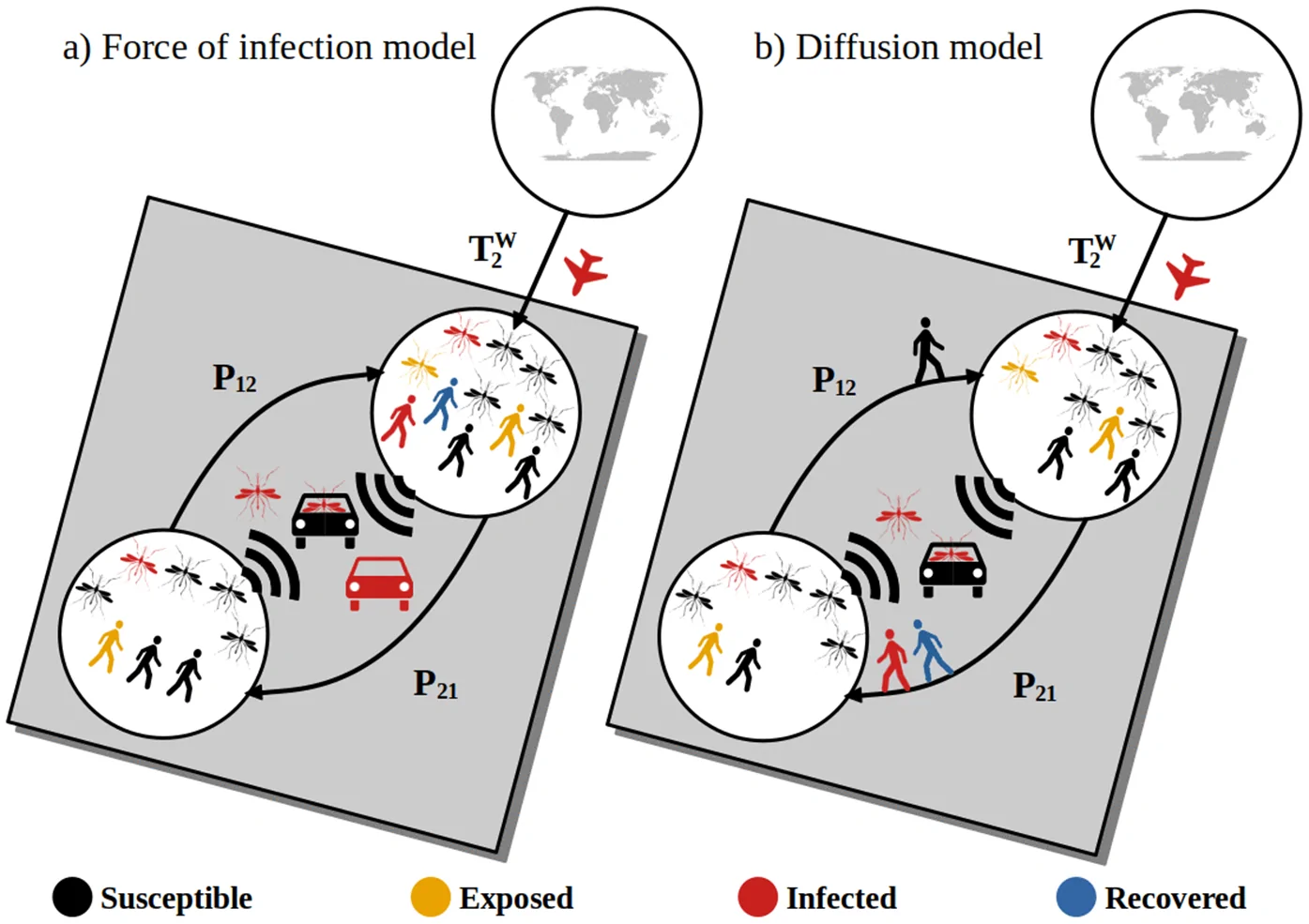
Illustration of the metapopulation models encompassing 4 mobility routes. The human and vector population locally interact in spatially distributed nodes according to the SEIR dynamics. Their colors indicate their epidemiological state. The human flow from node *i* to node *j* is represented with curved black arrows and encoded in the matrix element $P_{ij}$. The airline mobility is modeled as a infection reservoir in which infected hosts interact with susceptible vectors node *i* (straight black arrow) according to $T_{i}^{W}$, the ratio of host arrival in node *i* over all host arrival in the system. The birth-death process of hosts and vectors is not represented. **a)** In the force of infection model, the host mobility is encoded in the force of infection. There is no explicit movement between nodes. **b)** In the diffusion model, the host mobility is encoded as a diffusion process. Therefore, there is an explicit movement of hosts between nodes. The autonomous vector dispersal and the human-mediated host mobility are encoded in the force of infection for both models.

Because assumptions about host mobility can strongly influence epidemic outcomes [21,46], we propose two models to analyze two alternative formulations present in the literature: one in which host mobility is incorporated through the *force of infection* (FoI model), and another one in which it is incorporated as a *diffusion*. In both models, the other mobility processes are incorporated in the force of infection. Host-mediated vector transport accounts for mosquitoes being passively carried over long distances by vehicles, a mechanism that have been suggested to contribute substantially to mosquito dispersal [49,50]. Autonomous vector dispersal captures the short-range movement of mosquitoes from their birthplace, typically within a few hundred meters [51]. Airline travel introduces infected individuals arriving from endemic regions by plane, seeding local transmission (see Fig 1).

In what follows, we analyze the basic reproduction number (*R*_0_) and the attack rate for both host-mobility formulations to quantify their impact, using both synthetic and empirical networks. We consider two illustrative scenarios relevant to non-endemic areas: one in which the local *R*_0_ is below the epidemic threshold (i.e., *R*_0_ < 1) in all nodes, and one in which some nodes exceed the threshold, mimicking, respectively, winter conditions and peak mosquito activity during summer in temperate countries [52]. We further compare the formulations by assessing local importation risk using data-driven mobility estimates, defined as the probability of disease introduction from external sources. Differences in outcomes are illustrated using Italy as a realistic case study to support the comparison in real-world scenarios. The comparison is carried out across metrics capturing epidemic emergence, timing, and spatial spread.

## Materials and methods

### Metapopulation models

We consider a SEIR-SEI compartmental model including birth-death processes [53]. Subpopulation of hosts and vectors interact locally within nodes that are discrete and spatially distributed units. In each node *i*, hosts are divided into four compartments, i.e., susceptible ($S_{h}^{i}$), exposed ($E_{h}^{i}$), infected ($I_{h}^{i}$) or recovered ($R_{h}^{i}$) while vectors are divided into three compartments, i.e., susceptible ($S_{v}^{i}$), exposed ($E_{v}^{i}$), infected ($I_{v}^{i}$). $N_{h}^{i},N_{v}^{i}$ are the total host and vector population in node *i*, respectively. Only infected vectors can infect susceptible hosts and vice versa. We suppose that the total number of hosts and vectors remain constant ($\sum_{i}N_{h}^{i}(t)=N_{h},\sum_{i}N_{v}^{i}(t)=N_{v}$). Nodes are coupled through a mobility matrix 𝐏 where $P_{ij}$ represents the conditional probability that an individual departing from node *i* travels to node *j* such that $\sum_{j}{𝐏}_{ij}=1$. In addition, air travel passengers from endemic countries are modeled as an infection reservoir which follows a homogeneous SEIR-SEI dynamics. Infected air travel passengers are imported into nodes containing airports at a rate $N_{W}^{sys}T_{i}^{W}\frac{I_{h}^{W}}{N_{h}^{W}}$ [54] (see Fig 1). The list of parameters can be found in Table 1. Based on this metapopulation structure, the host mobility within the country is integrated according to two alternative formulations:

**Table 1:** Parameters of the models.

Epidemiological and biological		Mobility	
$T_{lh}$	host life expectancy	${𝐏}_{ij}$	conditional probability that an individual departing from node *i* travel to node *j*
$T_{lv}$	vector life expectancy	ϵ	time-scale separation parameter
$c_{hv}$	effective contact rate host to vector	*p*_0_	average number of vectors transported per moving host
$c_{vh}$	effective contact rate vector to host	*d*_0_	characteristic length of the autonomous vector dispersal
$\kappa_{i}$	vector carrying capacity in node *i*	$N_{W}^{sys}$	daily host arrival from the infection reservoir to the system
$T_{eit}$	extrinsic incubation time	$T_{i}^{W}$	proportion of hosts arrival from infection reservoir in node *i*
$T_{iit}$	intrinsic incubation time		
$T_{id}$	host infection duration		

#### Force of infection (FoI) model.

In the FoI model, the various mobility routes are encapsulated within a spatial infection kernel that defines the force of infection between nodes. Since the mechanistic differences between the two modeling formulations are primarily reflected in the infection rates of susceptible entities, we focus our description on the equations for susceptible hosts ($S_{h}^{i}$) and susceptible vectors ($S_{v}^{i}$); the complete SEIR-SEI systems are detailed in Text A in S1 Appendix:


$$\begin{array}{ccc} \frac{dS_{h}^{i}}{dt} & = & \frac{N_{h}^{i}-S_{h}^{i}}{T_{lh}}-\frac{S_{h}^{i}c_{vh}^{i}}{N_{h}^{i}}\sum_{j}I_{v}^{j}K_{h}^{ji} \end{array}$$ (1)

$$\begin{array}{ccc} \frac{dS_{v}^{i}}{dt} & = & \frac{\kappa^{i}-S_{v}^{i}}{T_{lv}}-\frac{S_{v}^{i}c_{hv}^{i}}{N_{h}^{i}}\sum_{j}\left(I_{h}^{j}+\delta_{ij}N_{W}^{sys}T_{i}^{W}\frac{I_{h}^{W}}{N_{h}^{W}}\right)K_{v}^{ji}, \end{array}$$ (2)

where $\delta_{ij}$ is the Kronecker delta. Let us denote $K_{h}^{ji}\propto e^{-\frac{d_{ji}}{d_{0}}}+\frac{p_{0}P_{ji}}{\epsilon }$, and $K_{v}^{ji}\propto \delta_{ij}+\frac{P_{ji}}{\epsilon }$, the infection kernel from node *j* to node *i* associated with the infection rate of susceptible hosts and susceptible vectors, respectively. The exponential term captures the autonomous vector dispersal in the host infection rate in node *i* [28]. It shows that an infected vector in *j* can move in node *i* and infect a susceptible host ($K_{h}^{ji}$).

$\frac{1}{\epsilon }$ is the host mobility rate, i.e., the fraction of host moving per unit of time while $\frac{P_{ij}}{\epsilon }$ is the transition rate between node *i* and node *j*. In this regard, ϵ provides the time scale separation between epidemic dynamics (i.e., reaction) and host mobility (i.e., diffusion). Host-mediated vector transport is modeled by $\frac{p_{0}P_{ji}}{\epsilon }$, where $p_{0}\ll 1$ represents the average number of vectors transported per moving host. The infection kernel is normalized so that $\sum_{j}K_{h}^{ij}=\sum_{j}K_{v}^{ij}=1$. Finally, airline travel contributes an additional term, representing the fraction of infected individuals imported daily from the infection reservoir into airport-associated nodes $N_{W}^{sys}T_{i}^{W}\frac{I_{h}^{W}}{N_{h}^{W}}$, without altering the local number of hosts.

#### Diffusion model.

In the diffusion model, host movement is explicitly represented as a reaction–diffusion process. Note that the diffusion term is present for all host compartments (see Text A in S1 Appendix for the complete SEIR-SEI model):


$$\begin{array}{ccc} \frac{dS_{h}^{i}}{dt} & = & \frac{N_{h}^{i}-S_{h}^{i}}{T_{lh}}-\frac{S_{h}^{i}c_{vh}^{i}}{N_{h}^{i}}\sum_{j}I_{v}^{j}K_{h}^{ji}+\frac{1}{\epsilon }\left[\sum_{j=1}^{M}P_{ji}S_{j}-P_{ij}S_{i}\right] \end{array}$$ (3)

$$\begin{array}{ccc} \frac{dS_{v}^{i}}{dt} & = & \frac{\kappa^{i}-S_{v}^{i}}{T_{lv}}-\frac{S_{v}^{i}c_{hv}^{i}}{N_{h}^{i}}\sum_{j}\left(I_{h}^{j}+\delta_{ij}N_{W}^{sys}T_{i}^{W}\frac{I_{h}^{W}}{N_{h}^{W}}\right)K_{v}^{ji}, \end{array}$$ (4)

where $\delta_{ij}$ is the Kronecker delta. Hosts move between nodes at the transition rates proportional to $\frac{P}{\epsilon }$, where the scaling parameter ϵ controls the relative timescale of mobility versus epidemic dynamics [18,55]. When ϵ>1, the epidemics dynamics is faster than the mobility; when ϵ<1, mobility dominates. We denote $K_{h}^{ji}\propto e^{-\frac{d_{ji}}{d_{0}}}+\frac{p_{0}P_{ji}}{\epsilon }$, and $K_{v}^{ji}=\delta_{ij}$, the infection kernel from node *j* to node *i* associated with the infection rate of susceptible hosts and susceptible vectors, respectively. Since the host mobility is explicitly accounted by the diffusion term, $K_{v}^{ji}$ is the unit matrix, i.e., susceptible vectors are only infected locally by infected hosts. The host-mediated vector transport, the autonomous vector dispersal and the infection reservoir are modeled identically as in the FoI model.

### Construction of the Italian mobility networks

We construct two real-world networks for Italy: one at the regional level with 20 regions (NUTS2) and one at the provincial level with 107 provinces (NUTS3).

#### Population data and distance.

Population data were obtained from the Comprehensive World Cities Database (Simplemaps, version 1.77, updated in 2024). City-level data were aggregated to the appropriate administrative level (NUTS2 or NUTS3). This database reports a total Italian population of around 55.6 million. The geographical position of each node was defined as the average coordinates of the cities it contains. Distances between nodes were computed using geodesic distances, accounting for the curvature of the Earth.

#### Mobility data.

Mobility matrices were derived from Cuebiq data (publicly available [17]). We focused on mobility flows on 18th January 2020 before any COVID-19-related lockdowns. The conditional probability that an individual departing from node *i* travels to node *j* was computed as $P_{ij}=\frac{c_{ij}*p_{i}}{u_{i}}$, $c_{ij}$ is the number of time a user from node *i* move to node *j*, $p_{i}$ is the population of node *i* and $u_{i}$ the sample size of users recorded in node *i*. Finally, we impose $\sum_{j}P_{ij}=1$. Airline traffic data were obtained from the Italian civil aviation authority (ENAC) [56] to determine the size and position of airports. We computed the total number of arrivals from international destinations per airports and integrate at the level of NUTS2 and NUTS3. We then normalized by the total number of arrivals (more than 63 million passengers in 2019) to estimate the proportion of each unit $T_{i}^{W}$ in capturing the total Italian airline traffic. Then we estimated the proportion of arrivals and departures from and to countries currently at risk of vector-borne diseases over the total international traffic (approximatively 6%). Countries at risk are defined as countries where the risk of dengue is frequent or sporadic as assessed by the CDC [57]. Assuming that the arrivals are similar to the departures it allowed us to estimate the average daily arrivals in Italy to $N_{W}=11003$ daily passengers from countries at risk.

### Estimating the time-scale separation parameter

The mobility parameter ϵ is estimated from time-resolved mobility matrices provided by the Meta Data 4 Good Program [58]. These matrices are used exclusively for the estimation of ϵ; all other analyses in the Results section are based on the pre-lockdown mobility matrix derived from Cuebiq data, as described in the previous section. The Meta Data 4 Good Program is a collaborative initiative that provides aggregated GPS-based datasets of Facebook users that opted-in for the collection of their data. The Meta Data 4 Good Program provides estimates of the mobility flow between local administrative areas at the NUTS3 level for European countries [59]. These flows are built by aggregating weekly movements of Facebook users that opted-in to the location-based services. The flows are reported by sequence of 8 hours (0–8, 8–16, and 16–24) along with the userbase in each unit. The precise methodology is detailed in [59]. For Italy, we constructed weekly mobility matrices covering 12 weeks between 16th June 2020 and 7th September 2020 corresponding to the absence of lockdown and the seasonal presence of mosquitos. We averaged the 8 hours userbase for each week to obtain the weekly active users $u_{i}$ in each node. The database provides the weekly mean co-locations $f_{ij}$, i.e., the average fraction of time in which pairs of users from province *i* and *j* were observed together. The conditional probability that an individual departing from node *i* travels to node *j* is upscaled by computing $P_{ij}=\frac{f_{ij}*p_{i}}{u_{i}}$, where $p_{i}$ is the population of node *i*. Finally, we impose $\sum_{j}P_{ij}=1$. Here, we assume that the population of Facebook users in node *i* active a time *t* is a good proxy of the global population of node *i*, i.e., $N_{h}^{i}(t)=u_{i}(t)$.

For any epidemiological state, the evolution of the number of host $N_{h}^{i}(t)=S_{h}^{i}(t)+E_{h}^{i}(t)+I_{h}^{i}(t)+R_{h}^{i}(t)$ in node *i* read:


$$\begin{array}{c} \frac{dN_{h}^{i}(t)}{dt}=\frac{1}{\epsilon }\left[\sum_{j=1}^{M}P_{ji}N_{h}^{j}(t)-N_{h}^{i}(t)\right] \end{array}$$ (5)

We assume that within the time span Δt=7 days, the evolution of population $\Delta N_{h}^{i}$ is small. This choice balances the need to reduce short-term fluctuations (e.g., weekends) while retaining enough samples to resolve dynamics. In full generality, ϵ can be node dependent $\epsilon_{i}$. Rewriting the above equation under these assumptions yields:


$$\begin{array}{c} \epsilon_{i}=\frac{\left(\sum_{j=1}^{M}P_{ji}N_{h}^{j}(t)-N_{h}^{i}(t)\right)\Delta t}{N_{h}^{i}(t+\Delta t)-N_{h}^{i}(t)}, \end{array}$$ (6)

where $N_{h}^{i}(t)$ and $N_{h}^{i}(t+\Delta t)$ is the population of active Facebook user in node *i* in the week *t* and in week *t* + 1. Since the total population of active users changes from one week to ano*t*her, *t*he population are normalized ($N_{h}^{i}(t+\Delta t)=N_{h}^{i}(t+\Delta t)\frac{\sum_{i}N_{h}^{i}(t)}{\sum_{i}N_{h}^{i}(t+\Delta t)}$) to account only for the change in the population due to mobility.

### Estimating the local importation risk for chikungunya

To estimate the local importation risk, we perform stochastic simulations of the FoI and diffusion model using the Gillespie algorithm on the Italian provinces (NUTS3). In particular, we use the τ-leaping approximation scheme with τ=0.05 days to have a reasonable computational time while ensuring that the average of the stochastic trajectories are consistent with the deterministic dynamics [60]. We define the local importation risk as the ratio of simulations where a province (NUTS3) is infected over the total number of simulations, i.e., the probability that a province is infected. Probabilities are estimated over 1000 simulations of the stochastic version of the FoI and diffusion model, for each scenario. Serological evidence from a historical chikungunya outbreak in Italy shows an estimated 45% detection rate [61]. We therefore assume that infection in a province can be detected only when at least two infections occur, since single cases are likely to remain unnoticed. The ISS (the Italian institute of health) reports that Italy experienced 41 imported chikungunya cases from January to September 2025 [62] corresponding to approximatively 1 per week nationwide. Dividing per the number of provinces, we fix the rate of injection from the airline mobility to 0.01 people per week, i.e., $N_{W}^{sys}T_{i}^{W}\frac{I_{h}^{W}}{N_{h}^{W}}=0.01$ ${week}^{-1}$. For assessing the importation risk, we parameterize the models for chikungunya (CHIKV) carried by *Aedes albopictus* (see Table A in S1 Appendix).

## Results

**Fig 2:**
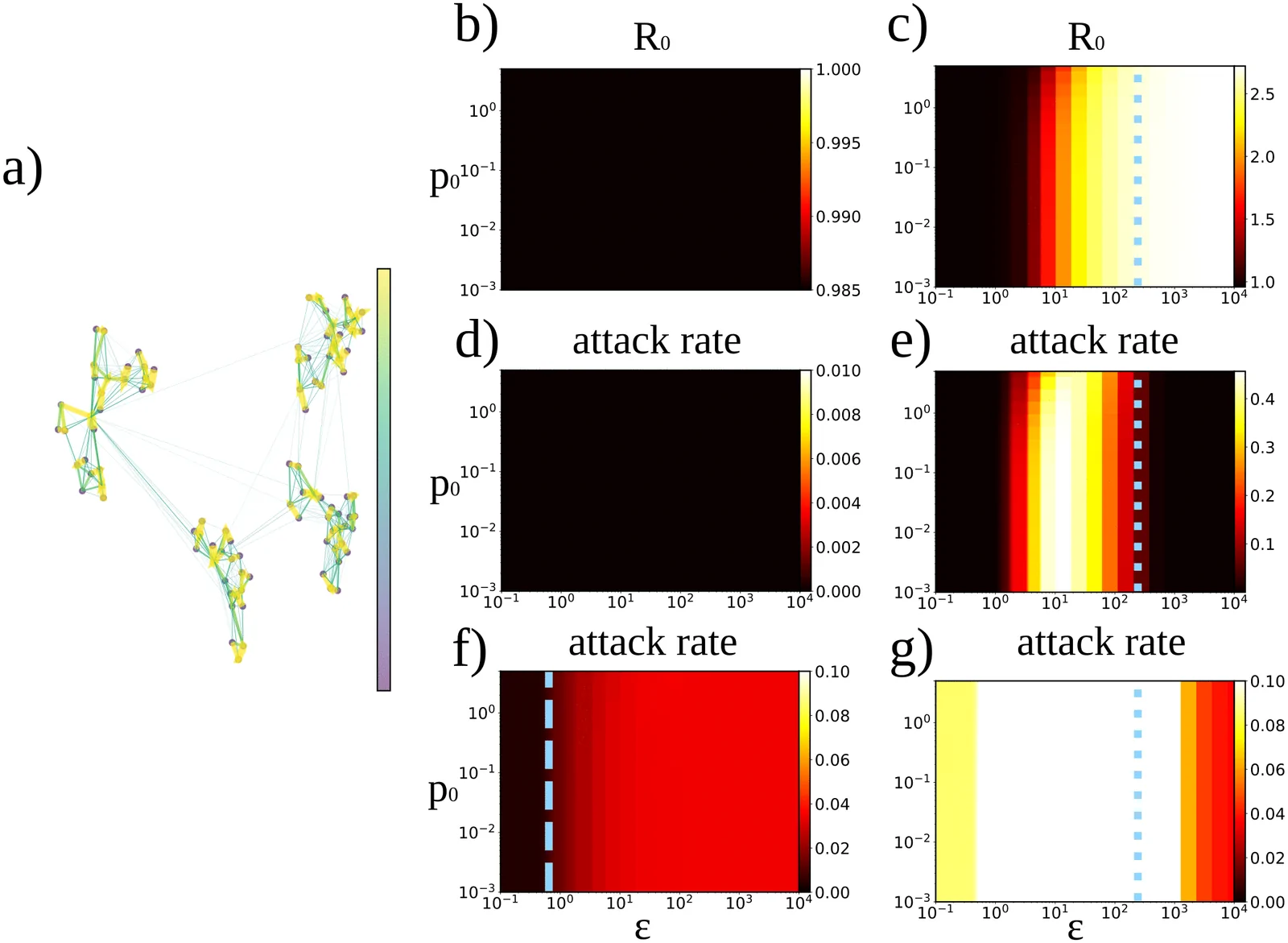
Phase diagrams in the ($\epsilon ,p_{0}$) space for synthetic networks. a) Illustration of a 100 nodes synthetic network whose spatial distribution is generated by the Soneira-Peebles model. Link from node *i* to *j* are shown for $P_{ij}>0.01$. Phase diagram of the basic reproduction number *R*_0_ for the force of infection model **b)** and the diffusion model **c)**. Attack rate without infection reservoir for the force of infection model **d)** and the diffusion model **e)**. Attack rate with infection reservoir for the force of infection model **f)** and the diffusion model **g)**. ϵ is the variable associated with human mobility and *p*_0_ is the one associated with human-mediated vector mobility. The blue dashed line is the estimated threshold between slow and fast mobility interactions for the FoI model. The blue dotted line corresponds to $\epsilon =\frac{t_{max}}{3}=243$, the limit after which the host mobility diffusion does not reach equilibrium within realistic time within 2 years ($t_{max}=730$). Individual *R*_0_ = 0.98 for each node.The basic reproduction number for the infection reservoir is set to $R_{0}^{W}=2$. Results are averaged over 100 synthetic networks.

**Fig 3:**
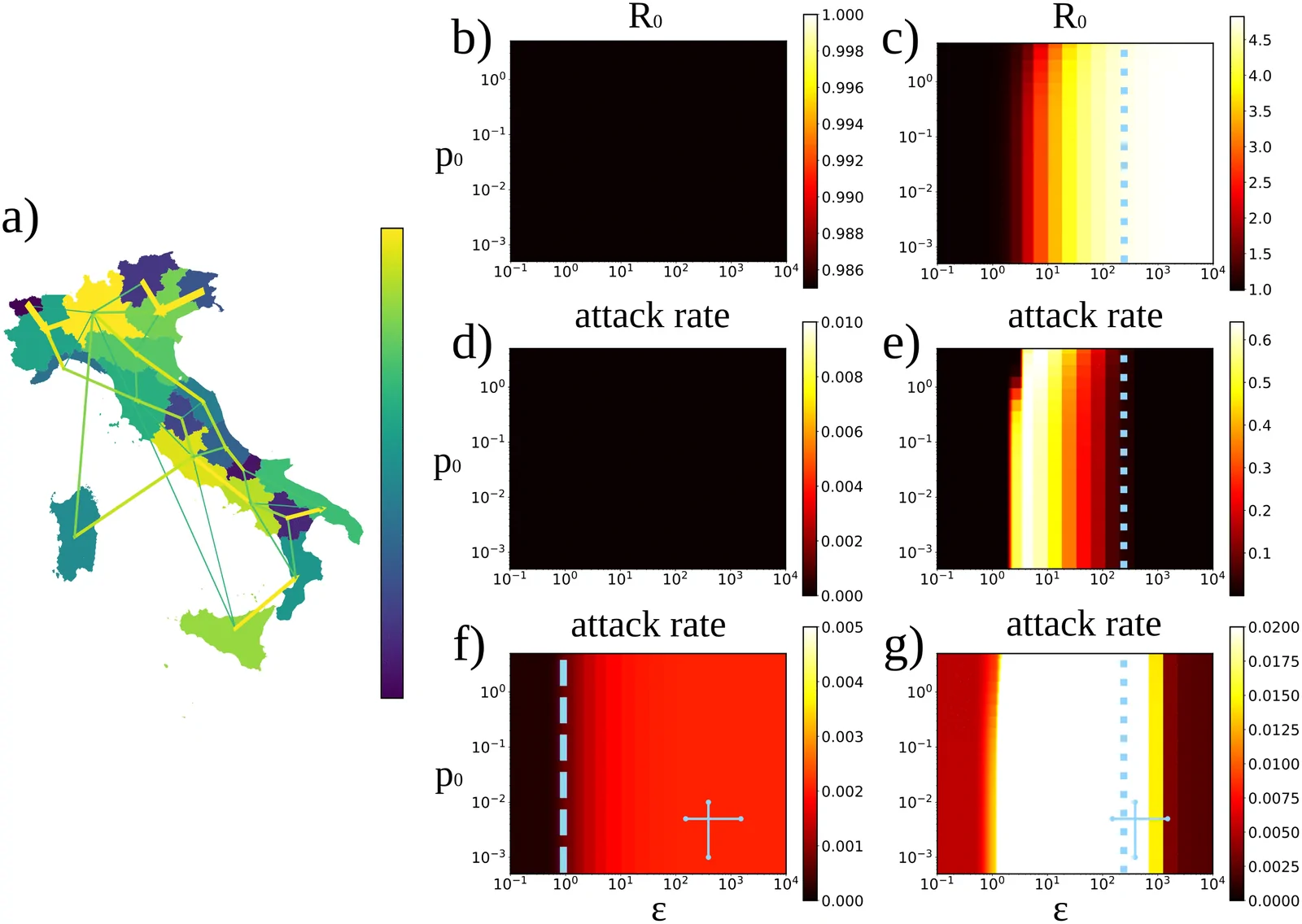
Phase diagrams in the ($\epsilon ,p_{0}$) space for Italian regions (NUTS2). a) Illustration of the Italian regions and the mobility flow between them. Colors of regions and arrows indicate their relative size and intensity, respectively. Link from node *i* to *j* are shown for $P_{ij}>0.1$. *R*_0_ for the force of infection model **b)** and the diffusion model **c)**. Attack rate without infection reservoir for the force of infection model **d)** and for the diffusion model **e)**. Attack rate with infection reservoir for the force of infection model **f)** and for the diffusion model **g)**. ϵ is the variable associated with human mobility and *p*_0_ is the one associated with human-mediated vector mobility. The blue dashed line is the estimated threshold between slow and fast mobility. Horizontal and vertical plain lines are the estimation of the uncertainty range for empirical ϵ and *p*_0_.The blue dotted line corresponds to $\epsilon =\frac{t_{max}}{3}=243$, the limit after which the host mobility diffusion does not reach equilibrium. *R*_0_ = 0.98 for each node. $R_{0}^{W}=2$ for the infection reservoir. Basemap administrative boundaries are realized by ISTAT data, via the Openpolis geojson-italy repository under the Creative Commons Attribution 4.0 (CC BY 4.0) license.

**Fig 4:**
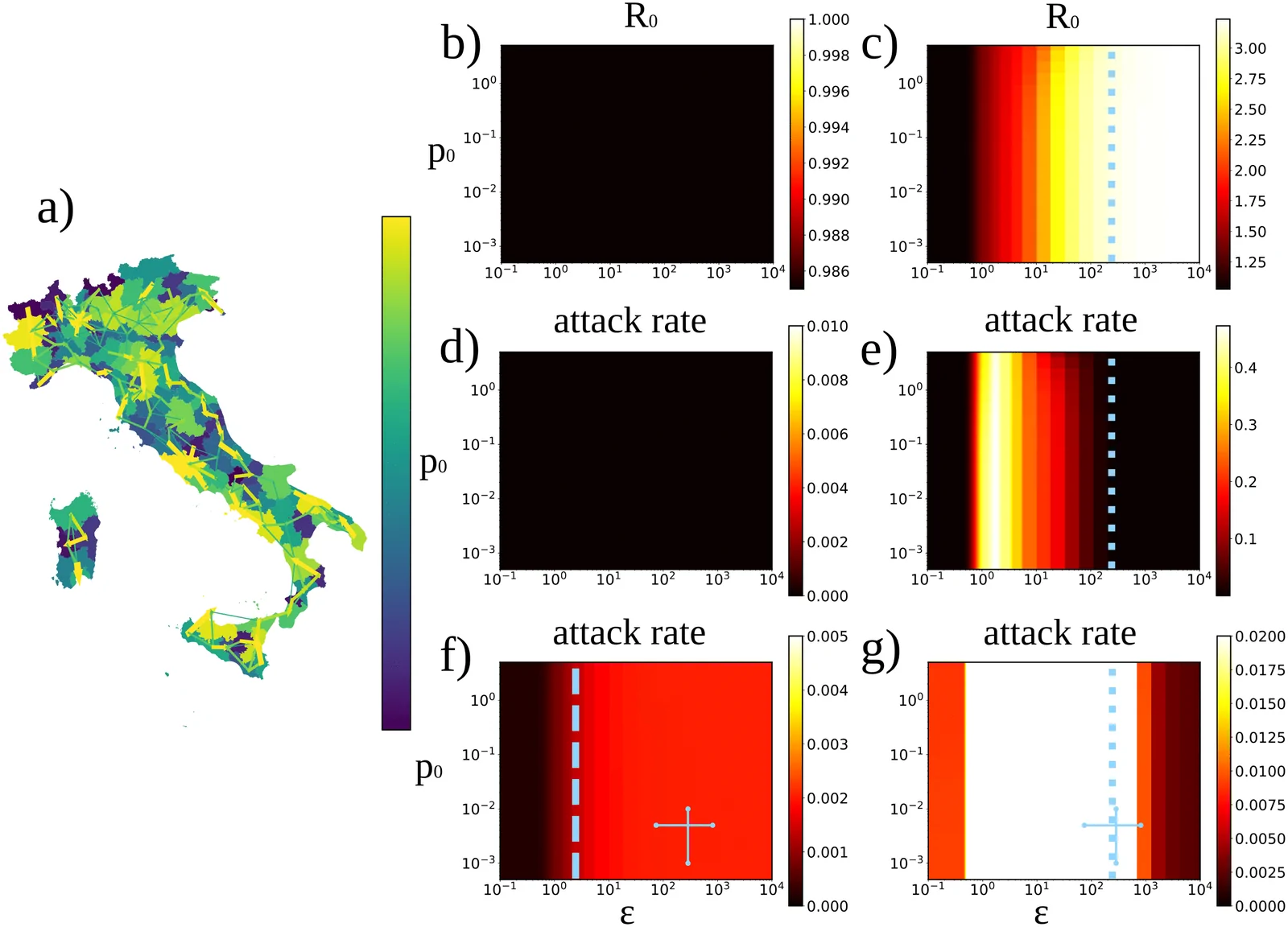
Phase diagrams in the ($\epsilon ,p_{0}$) space for Italian provinces (NUTS3). a) Illustration of the Italian provinces and the mobility flow between them. Colors of regions and arrows indicate their relative size and intensity, respectively. Link from node *i* to *j* are shown for $P_{ij}>0.1$. *R*_0_ for the force of infection model **b)** and the diffusion model **c)**. Attack rate without infection reservoir for the force of infection model **d)** and for the diffusion model **e)**. Attack rate with infection reservoir for the force of infection model **f)** and for the diffusion model **g)**. ϵ is the variable associated with human mobility and *p*_0_ is the one associated with human-mediated vector mobility. Horizontal and vertical plain lines are the estimation of the uncertainty range for empirical ϵ and *p*_0_. The blue dashed line is the estimated threshold between slow and fast mobility. The blue dotted line corresponds to $\epsilon =\frac{t_{max}}{3}=243$, the limit after which the host mobility diffusion does not reach equilibrium. *R*_0_ = 0.98 for each node. $R_{0}^{W}=2$ for the infection reservoir. Basemap administrative boundaries are realized by ISTAT data, via the Openpolis geojson-italy repository under the Creative Commons Attribution 4.0 (CC BY 4.0) license.

The primary objective of this study is to quantify and compare the influence of mobility on vector-borne epidemic dynamics under two different formulations. Specifically, we investigate how mobility features shape the onset of epidemics in systems that operate below the epidemic threshold mimicking unfavorable conditions for vector proliferation typically observed in temperate areas during winter months. To this end, we analyze an ensemble of synthetic networks and Italy at two different scales, NUTS2 (regions) and NUTS3 (provinces). Synthetic networks are generated from the Soneira-Peebles model that provides a realistic node spatial distribution [63]. Node populations are set following a Zipf law and the mobility coupling is derived from the radiation model (see Fig 2A and Text B in S1 Appendix for details). For the empirical networks, population of Italian administrative units were aggregated from city-level data and mobility flows were estimated using GPS sensor data (see Methods and see Figs 3A and 4A). Since the minimal distance between nodes in the all systems is of the order of tens of kilometers, we disregard the influence of the vector autonomous dispersal in the following analysis (*d*_0_ = 150m). Unless otherwise stated, we parameterize the models for dengue (DENV) carried by *Aedes albopictus* using values from the literature (see Table A in S1 Appendix). Parameters are kept constant across nodes. We set the effective contact rates host-to-vector $c_{hv}=0.11$ and vector-to-host $c_{vh}=0.12$ such that the basic reproduction number of each node *i* in the system remains slightly below the epidemic threshold, i.e., $R_{0}\approx 0.98<1$. For the infection reservoir we set $R_{0}^{W}=2$ ($c_{vh}^{W}=0.25$, $c_{hv}^{W}=0.22$). This approach is consistent with literature as effective contact rates are often treated as free parameters due to its difficulty to obtain precise empirical estimates [25]. In the following, we will refer to this parametrization as our *case study*.

### Analysis of the basic reproduction number *R*_0_ and the attack rate

To asses the impact of our mobility parameters on the vector-borne disease dynamics, we compute the basic reproduction number *R*_0_ using the next-generation matrix method [64].

Since *R*_0_ is defined at equilibrium, it is not possible to consider the contribution of airline mobility as the number of individuals from the infection reservoir entering the system at equilibrium is zero. The airline mobility contribution is visible only out-of-equilibrium. The parameter ϵ represents the time-scale separation between mobility and epidemic dynamics. When ϵ≪1, host mobility is much faster than the infection process (fast mobility regime). Conversely, when ϵ≫1, host mobility is slow relative to disease spread (slow mobility regime), and local epidemiological dynamics dominate over spatial displacement. For the sake of readability, we first present the analysis of the FoI model, then the diffusion model before comparing them.

#### Force of infection model.

We can show that the basic reproduction number associated with the FoI model $R_{0}^{F}$ read (see Text C in S1 Appendix for derivation):


$$\begin{array}{c} R_{0}^{F}=max\left(\sqrt{\rho (𝒜)},\sqrt{\rho (ℬ)}\right), \end{array}$$ (7)

with ρ(𝒜),ρ(ℬ), the spectral radius of the matrix 𝒜 and matrix ℬ respectively. The latter are defined as:


$$\begin{array}{ccc} 𝒜 & = & {C^{\prime }}_{vh}K_{h}^{⊤}\frac{T_{lv}^{2}}{T_{eit}+T_{lv}}{𝒞}_{hv}K_{v}^{⊤}⊙\kappa^{⊤}\left(\frac{T_{id}T_{lh}^{2}}{\left(T_{id}+T_{lh})(T_{iit}+T_{lh}\right)}\right) \end{array}$$ (8)

$$\begin{array}{ccc} ℬ & = & {𝒞}_{hv}K_{v}^{⊤}⊙\kappa^{⊤}\left(\frac{T_{id}T_{lh}^{2}}{\left(T_{id}+T_{lh})(T_{iit}+T_{lh}\right)}\right){C^{\prime }}_{vh}K_{h}^{⊤}\frac{T_{lv}^{2}}{T_{eit}+T_{lv}} \end{array}$$ (9)

with ${C^{\prime }}_{vh}=diag(c_{vh}^{1},...,c_{vh}^{j},...)$, ${𝒞}_{hv}=diag(\frac{c_{vh}^{1}}{N_{h}^{1}},...,\frac{c_{vh}^{j}}{N_{h}^{j}},...)$, $\kappa =(\kappa^{1},...,\kappa^{j},...)$ and ⊙ the element-wise multiplication. Next, wecompute the phase diagram for $R_{0}^{F}$ in the ($\epsilon ,p_{0}$) space for synthetic networks and Italian networks (see Figs 2B, 3B and 4B). We divide the phase diagram into three regimes.

**Case** ϵ≫1 **and** $\epsilon \gg p_{0}$. It corresponds to the case of no mobility interactions. Using perturbation analysis, we derive $R_{0}^{F}$ at the first order (see Text D in S1 Appendix for derivation):


$$\begin{array}{c} R_{0}^{F,1}=max_{i}\left(\sqrt{\frac{T_{id}T_{lh}^{2}T_{lv}^{2}}{\left(T_{id}+T_{lh})(T_{iit}+T_{lh})(T_{eit}+T_{lv}\right)}\left(\frac{\frac{c_{vh}^{i}c_{hv}^{i}\kappa^{i}}{N_{h}^{i}}+(c_{vh}^{i}\sum_{l\neq i}(P^{⊤}{)}_{il}\frac{c_{hv}^{l}\kappa^{l}}{N_{h}^{l}}(P^{⊤}{)}_{li})\frac{p_{0}}{\epsilon^{2}}}{\left(1+\frac{1}{\epsilon })(1+\frac{p_{0}}{\epsilon }\right)}\right)}\right) \end{array}$$ (10)

By setting ϵ→∞, we recover the classical SEIR case $R_{0}^{F}=R_{0}^{SEIR}\approx 0.98<1$ [65]. Numerical simulations confirm the absence of outbreak across synthetic and Italian networks in our case study (see Figs 2B, 3B and 4B).

**Case** ϵ≪1 **and** $\epsilon \gg p_{0}$. Here hosts move extremely fast between nodes but vectors remain localized. For our case study, the spectral radius of 𝒜 and ℬ are both proportional to the one of the mobility matrix $P^{⊤}$ (see Text D in S1 Appendix). Since $\rho (P^{⊤})=1$, $R_{0}^{F}$ remains unchanged compared to the no-mobility case, we observe an absence of outbreak. This analytical prediction matches our numerical observations (see Figs 2B, 3B and 4B).

**Case** ϵ≪1 **and** $\epsilon \ll p_{0}$. In this regime, both vectors and hosts interact across all nodes according to the mobility matrix. In our case study, we show that the spectral radius of 𝒜 and ℬ is proportional to the one of $(P^{⊤}{)}^{2}$. Since $\rho (P^{⊤}{)}^{2}=1$, *R*_0_ < 1. Again, this leads to the absence of outbreak which is again consistent with our numerical observations (see Figs 2B, 3B and 4B). By deriving a lower bound for $R_{0}^{F}$ in this regime, we estimate the critical point $\epsilon^{*}$ separating slow and fast mobility (see Text D in S1 Appendix):


$$\begin{array}{c} \epsilon^{*}=\frac{(P^{{⊤}^{2}}{)}_{max,max}}{1-(P^{{⊤}^{2}}{)}_{max,max}} \end{array}$$ (11)

with $(P^{{⊤}^{2}}{)}_{max,max}$ the maximum of the diagonal of $(P^{⊤}{)}^{2}$. Here $(P^{⊤}{)}_{ij}^{2}$ denotes the conditional probability for an individual departing from node *i* to travel to node *j* after traveling to any node *k* and its diagonal elements capture the contribution of return paths to the same node. In particular, its maximum diagonal element reflects the largest weights of such return paths. Higher values of this quantity widen the slow-mobility region of the phase space allowing importation-driven outbreaks to occur under a broader range of conditions. Our numerical simulations are in good agreement with our analytical estimates (see dashed lines in Figs 2F, 3F and 4F).

Across synthetic and Italian networks, the attack rate matches the prediction of the basic reproduction number (see Figs 2D, 3D and 4D). The standard deviation of the attack rate for the synthetic networks ensemble is negligible since we observe that an absence of dynamics happens in this case (see Fig A in S1 Appendix). Incorporating airline mobility reveals 2 zones in the mobility phase space (see Figs 2F, 3F and 4F). For fast internal host mobility (ϵ→0), we observe an absence of outbreaks whereas for slow internal mobility, the injection of infected air travelers sustains outbreaks. Mechanistically, in the FoI model, fast internal mobility prevents outbreaks in visited nodes because the time spent by hosts in each node becomes too short for local vectors to effectively acquire infection from transient infected travelers (see Eq. 2).

#### Diffusion model.

The basic reproduction number $R_{0}^{D}$ associated with the diffusion model read (see Text C in S1 Appendix for derivation):


$$\begin{array}{c} R_{0}^{D}=max\left(\sqrt{\rho (𝒞)},\sqrt{\rho (𝒟)}\right), \end{array}$$ (12)

with ρ(𝒞),ρ(𝒟), the spectral radius of the matrix 𝒞 and matrix 𝒟, respectively. The latter are defined as:


$$\begin{array}{ccc} 𝒞 & = & {C^{\prime }}_{vh}{𝒦}_{h}^{⊤}\frac{T_{lv}^{2}}{T_{eit}+T_{lv}}({𝒦}_{v}{𝒞}_{hv}⊙\kappa^{⊤})\frac{1}{T_{iit}}{\left(\frac{T_{id}+T_{lh}}{T_{id}T_{lh}}+\frac{1}{\epsilon }{ℒ}^{⊤}\right)}^{-1}{\left(\frac{T_{iit}+T_{lh}}{T_{iit}T_{lh}}+\frac{1}{\epsilon }{ℒ}^{⊤}\right)}^{-1} \end{array}$$ (13)

$$\begin{array}{ccc} 𝒟 & = & ({𝒦}_{v}{𝒞}_{hv}⊙\kappa^{⊤})\frac{1}{T_{iit}}{\left(\frac{T_{id}+T_{lh}}{T_{id}T_{lh}}+\frac{1}{\epsilon }{ℒ}^{⊤}\right)}^{-1}{\left(\frac{T_{iit}+T_{lh}}{T_{iit}T_{lh}}+\frac{1}{\epsilon }{ℒ}^{⊤}\right)}^{-1}{C^{\prime }}_{vh}{𝒦}_{h}^{⊤}\frac{T_{lv}^{2}}{T_{eit}+T_{lv}}, \end{array}$$ (14)

with $L^{⊤}$, the transpose of the Laplacian matrix of the mobility network. Next, we compute the phase diagram for $R_{0}^{D}$ in the ($\epsilon ,p_{0}$) space for synthetic networks and Italian networks (see Figs 2C, 3C and 4C). We remark that ${𝒦}_{v}^{⊤}={𝒦}_{v}=𝐈$ in the diffusion model.

**Case** ϵ≫1 **and** $\epsilon \gg p_{0}$. In this regime, diffusion is infinitely slow ($\frac{L^{⊤}}{\epsilon }\to 0$), so the system effectively behaves as if nodes do not interact. Once equilibrium is reached, hosts remains localized and the basic reproduction number of each node can be approximated by the classical SEIR formula for the basic reproduction number $R_{0}^{SEIR}$ with updated equilibrium host population *i*, $N_{eq}^{i}$. In our case study, $R_{0}^{SEIR}$ read:


$$\begin{array}{c} R_{0}^{SEIR}=\sqrt{\frac{T_{id}T_{lh}^{2}T_{lv}^{2}c_{vh}c_{hv}}{\left(T_{id}+T_{lh})(T_{iit}+T_{lh})(T_{eit}+T_{lv}\right)}}{\text{max}}_{i}(\sqrt{\frac{\kappa_{i}}{N_{eq}^{i}}}) \end{array}$$ (15)

The number of vectors in node *i* $\kappa_{i}=MN_{h}^{i}$ is fixed in our case study. As a result, the vector-to-host ratio increases $\frac{MN_{h}^{i}}{N_{eq}^{i}}>M$ in nodes where the equilibrium host population decreases, which can drive the system above the epidemic threshold ($R_{0}^{D}>1$). Comparing theoretical predictions with numerical simulations confirm the epidemics onset: $R_{0}^{SEIR}$ and the observed basic reproduction number $R_{num}^{D}$ are in good agreement for a synthetic network of the considered ensemble (2.74,2.74), the Italian regions (4.83,4.83) and the Italian provinces (3.10,3.24) (see Figs 2C, 3C and 4C).

**Case** ϵ≪1 **and** $\epsilon \gg p_{0}$. In the cases where ϵ≪1, the inverse matrix of Eqs.13, 14 is ill-defined. Nonetheless, by expressing the matrix 𝐕 in terms of its (left-)right eigenvectors, it is possible to show that 𝒞 and 𝒟 reduce to (see Text E in S1 Appendix for derivation):


$$\begin{array}{cc} 𝒞 & \sim {𝒞}_{vh}^{\prime }({𝒞}_{hv}⊙\kappa^{⊤}){𝐒}^{2} \end{array}$$ (16)

$$\begin{array}{cc} 𝒟 & \sim ({𝒞}_{hv}⊙\kappa^{⊤})S^{2}{𝒞}_{vh}^{\prime }, \end{array}$$ (17)

where 𝐒 is $N_{patch}\times N_{patch}$ matrix where each column is the same and equal to $\frac{1}{2m}(s_{1}...s_{n}{)}^{⊤}$, $s_{i}$ being the strength of node *i* and *m* the total strength of the mobility network.

In our case study, ρ(𝒞) and ρ(𝒟) reduce to the no mobility case. This result matches the numerical simulations since we observe no outbreak for ϵ≪1 and $\epsilon \gg p_{0}$ in our numerical simulations (see Figs 2C, 3C and 4C).

**Case** ϵ≪1 **and** $\epsilon \ll p_{0}$. Using the exact same canvas as before, we show that 𝒞 and 𝒟 reduce again to the no mobility case in our case study (see Text E in S1 Appendix for derivation). This is again consistent with our numerical simulations (see Figs 2C, 3C and 4C).

For ϵ≪1 (fast mobility), the attack rate matches the prediction of the reproduction number $R_{0}^{D}$ consistent with the absence of epidemics. However, for ϵ≫1 (slow mobility), the diffusion model predicts outbreaks at equilibrium ($R_{0}^{D}>1$) while the simulated attack rate remains negligible (see Figs 2E, 3E and 4E). This discrepancy arises because the system fails to reach equilibrium within the two-year horizon, highlighting that *R*_0_ is not always a valid predictor over short- to medium-term time horizons. We therefore quantify the validity domain of *R*_0_-based predictions with a blue dotted line in Figs 2B, 2E, 2G, 3B, 3E, 3G, 4B, 4E and 4G. Only to the left of this line do outbreaks reach quasi-equilibrium within two years. In the vicinity of this boundary, the dynamics remain only partially developed within the analyzed time span (see Fig B in S1 Appendix). In contrast with the FoI, the addition of airline mobility and local vector interaction (see Eq. 4) leads to outbreaks even in the fast mobility regime, as imported infections can sustain local transmission, despite rapid host movement. Its effect is instead marginal where internal mobility alone already sustains transmission. Finally, for slow mobility, the diffusion model behaves similarly to the FoI although its predicts significantly larger outbreak sizes, consistent with previous analyses (see Figs 2G, 3G and 4G).The standard deviation of the attack rate for the synthetic networks ensemble is maximal at the transition since each network undergoes it for slightly different parameters (see Fig A in S1 Appendix).

#### Comparison between formulations.

Our analysis reveals that the FoI and diffusion models exhibit opposite responses to mobility, depending on the balance between human mobility (ϵ) and host-mediated vector mobility (*p*_0_). For any values of ϵ and *p*_0_, the mobility has no effect in the FoI model and systems remain below the epidemic threshold. In contrast, the diffusion model predicts that mobility can raise the systems above the threshold, enabling epidemic onset. For fast host mobility (ϵ≫1) and any proportion of vector travelers ($\epsilon \gg p_{0}$ or $\epsilon \ll p_{0}$), both models tend to homogenize the systems dynamics keeping it below threshold. For slow host mobility, models diverge (ϵ≫1): the mobility raises the systems above the epidemic threshold in the diffusion model whereas the systems remain subthreshold for the FoI model. These contrasting patterns arise because the models differ in how human populations are represented as the diffusion model incorporates an effective redistribution of humans across nodes leading to progressive changes in the vector-to-host ratio, while the FoI model assumes that mobility does not significantly affect the average local vector-to-host ratio.

Overall, the diffusion model shows that mobility features can shift systems across the epidemic threshold, underscoring the importance of the choice of the mobility formulation in predicting large-scale epidemic dynamics.

### Comparison between mobility formulations with fixed host population

While the previous analysis highlights important differences between the FoI and diffusion formulations, the use of the diffusion formulation may primarily apply to scenarios in which the human population physically relocates, such as seasonal or long-term migration. To further investigate the effects of population redistribution -and to address whether model differences persist when the host distribution is stable- we compare the two models while keeping the human population fixed. Specifically, we set the host population in each node equals to the equilibrium distribution obtained from the diffusion model, while leaving all other parameters unchanged. Under this setup, the distribution of local reproduction numbers becomes heterogeneous, reflecting conditions typical of the early phase of the peak season in non-endemic areas [2].

**Fig 5:**
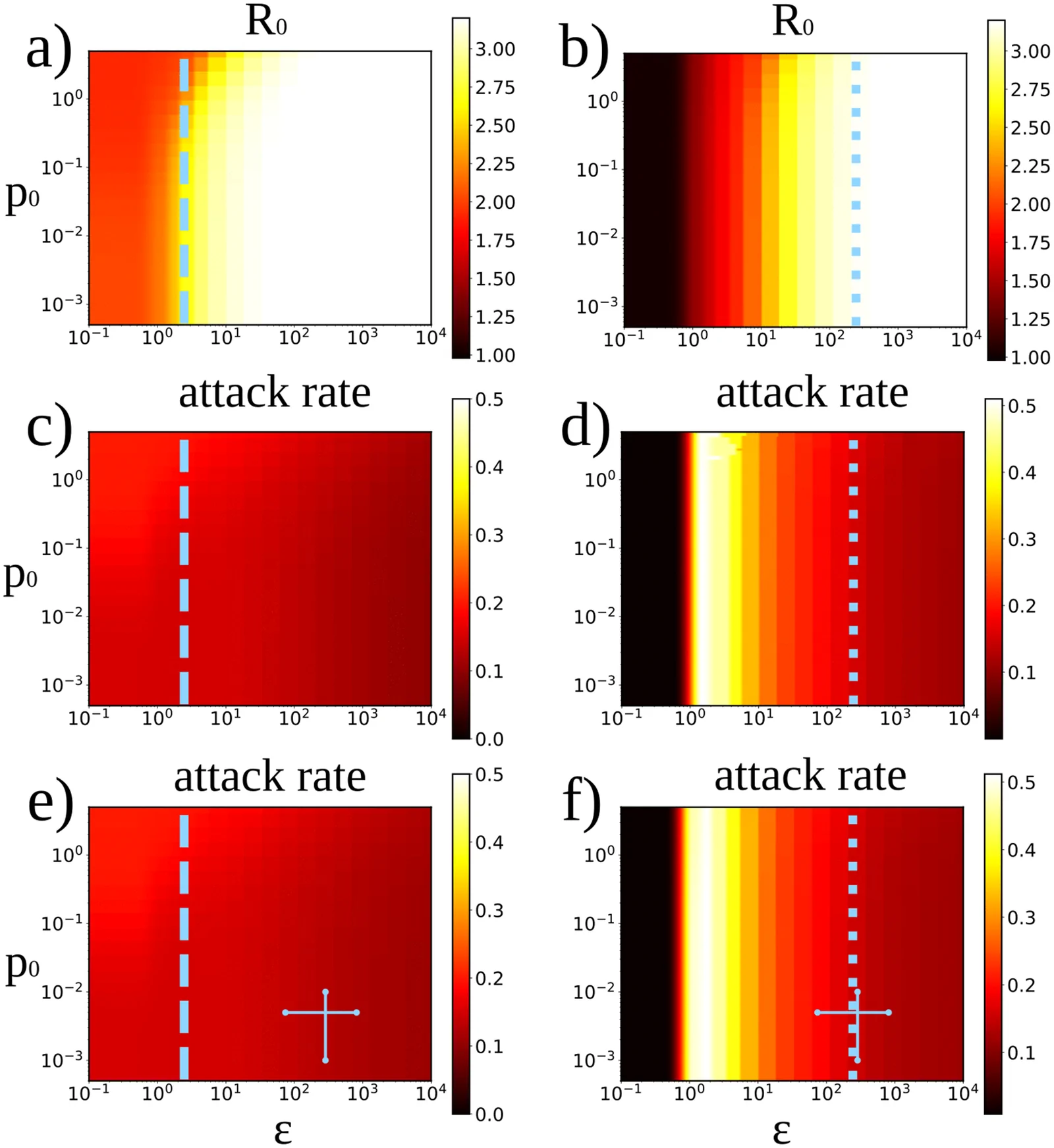
Phase diagrams in the ($\epsilon ,p_{0}$) space for Italian provinces (NUTS3) where host population is at equilibrium. *R*_0_ for the force of infection model **a)** and the diffusion model **b)**. Attack rate without infection reservoir for the force of infection model **c)** and for the diffusion model **d)**. Attack rate with infection reservoir for the force of infection model **e)** and for the diffusion model **f)**. ϵ is the variable associated with human mobility and *p*_0_ is the one associated with human-mediated vector mobility. Horizontal and vertical plain lines are the estimation of the uncertainty range for empirical ϵ and *p*_0_. The blue dashed line is the estimated threshold between slow and fast mobility. The blue dotted line corresponds to $\epsilon =\frac{t_{max}}{3}=243$, the limit after which the host mobility diffusion does not reach equilibrium. The host population of the FoI and diffusion model are constant equal to its equilibrium value for the diffusion model. $R_{0}^{W}=2$ for the infection reservoir.

**Case** ϵ≫1. In this regime, the basic reproduction numbers for the FoI and the diffusion model are identical with values consistent those obtained in the previous analysis ($R_{num}^{D}=3.24$ for Italian provinces). These results are in agreement with numerical simulations which show similar attack rates for both model, regardless of whether airline mobility is included (see Fig 5). This convergence is expected as, in the absence of host mobility, the models are mathematically equivalent.

**Case** ϵ≪1. In this regime, the basic reproduction numbers for the FoI and the diffusion model diverge. While the FoI model remains above threshold ($R_{num}^{F}\approx 2$ for the Italian provinces), the diffusion model falls below threshold (see Fig 5). This behavior is consistent with analytical predictions for this regime, as the reproduction number of the diffusion model does not explicitly depend on the equilibrium human population (see Text E in S1 Appendix for derivation). The corresponding attack rates confirm the difference between the two models. Mechanistically, the effective redistribution of the human population at equilibrium in the diffusion model causes the system to behave like a single, well-mixed patch, which in this case lies below the epidemic threshold.

**Case** ϵ~1.

In this intermediate regime, the basic reproduction number for the diffusion model is above threshold but consistently lower than that of the FoI model (see Fig 5). Despite this, the attack rate is substantially higher in the diffusion model. This discrepancy highlights a key mechanistic difference: the effective mobility of susceptible hosts, which is absent in the FoI model. In the diffusion model, susceptible individuals continuously move into nodes where transmission potential is high, sustaining the epidemic. At the same time, infected hosts also diffuse across the system maintaining epidemic growth in high-risk areas. In contrast, in the FoI model the pool of susceptible individuals is not replenished (aside from demographic processes) and progressively declines. As a result, although the FoI model exhibit a higher basic reproduction number, it leads to a lower overall attack rate. Similar results are observed at the regional scale (see Fig C in S1 Appendix).

Overall, while the two formulations yield comparable results under slow human mobility, their divergence at intermediate and high mobility underscores the importance of the mobility formulation in shaping large-scale epidemic dynamics, even when the host population is kept fixed.

### Importation risk of vector-borne diseases in real-world conditions

To further assess the comparative impact of mobility parameters in the two formulations, we estimate the local importation risk of vector-borne diseases under real-world conditions. In particular, we evaluate how the models’ assumptions shape the interplay between different mobility pathways using data-driven parameter values.

First, we estimate the time-scale separation parameter, and the human-mediated vector mobility parameter for the Italian networks. We interpret the latter as the average number of *Aedes albopictus* mosquitoes transported per human trip between nodes. Based on empirical studies including observations from Barcelona reporting values between 0.002 and 0.01, we fix this parameter to *p*_0_ = 0.005 [49] (see Supplementary Material). While the two models differ in how mobility affects epidemic dynamics, they rely on the same underlying host mobility representation, and thus share a consistent definition of the time-scale separation parameter. Using time-resolved mobility data, we estimate this parameter across nodes and time intervals (see Methods). Negative values reflect flows from smaller to larger cities, such as return mobility after summer holidays. To ensure consistency, we consider both absolute and positive-only distributions (see Fig D in S1 Appendix). For Italian regions, the average of the medians yields a representative value of 390, with an uncertainty interval from 151 to 1514. For Italian provinces, the corresponding value is 285, with an uncertainty interval from 74 to 812. These relatively high values indicate a slow diffusion regime, consistent with observed summer mobility patterns in Italy.

Next, we focus on the Italian provinces to investigate how the mobility formulation shapes the estimation of the local importation risk in a realistic setting. We define the local importation risk as the probability for each province to be infected at a given time knowing a particular initial condition. We consider that an infection in a province can be detected only when at least two infections occur (see Methods for details). We assume the local *R*_0_ = 2 for each province which is consistent to the observed basic reproduction number during the peak season on a historical chikungunya outbreak in Italy [7]. We fixed the total rate of infected international travelers injected in Italy to 0.01 ${week}^{-1}$ and we set the mobility parameters to ϵ=285 and *p*_0_ = 0.005. Epidemic parameters are calibrated for chikungunya (see Table A in S1 Appendix) (see Methods for details).

**Fig 6:**
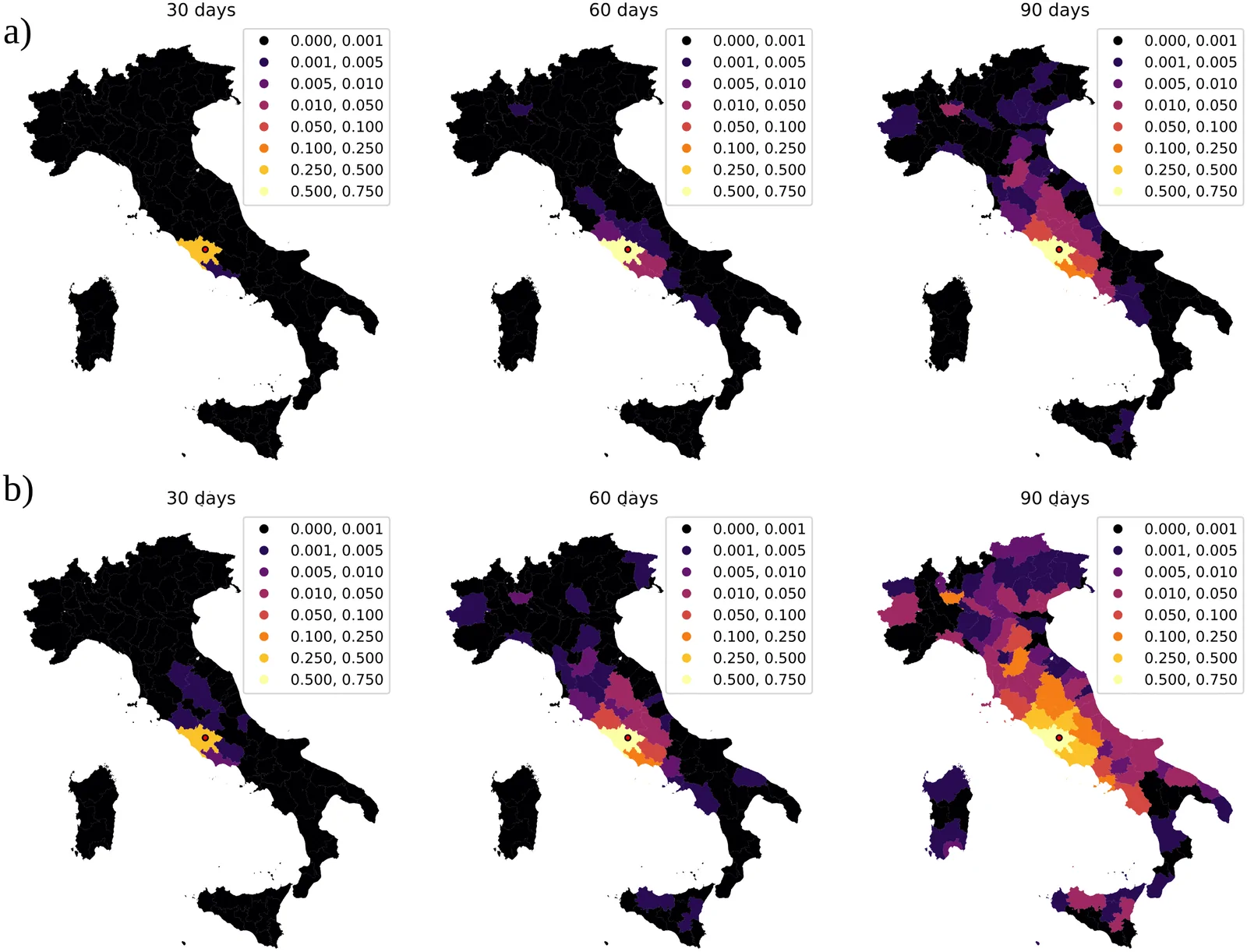
Local importation risk for Italian provinces (NUTS3) in function of time knowing that the infection is seeded in Rome province. Probability for a province to be infected 30, 60 and 90 days after the arrival of an infected individual in Rome for the **a)** FoI model and the **b)** diffusion model. *R*_0_ = 2 for each province. Fixed airline injection rate in Rome is 0.01 individual per week. Province is considered infected if at least 2 individuals are present. The red points indicate where the infection is seeded. Probabilities are computed over 1000 simulations. Basemap administrative boundaries are realized by ISTAT data, via the Openpolis geojson-italy repository under the Creative Commons Attribution 4.0 (CC BY 4.0) license.

First, we analyze a *monocentric* scenario where an infected individual is introduced in a province hosting an airport at time *t* = 0 and where the total rate of infected travelers is concentrated there. We apply the *monocentric* scenario on the four biggest international airports of Italy (see Methods): Rome, Milan, Bergamo and Venice. Results show that 30 days after the introduction of an infected individual in the Rome province, the probability of spillover to another province is below 1% for FoI and diffusion models (see Fig 6). In contrast, 4 provinces have a probability to be infected superior to 5% after 90 days in the FoI model, but only after 60 days in the diffusion model. In the same vein, results show that 13 provinces have a probability to be infected greater than 1% after 90 days in the FoI model while the very same provinces have probability to be infected greater than 5% in the diffusion model after 90 days.

**Fig 7:**
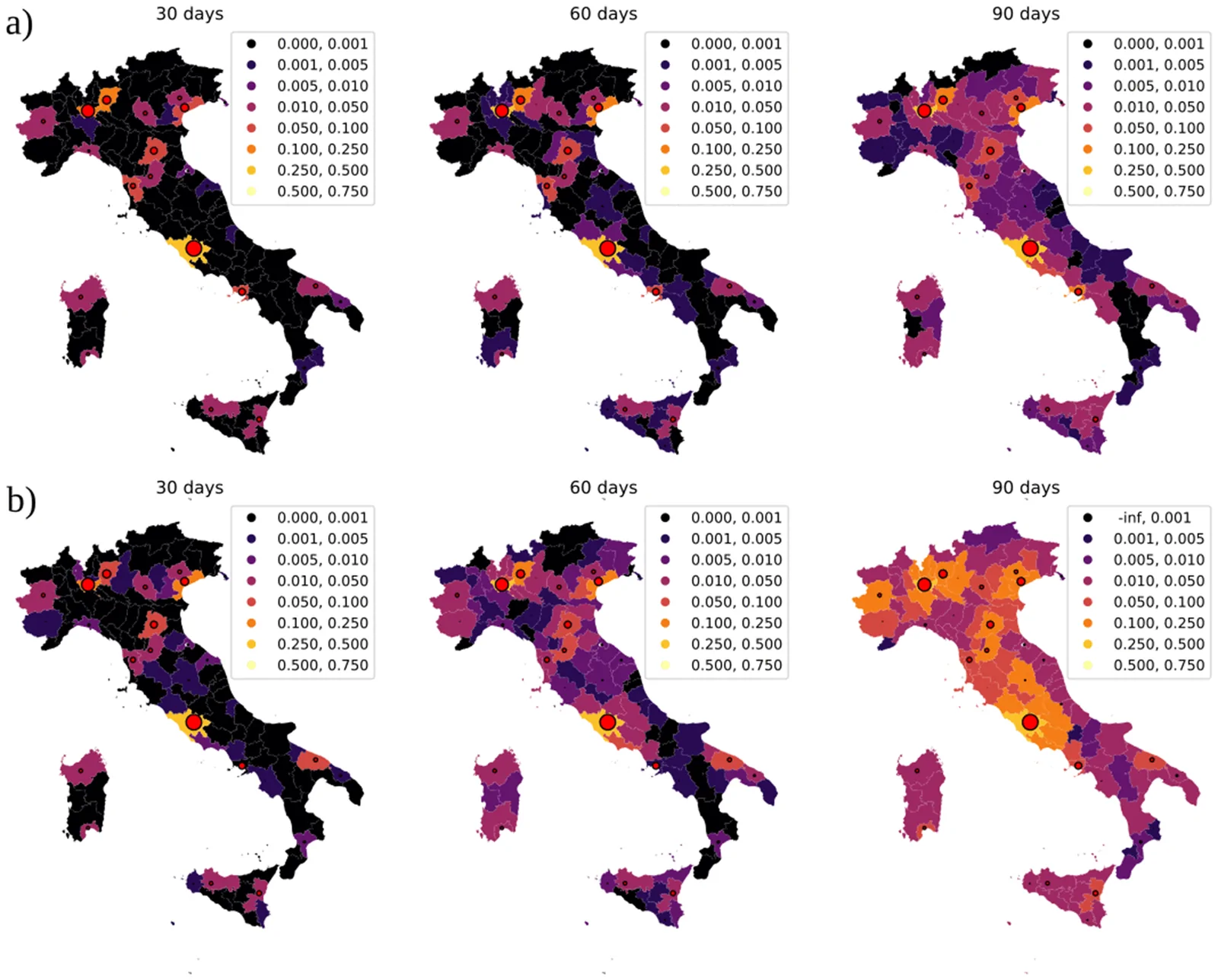
Local importation risk for Italian provinces (NUTS3) in function of time knowing that the infection is seeded proportionally to the incoming international airline flow. Probability for a province to be infected 30,60 and 90 days after the arrival of an infected individual for the **a)** FoI model and the **b)** diffusion model. Fixed airline injection rate is 0.01 people per week distributed across the international airports according to their weight. A province is considered infected if at least 2 individuals are present. Red points indicate the presence of an international airport and their size is proportional to their weight in the incoming international airline flow. Probabilities are computed over 1000 simulations. Basemap administrative boundaries are realized by ISTAT data, via the Openpolis geojson-italy repository under the Creative Commons Attribution 4.0 (CC BY 4.0) license.

While the FoI and the diffusion model follow very similar spatio-temporal patterns of infection spreading at short time (before 30 days), the diffusion model leads to significantly shorter arrival times than the FoI model at longer time (after 30 days). Note that the diffusion model always conserves similar patterns as the FoI model. Similar results are found when the infection is seeded in Milan (Fig E in S1 Appendix), Bergamo (Fig F in S1 Appendix) or Venice (Fig G in S1 Appendix). Then, we analyze a *polycentric* scenario where the infected individual is introduced in a province *i* according to $T_{i}^{W}$, the proportion it represents in the Italian inflow of international passengers. The fixed rate 0.01 infected passengers per week is distributed accordingly. Results show that there are 7 provinces with probabilities of being infected superior to 5% after 60 days in the FoI model corresponding to the biggest airports cumulating around 80% of the international passengers inflow (see Fig 7). In contrast, 10 provinces have corresponding probabilities in the diffusion model, among which three do not host international airports but are direct neighbors of provinces hosting big international airports. At 90 days, there are 10 provinces in the FoI model and 45 provinces in the diffusion model with probabilities of being infected superior to 5%.

Both models exhibit similar spatial patterns where the most important risk of spillover is concentrated in provinces neighboring the ones hosting an international airport. Strong mobility interaction between neighboring provinces can explain this effect (see Fig 4A). By computing the odds ratio between the diffusion and FoI models (see Fig H in S1 Appendix), we show again that the estimated importation risk for a given province is much more important in the diffusion model than in the FoI model at longer time horizons.

We hypothesize that the effective movement of infected hosts, combined with the replenishment of susceptible individuals described in the previous analysis, explains the faster spread observed in the diffusion model compared with the Fol one. Overall, these results confirm, using data-driven mobility parameters, that the choice of mobility formulation has a crucial impact on how the interplay between mobility pathways translates into epidemic outcomes in realistic settings.

## Discussion

In this study, we analyzed how different formulations of host mobility shape the predicted dynamics of vector-borne diseases in non-endemic regions. Using unified metapopulation models, we compared two classical formulations: a force-of-infection (FoI) formulation, in which human movement is implicit, and a diffusion formulation, in which individuals move explicitly between nodes. Unlike most previous models that account only for human movement, they integrate four coupled mobility processes: host mobility within countries and long-range air traffic, autonomous vector dispersal and host-mediated vector transport.

The results underscore how different modeling formulations can lead to divergent epidemic outcomes, even when based on the same empirical data. First, in the diffusion model, the interplay between host and vector mobility can generate spontaneous large-scale outbreaks, even when the system is initially subthreshold (*R*_0_ < 1). This occurs because explicit host diffusion progressively alters local vector-to-host ratio over time, an important variable in basic reproduction number of vector-borne disease [53], effectively pushing some patches above the epidemic threshold. Moreover, the movement of infected hosts actively transports the pathogen to new locations, where they act as persistent sources of infection for local vectors, intensifying transmission in newly reached areas. At the same time, the mobility of susceptible hosts into supra-threshold nodes replenishes the local susceptible pool, sustaining large outbreaks in intermediate mobility regime within the diffusion model.

In contrast, such mobility-driven transitions cannot occur in the FoI model, as host movement is only implicit and temporary, leaving the vector-to-host ratio constant and preventing the redistribution of infected hosts across patches. In this case, only external seeding—for instance through airline importation—can trigger outbreaks in subthreshold systems, for slow host and vector mobility. These findings hold consistently across both synthetic networks and the empirical Italian mobility networks at regional (NUTS2) and provincial (NUTS3) scales. Altogether, it implies that the representation of host mobility can critically influence predicted outbreak likelihood, size and timing.

To test our results in a real-world setting, we focused on Italy by combining population, mobility and air traffic data to estimate host mobility (ϵ), host mediated vector mobility (*p*_0_) and airline mobility ($T_{W}$). Parameter estimates suggest a low host mobility rate ϵ highlighting that mobility and epidemics timescale are not identical as suggested by [18,55]. This relatively slow diffusion is consistent with the daily life observation that the flow of people between provinces is small relative to a physical diffusion. Although the estimated contribution of the host-mediated vector mobility *p*_0_ is small, the high volume of daily intra-country trips translates into potentially tens of thousands of transported mosquitoes, relevant at national scales even if negligible locally [49]. This illustrative setting demonstrates that the host mobility formulation substantially affects key estimates for epidemic preparedness. When airline seeding is fixed at a constant rate, both models predict negligible short-term spillover (within 30 days) but non-negligible long-term risk, with similar spatial patterns concentrated in provinces strongly connected to international airports. However, the diffusion model systematically predicts significantly higher long-term risk (90 days), particularly for values of the reproduction number associated with high local transmission potential. While a local reproduction number of 2 may be realistic over short periods, maintaining such values over 90 days represents an extreme scenario, although it may become plausible under future climate warming [7,66]. The divergence in epidemiological outcomes between the two models stems from their different mechanistic formulations of host movement. In the diffusion model, the higher risk is driven by the explicit relocation of both susceptible and infected individuals, who remain in new locations as persistent sources of infection for local vectors, thereby altering local transmission dynamics. As a result, diffusion-based formulations are better suited to describing sustained or long-term relocation processes, but may generate unrealistic short-term dynamics due to their memoryless nature, potentially overestimating the speed of outbreak spread. In contrast, the FoI formulation, which represents movement as an implicit mixing process, is more appropriate for capturing short-term mobility, although it may fail to represent mechanisms such as the recurrent visitation of hosts to specific locations. Consequently, researchers should explicitly evaluate and justify their mobility formulations based on the epidemiological context and the dynamics they aim to capture.

Our work presents the following limitations. A key limitation is that both models assume a constant basic reproduction number and time-invariant vector-abundance, thus overlooking seasonal fluctuations that can substantially affect transmission potential in real-world conditions [2,5,67]. Although this represents a strong simplification, the scenarios presented should be regarded as illustrative of eco-climatic conditions favorable to transmission. Diffusion formulations simplify human mobility as memoryless, thereby neglecting recurrent commuting patterns known to alter epidemic thresholds and spatial unfolding of the disease [20,22,46]. In particular, a diffusion model was shown to overestimate the empirical *R*_0_ in an endemic setting for malaria [21]. While the diffusion model may underestimate the importation time in non-endemic settings, the FoI one may overestimate it by constraining infected individuals from moving and rapidly initiating transmission elsewhere. Future work should incorporate commuting-based mobility kernels preserving home-destination structure and differentiate airport-to-home versus global mobility since both may be quite different. Moreover, local variability in vector abundance, temperature, and precipitation introduces strong spatial heterogeneity in transmission potential at small scale. Past Italian chikungunya outbreaks have emerged in coastal or peri-urban areas with higher vector densities [7,52,66]. Considering such fine-scale environmental and demographic heterogeneity, alongside high-resolution municipal mobility data, could yield spatio-temporal patterns that differ from the one we investigated.

Despite these limitations, our results underscore that the choice of host-mobility formulation is not a technical detail but a determinant of outbreak predictions. Model formulations must therefore be explicitly justified and problem-specific. For instance, the ability of the diffusion model to modify the local vector-to-host ratio may become realistic for exploring the effects of temporary but sustained host redistribution on epidemic dynamics (e.g., seasonal labor migrations, touristic stays or migration flows), although it may overestimate short-term mobility effects. In such case, mobility can effectively relocate infectious individuals for most or all of their infectious period, making the diffusion approximation more meaningful. In contrast, the Fol formulation is more appropriate in contexts where host movement is transient and the overall population structure remains effectively constant. More generally, systematically comparing mobility formulations under time-dependent and spatially heterogeneous transmission conditions, for example by incorporating climate-driven parameters [2,3,67–70], could improve our understanding of their respective strengths and limitations, ultimately enhancing our ability to predict and mitigate the emergence of vector-borne epidemics in newly exposed areas.

## Supporting information

S1 Appendix Supplementary results and derivations. (PDF)
