# Supplementary material for: How host mobility formulations shape estimates of pathogen dispersal and epidemic risk in non endemic regions
Source: PLoS Comput Biol. 2026 Aug 24;22(8):e1014646. doi: 10.1371/journal.pcbi.1014646 (PMC13533434; doi:10.1371/journal.pcbi.1014646)
Supplement: Supplementary file 1 [file pcbi.1014646.s001.pdf]

Supplementary Text

How host mobility formulations shape estimates of pathogen dispersal and epidemic risk in non endemic regions

Charley Presigny[1,2*], Piero Poletti[3], Stefano Merler[3] and Manlio De Domenico[1,2,4]

1 Department of Physics & Astronomy 'Galileo Galilei', University of Padua, Padua, Italy
2 Istituto Nazionale di Fisica Nucleare, Sez. Padova, Padua, Italy
3 Center for Health Emergencies, Fondazione Bruno Kessler, Trento, Italy
4 Padua Center for Network Medicine, University of Padua, Padua, Italy

* corresponding author: charleypierre.presigny@unipd.it

Contents

A Models formulation **2**
- A.1 Force of infection model . . . 2
  - A.1.1 ODEs . . . 2
  - A.1.2 Matrix formulation . . . 2
- A.2 Diffusion model . . . 3
  - A.2.1 ODEs . . . 3
  - A.2.2 Matrix formulation . . . 3

B Synthetic data **3**

C Next-generation matrices **5**
- C.1 Diffusion model . . . 5
- C.2 FoI model . . . 7

D Analysis of R_0 for the FoI model **8**
- D.1 Case $X \ll 1$ . . . 9
- D.2 Development of the matrices A and B . . . 9
  - D.2.1 Explicit form of the exact solution . . . 9
  - D.2.2 First order perturbation eigenvalue . . . 9
- D.3 Case $X \gg 1$ and $X_0 \ll 1$ . . . 10
- D.4 Case $X \gg 1$ . . . 11
- D.5 Transition between the regimes $X, X_0 \ll 1$ and $X, X_0 \gg 1$ . . . 11

E Analysis of R_0 for the diffusion model **12**
- E.1 Case $\epsilon \to 0$ . . . 12

A Models formulation

A.1 Force of infection model

A.1.1 ODEs

$$\frac{dS_h^i}{dt} = \frac{N_h^i - S_h^i}{T_{lh}} - \frac{S_h^i c_{vh}^i}{N_h^i} \sum_j I_v^j K_h^{ji} \tag{1}$$

$$\frac{dE_h^i}{dt} = \frac{S_h^i c_{vh}^i}{N_h^i} \sum_j I_v^j K_h^{ji} - E_h^i(\frac{1}{T_{iit}} + \frac{1}{T_{lh}}) \tag{2}$$

$$\frac{dI_h^i}{dt} = \frac{E_h^i}{T_{iit}} - I_h^i(\frac{1}{T_{id}} + \frac{1}{T_{lh}}) \tag{3}$$

$$\frac{dR_h^i}{dt} = \frac{I_h^i}{T_{id}} - \frac{R_h^i}{T_{lh}} \tag{4}$$

$$\frac{dS_v^i}{dt} = \frac{\kappa^i - S_v^i}{T_{lv}} - \frac{S_v^i c_{hv}^i}{N_h^i} \sum_j \left(I_h^j + \delta_{ij} N_W^{sys} T_i^W \frac{I_h^W}{N_h^W} \right) K_v^{ji} \tag{5}$$

$$\frac{dE_v^i}{dt} = \frac{S_v^i c_{hv}^i}{N_h^i} \sum_j \left(I_h^j + \delta_{ij} N_W^{sys} T_i^W \frac{I_h^W}{N_h^W} \right) K_v^{ji} - E_v^i(\frac{1}{T_{eit}} + \frac{1}{T_{lv}}) \tag{6}$$

$$\frac{dI_v^i}{dt} = \frac{E_v^i}{T_{eit}} - \frac{I_v^i}{T_{lv}} \tag{7}$$

A.1.2 Matrix formulation

$$\frac{d\mathbf{S_h}}{dt} = \left(\frac{\mathbf{N_h} - \mathbf{S_h}}{T_{lh}} \right) - \boldsymbol{\mathcal{C}_{vh}}(\boldsymbol{\mathcal{K}_h}^\top \mathbf{I_v} \odot \mathbf{S_h}) \tag{8}$$

$$\frac{d\mathbf{E_h}}{dt} = \boldsymbol{\mathcal{C}_{vh}}(\boldsymbol{\mathcal{K}_h}^\top \mathbf{I_v} \odot \mathbf{S_h}) - \left(\frac{T_{lh} + T_{iit}}{T_{lh} T_{iit}} \right) \mathbf{E_h} \tag{9}$$

$$\frac{d\mathbf{I_h}}{dt} = \frac{\mathbf{E_h}}{T_{iit}} - \left(\frac{T_{lh} + T_{id}}{T_{lh} T_{id}} \right) \mathbf{I_h} \tag{10}$$

$$\frac{d\mathbf{R_h}}{dt} = \frac{\mathbf{I_h}}{T_{id}} - \frac{\mathbf{R_h}}{T_{lh}} \tag{11}$$

$$\frac{d\mathbf{S_v}}{dt} = \left(\frac{\boldsymbol{\kappa} - \mathbf{S_v}}{T_{lv}} \right) - \boldsymbol{\mathcal{C}_{hv}}(\boldsymbol{\mathcal{K}_v}^\top \mathbf{I_h} \odot \mathbf{S_v}) \tag{12}$$

$$\frac{d\mathbf{E_v}}{dt} = \boldsymbol{\mathcal{C}_{hv}}(\boldsymbol{\mathcal{K}_v}^\top \mathbf{I_h} \odot \mathbf{S_v}) - \left(\frac{T_{lv} + T_{eit}}{T_{lv} T_{eit}} \right) \mathbf{E_v} \tag{13}$$

$$\frac{d\mathbf{I_v}}{dt} = \frac{\mathbf{E_v}}{T_{eit}} - \frac{\mathbf{I_v}}{T_{lv}} \tag{14}$$

A.2 Diffusion model

A.2.1 ODEs

$$\frac{dS_h^i}{dt} = \frac{N_h^i - S_h^i}{T_{lh}} - \frac{S_h^i c_{vh}^i}{N_h^i} \sum_j I_v^j K_h^{ji} + \frac{1}{\epsilon} \left[\sum_{j=1}^{M} P_{ji} S_j - P_{ij} S_i \right] \tag{15}$$

$$\frac{dE_h^i}{dt} = \frac{S_h^i c_{vh}^i}{N_h^i} \sum_j I_v^j K_h^{ji} - E_h^i \left(\frac{1}{T_{iit}} + \frac{1}{T_{lh}}\right) + \frac{1}{\epsilon} \left[\sum_{j=1}^{M} P_{ji} E_j - P_{ij} E_i \right] \tag{16}$$

$$\frac{dI_h^i}{dt} = \frac{E_h^i}{T_{iit}} - I_h^i \left(\frac{1}{T_{id}} + \frac{1}{T_{lh}}\right) + \frac{1}{\epsilon} \left[\sum_{j=1}^{M} P_{ji} I_j - P_{ij} I_i \right] \tag{17}$$

$$\frac{dR_h^i}{dt} = \frac{I_h^i}{T_{id}} - \frac{R_h^i}{T_{lh}} + \frac{1}{\epsilon} \left[\sum_{j=1}^{M} P_{ji} R_j - P_{ij} R_i \right] \tag{18}$$

$$\frac{dS_v^i}{dt} = \frac{\kappa^i - S_v^i}{T_{lv}} - \frac{S_v^i c_{hv}^i}{N_h^i} \sum_j \left(I_h^j + \delta_{ij} N_W^{sys} T_i^W \frac{I_h^W}{N_h^W} \right) K_v^{ji} \tag{19}$$

$$\frac{dE_v^i}{dt} = \frac{S_v^i c_{hv}^i}{N_h^i} \sum_j \left(I_h^j + \delta_{ij} N_W^{sys} T_i^W \frac{I_h^W}{N_h^W} \right) K_v^{ji} - E_v^i \left(\frac{1}{T_{eit}} + \frac{1}{T_{lv}}\right) \tag{20}$$

$$\frac{dI_v^i}{dt} = \frac{E_v^i}{T_{eit}} - \frac{I_v^i}{T_{lv}} \tag{21}$$

A.2.2 Matrix formulation

$$\frac{d\mathbf{S_h}}{dt} = \left(\frac{\mathbf{N_h} - \mathbf{S_h}}{T_{lh}} \right) - \boldsymbol{\mathcal{C}_{vh}} \boldsymbol{\mathcal{K}_h}^\top \mathbf{I_v} \odot \mathbf{S_h} - \frac{1}{\epsilon} \boldsymbol{\mathcal{L}}^\top \mathbf{S_h} \tag{22}$$

$$\frac{d\mathbf{E_h}}{dt} = \boldsymbol{\mathcal{C}_{vh}} \boldsymbol{\mathcal{K}_h}^\top \mathbf{I_v} \odot \mathbf{S_h} - \left(\frac{T_{lh} + T_{iit}}{T_{lh} T_{iit}} + \frac{1}{\epsilon} \boldsymbol{\mathcal{L}}^\top \right) \mathbf{E_h} \tag{23}$$

$$\frac{d\mathbf{I_h}}{dt} = \frac{\mathbf{E_h}}{T_{iit}} - \left(\frac{T_{lh} + T_{id}}{T_{lh} T_{id}} + \frac{1}{\epsilon} \boldsymbol{\mathcal{L}}^\top \right) \mathbf{I_h} \tag{24}$$

$$\frac{d\mathbf{R_h}}{dt} = \frac{\mathbf{I_h}}{T_{id}} - \frac{\mathbf{R_h}}{T_{lh}} - \frac{1}{\epsilon} \boldsymbol{\mathcal{L}}^\top \mathbf{R_h} \tag{25}$$

$$\frac{d\mathbf{S_v}}{dt} = \left(\frac{\boldsymbol{\kappa} - \mathbf{S_v}}{T_{lv}} \right) - \boldsymbol{\mathcal{C}_{hv}} \boldsymbol{\mathcal{K}_v}^\top \mathbf{I_h} \odot \mathbf{S_v} \tag{26}$$

$$\frac{d\mathbf{E_v}}{dt} = \boldsymbol{\mathcal{C}_{hv}} \boldsymbol{\mathcal{K}_v}^\top \mathbf{I_h} \odot \mathbf{S_v} - \left(\frac{T_{lv} + T_{eit}}{T_{lv} T_{eit}} \right) \mathbf{E_v} \tag{27}$$

$$\frac{d\mathbf{I_v}}{dt} = \frac{\mathbf{E_v}}{T_{eit}} - \frac{\mathbf{I_v}}{T_{lv}} \tag{28}$$

B Synthetic data

Nodes of our synthetic networks are spatially distributed according to the Soneira-Peebles model,originally designed for the distribution of galaxies in the universe [1]. This model has the advantages of proposing a analytical two-points correlation function, i.e. the excess probability of finding two nodes separated by a given distance as compared with an uniform distribution, which is a power-law from a given resolution. It is driven by three parameters L, η, λ that determine together the number of nodes and their spatial

Ae. albopictus	Parameter	Baseline	Range	References
Demographics	T_{lh}	29220 (80 years)		Life expectancy (Greece, Italy, Spain, France)
	T_{lv}	13 (T_dep)	7-42	[5–9]
	M	2 (Tdep)	1-10	Mosquito over host ratio [6, 7]
	K_i	$M * Nh_i$		Carrying capacity
Mobility	η	150	110-150 m	computed from [9, 10]
	p_0	0.005	0.002-0.01	[11]
	ϵ	/	/	
DENV	T_{eit}	10 (T_dep)	7-14	[6, 9, 12, 13]
	T_{iit}	5	4-7	[6, 12, 13]
	T_{id}	5	2-12	[6, 9, 12, 13]
	c_{vh}	0.08	0.03-0.75	[6, 9, 12]
	c_{hv}	0.08	0.05-0.375	[6, 9, 12]
CHKV	T_{eit}	3	2-6	[6, 14, 15]
	T_{iit}	3	2-4	[6, 9, 14]
	T_{id}	6	2-7	[6, 9, 15]
	c_{vh}	0.05	0.03-0.08	[6, 9]
	c_{hv}	0.05	0.042-0.08	[6, 9]

Table A. Values of the biological parameters associated with dengue (DENV) and chikungunya (CHKV) for *Aedes albopictus*.

distribution [1]. Here, we first generate networks with 1000 nodes with parameters $L = 10, \eta = 2, \lambda = 1.59$ in a square with sides 1000 km. Then we attribute randomly to each node a population according to a Zipf-law of the form $P(r) = s * r^{-1}$, with r the rank of the node and $s = 1e6$ the population of the biggest node. This population distribution is consistent with the literature on human settlements that claim that the population distribution of the cities of a country follows a Zipf law, at least in its upper-tail part [2,3]. We associate airports to the 50 biggest nodes in terms of populations where daily arrival of passengers are distributed according to the Zipf-law with $s = 5500$, the rank of the airport matching the rank of the related node (e.g. the biggest airport with the biggest node). Altogether, nodes constitute synthetic cities in a country. Then, we operate a spatial hierarchical clustering to generate synthetic networks with $N = 100$ nodes accounting for synthetic NUTS3 units. Finally we build the mobility matrix between nodes using the radiation model [4].

C Next-generation matrices

C.1 Diffusion model

To compute the basic reproduction number R_0 which is defined as the "number of secondary cases produced in one generation in a fully susceptible population", we need to define the disease free equilibrium (DFE) $\mathbf{X}_s \in \mathbb{R}^{7N_{patch}}$ with $\mathbf{X}_s^i = (S_{heq}^i, 0, 0, 0, \kappa^i, 0, 0)^\top$ with $S_{veq}^i = \kappa^i$ and $S_{heq}^i = N_{heq}^i$. We consider a time $t >> T_{lv}, \epsilon$ to reach the equilibrium in population in each path.

The dynamical system can be written with a vector $\mathcal{F}, \mathcal{V} \in \mathbb{R}^{7N_{patch}}$ encompassing the rate of new infections in each compartment and the differences of rates of other transitions respectively between compartments (keep the order of the ODE) with $\frac{dX^i}{dt} = \mathcal{F}^i - \mathcal{V}^i$. For the sake of clarity, we will focus on the part of those vectors representing the infected compartments which are (E_h, I_h, E_v, I_v) using the matrix form using this very order:

$$\mathcal{F} = \begin{pmatrix} \boldsymbol{\mathcal{C}_{vh}}\boldsymbol{\mathcal{K}_h}^\top \mathbf{I_v} \odot \mathbf{S_h} \\ 0 \\ \boldsymbol{\mathcal{C}_{hv}}\boldsymbol{\mathcal{K}_v}^\top \mathbf{I_h} \odot \mathbf{S_v} \\ 0 \end{pmatrix} \quad \mathcal{V} = \begin{pmatrix} \left(\frac{\mathbf{I}}{T_{lh}} + \frac{\mathbf{I}}{T_{iit}} + \frac{1}{\epsilon}\mathcal{L}^\top\right)\mathbf{E_h} \\ -\frac{\mathbf{E_h}}{T_{iit}} + \left(\frac{\mathbf{I}}{T_{lh}} + \frac{\mathbf{I}}{T_{id}} + \frac{1}{\epsilon}\mathcal{L}^\top\right)\mathbf{I_h} \\ \left(\frac{\mathbf{I}}{T_{lv}} + \frac{\mathbf{I}}{T_{eit}}\right)\mathbf{E_v} \\ -\frac{\mathbf{E_v}}{T_{eit}} + \frac{\mathbf{I_v}}{T_{lv}} \end{pmatrix} \tag{29}$$

To compute R_0 through the next-generation matrix we compute the jacobian for the infected states $\mathcal{J} = \mathbf{F} - \mathbf{V}$ with $\mathbf{F}$ the derivatives of $\mathcal{F}$ and $\mathbf{V}$, the derivatives of V . The jacobian is expressed in the DFE point $\mathbf{X}_s$:

$$\mathbf{F}\Big|_{\mathcal{X}_s} = \begin{pmatrix} \mathbf{0} & \mathbf{0} & \mathbf{0} & \boldsymbol{\mathcal{C}'_{vh}}\boldsymbol{\mathcal{K}_h}^\top \\ \mathbf{0} & \mathbf{0} & \mathbf{0} & \mathbf{0} \\ \mathbf{0} & \boldsymbol{\mathcal{C}_{hv}}(\boldsymbol{\mathcal{K}_v}^\top \odot \boldsymbol{\kappa}^\top) & \mathbf{0} & \mathbf{0} \\ \mathbf{0} & \mathbf{0} & \mathbf{0} & \mathbf{0} \end{pmatrix} \tag{30}$$

$$\mathbf{V}\Big|_{\mathcal{X}_s} = \begin{pmatrix} \left(\frac{\mathbf{I}}{T_{lh}} + \frac{\mathbf{I}}{T_{iit}} + \frac{1}{\epsilon}\mathcal{L}^\top\right) & \mathbf{0} & \mathbf{0} & \mathbf{0} \\ -\frac{\mathbf{I}}{T_{iit}} & \left(\frac{\mathbf{I}}{T_{lh}} + \frac{\mathbf{I}}{T_{id}} + \frac{1}{\epsilon}\mathcal{L}^\top\right) & \mathbf{0} & \mathbf{0} \\ \mathbf{0} & \mathbf{0} & \left(\frac{\mathbf{I}}{T_{lv}} + \frac{\mathbf{I}}{T_{eit}}\right) & \mathbf{0} \\ \mathbf{0} & \mathbf{0} & -\frac{\mathbf{I}}{T_{eit}} & \frac{\mathbf{I}}{T_{lv}} \end{pmatrix} \tag{31}$$

with $\boldsymbol{\mathcal{C}'_{vh}} = diag(c_{vh}^1, ..., c_{vh}^j, ...)$,$\boldsymbol{\mathcal{C}_{hv}} = diag(\frac{c_{hv}^1}{N_h^1}, ..., \frac{c_{hv}^j}{N_h^j}, ...)$,$\boldsymbol{\kappa} = (\kappa^1, ...\kappa^i, ...)$, $\mathbf{0}$ a $N_{patch} \times N_{patch}$ null matrix and $\mathbf{I}$ the unit matrix. The next-generation matrix is defined as $\mathbf{K} = \mathbf{F}\mathbf{V}^{-1}$ [16]. To compute $\mathbf{V}^{-1}$, we remark that this matrix is diagonal per blocks.Therefore, it will be composed of the inverse of the diagonal blocks on its diagonal. We also remark that both diagonal blocks have the form of a lower-triangular matrix per

block. Yet, the inverse of a lower-triangular matrix per block has the form ([17]p146):

$$\begin{bmatrix} A & 0 \\ C & D \end{bmatrix}^{-1} = \begin{bmatrix} A^{-1} & 0 \\ -D^{-1}CA^{-1} & D^{-1} \end{bmatrix}, \tag{32}$$

with A,D invertible. Using the above formula $\mathbf{V}^{-1}$ read:

$$\mathbf{V}^{-1} = \begin{pmatrix} \left(\frac{T_{iit}+T_{lh}}{T_{iit}T_{lh}}\mathbf{I} + \frac{1}{\epsilon}\boldsymbol{\mathcal{L}}^\top\right)^{-1} & \mathbf{0} & \mathbf{0} & \mathbf{0} \\ \frac{1}{T_{iit}}\left(\frac{T_{id}+T_{lh}}{T_{id}T_{lh}}\mathbf{I} + \frac{1}{\epsilon}\boldsymbol{\mathcal{L}}^\top\right)^{-1}\left(\frac{T_{iit}+T_{lh}}{T_{iit}T_{lh}}\mathbf{I} + \frac{1}{\epsilon}\boldsymbol{\mathcal{L}}^\top\right)^{-1} & \left(\frac{T_{id}+T_{lh}}{T_{id}T_{lh}}\mathbf{I} + \frac{1}{\epsilon}\boldsymbol{\mathcal{L}}^\top\right)^{-1} & \mathbf{0} & \mathbf{0} \\ \mathbf{0} & \mathbf{0} & \frac{T_{eit}T_{lv}}{T_{eit}+T_{lv}}\mathbf{I} & \mathbf{0} \\ \mathbf{0} & \mathbf{0} & \frac{T_{lv}^2}{T_{eit}+T_{lv}}\mathbf{I} & T_{lv}\mathbf{I} \end{pmatrix} \tag{33}$$

Which lead to the following expression for $\mathbf{K}$:

$$\mathbf{K} = \begin{pmatrix} \mathbf{0} & \mathbf{0} & \boldsymbol{\mathcal{C}'}_{\boldsymbol{vh}}\boldsymbol{\mathcal{K}}_{\boldsymbol{h}}^\top\frac{T_{lv}^2}{T_{eit}+T_{lv}}\mathbf{I} & T_{lv}\boldsymbol{\mathcal{C}'}_{\boldsymbol{vh}}\boldsymbol{\mathcal{K}}_{\boldsymbol{h}}^\top \\ \mathbf{0} & \mathbf{0} & \mathbf{0} & \mathbf{0} \\ \boldsymbol{\mathcal{C}}_{\boldsymbol{hv}}(\boldsymbol{\mathcal{K}}_{\boldsymbol{v}}^\top \odot \boldsymbol{\kappa})\frac{1}{T_{iit}}\left(\frac{T_{id}+T_{lh}}{T_{id}T_{lh}}\mathbf{I} + \frac{1}{\epsilon}\boldsymbol{\mathcal{L}}^\top\right)^{-1}\left(\frac{T_{iit}+T_{lh}}{T_{iit}T_{lh}}\mathbf{I} + \frac{1}{\epsilon}\boldsymbol{\mathcal{L}}^\top\right)^{-1} & \boldsymbol{\mathcal{C}}_{\boldsymbol{hv}}(\boldsymbol{\mathcal{K}}_{\boldsymbol{v}}^\top \odot \boldsymbol{\kappa})\left(\frac{T_{id}+T_{lh}}{T_{id}T_{lh}}\mathbf{I} + \frac{1}{\epsilon}\boldsymbol{\mathcal{L}}^\top\right)^{-1} & \mathbf{0} & \mathbf{0} \\ \mathbf{0} & \mathbf{0} & \mathbf{0} & \mathbf{0} \end{pmatrix} \tag{34}$$

The matrix $\mathbf{K}$ is antidiagonal per block. It means that $\mathbf{K}^2$ is diagonal per block. In full generality the eigenvalues λ of $\mathbf{K}$ are complex. Since the elements of $\mathbf{K}$ are all real it means that the potential complex conjugate λ^* are also eigenvalues of $\mathbf{K}$. It implies that $\lambda\lambda^*$ (λ^2 if real) are the eigenvalues of $\mathbf{K}^2$. Ultimately, we are interested in the spectral radius of $\mathbf{K}$ i.e. the maximum of the complex modulus formed by its eigenvalues $\sqrt{\lambda\lambda^*}$ (or $abs(\sqrt{\lambda^2})$ if eigenvalues are real). Therefore knowing the eigenvalues of $\mathbf{K}^2$ give us directly the spectral radius of $\mathbf{K}$ up to the square root. Since,the matrix $\mathbf{K}$ is antidiagonal per block. It means that $\mathbf{K}^2$ is diagonal per block and has the form:

$$
\mathbf{K}^2 = \begin{pmatrix} \boldsymbol{\mathcal{C}} & \boldsymbol{\mathcal{X}} & \mathbf{0} & \mathbf{0} \\ \mathbf{0} & \mathbf{0} & \mathbf{0} & \mathbf{0} \\ \mathbf{0} & \mathbf{0} & \boldsymbol{\mathcal{D}} & \boldsymbol{\mathcal{Y}} \\ \mathbf{0} & \mathbf{0} & \mathbf{0} & \mathbf{0} \end{pmatrix} \tag{35}
$$

$$
\boldsymbol{\mathcal{C}} = \boldsymbol{\mathcal{C}'_{vh}} \boldsymbol{\mathcal{K}_h^\top} \frac{T_{lv}^2}{T_{eit}+T_{lv}} \mathbf{I} \boldsymbol{\mathcal{C}_{hv}} (\boldsymbol{\mathcal{K}_v^\top} \odot \boldsymbol{\kappa}^\top) \frac{1}{T_{iit}} \left(\frac{T_{id}+T_{lh}}{T_{id}T_{lh}} \mathbf{I} + \frac{1}{\epsilon} \boldsymbol{\mathcal{L}}^\top \right)^{-1} \left(\frac{T_{iit}+T_{lh}}{T_{iit}T_{lh}} \mathbf{I} + \frac{1}{\epsilon} \boldsymbol{\mathcal{L}}^\top \right)^{-1} \tag{36}
$$

$$
\boldsymbol{\mathcal{X}} = \boldsymbol{\mathcal{C}'_{vh}} \boldsymbol{\mathcal{K}_h^\top} \frac{T_{lv}^2}{T_{eit}+T_{lv}} \mathbf{I} \boldsymbol{\mathcal{C}_{hv}} (\boldsymbol{\mathcal{K}_v^\top} \odot \boldsymbol{\kappa}^\top) \left(\frac{T_{id}+T_{lh}}{T_{id}T_{lh}} \mathbf{I} + \frac{1}{\epsilon} \boldsymbol{\mathcal{L}}^\top \right)^{-1} \tag{37}
$$

$$
\boldsymbol{\mathcal{D}} = \boldsymbol{\mathcal{C}_{hv}} (\boldsymbol{\mathcal{K}_v^\top} \odot \boldsymbol{\kappa}^\top) \frac{1}{T_{iit}} \left(\frac{T_{id}+T_{lh}}{T_{id}T_{lh}} \mathbf{I} + \frac{1}{\epsilon} \boldsymbol{\mathcal{L}}^\top \right)^{-1} \left(\frac{T_{iit}+T_{lh}}{T_{iit}T_{lh}} \mathbf{I} + \frac{1}{\epsilon} \boldsymbol{\mathcal{L}}^\top \right)^{-1} \boldsymbol{\mathcal{C}'_{vh}} \boldsymbol{\mathcal{K}_h^\top} \frac{T_{lv}^2}{T_{eit}+T_{lv}} \mathbf{I} \tag{38}
$$

$$
\boldsymbol{\mathcal{Y}} = \boldsymbol{\mathcal{C}_{hv}} (\boldsymbol{\mathcal{K}_v^\top} \odot \boldsymbol{\kappa}^\top) \frac{1}{T_{iit}} \left(\frac{T_{id}+T_{lh}}{T_{id}T_{lh}} \mathbf{I} + \frac{1}{\epsilon} \boldsymbol{\mathcal{L}}^\top \right)^{-1} \left(\frac{T_{iit}+T_{lh}}{T_{iit}T_{lh}} \mathbf{I} + \frac{1}{\epsilon} \boldsymbol{\mathcal{L}}^\top \right)^{-1} T_{lv} \boldsymbol{\mathcal{C}'_{vh}} \boldsymbol{\mathcal{K}_h^\top} \tag{39}
$$

Therefore to find the eigenvalues of $\mathbf{K}^2$, we need to find the eigenvalues of the diagonal blocks. According to Schur formula we notice that the determinant of the block matrices $\mathcal{M}$ of interests can be written in the following way [18]:

$$
det(\mathcal{M} - \lambda \mathbf{I}) = \begin{vmatrix} \mathcal{A} - \lambda \mathcal{I} & \mathcal{B} \\ \mathbf{0} & -\lambda \mathcal{I} \end{vmatrix} \tag{40}
$$

$$
= det(\mathcal{A} - \lambda \mathcal{I}) det(-\lambda \mathcal{I}) \tag{41}
$$

Therefore the eigenvalues of $\mathbf{K}^2$ are the ones of $\mathcal{C}, \mathcal{D}$ and 0 with degeneracy $2N \times 2N$.

C.2 FoI model

By using the same DFE as before the matrix $\mathbf{F}$ and $\mathbf{V}$ are expressed as:

$$
\mathbf{F}\Big|_{\mathcal{X}_s} = \begin{pmatrix} \mathbf{0} & \mathbf{0} & \mathbf{0} & \boldsymbol{\mathcal{C}'_{vh}} \boldsymbol{\mathcal{K}_h^\top} \\ \mathbf{0} & \mathbf{0} & \mathbf{0} & \mathbf{0} \\ \mathbf{0} & \boldsymbol{\mathcal{C}_{hv}} (\boldsymbol{\mathcal{K}_v^\top} \odot \boldsymbol{\kappa}^\top) & \mathbf{0} & \mathbf{0} \\ \mathbf{0} & \mathbf{0} & \mathbf{0} & \mathbf{0} \end{pmatrix} \tag{42}
$$

$$
\mathbf{V}\Big|_{\mathcal{X}_s} = \begin{pmatrix} \left(\frac{\mathbf{I}}{T_{lh}} + \frac{\mathbf{I}}{T_{iit}} \right) & \mathbf{0} & \mathbf{0} & \mathbf{0} \\ -\frac{\mathbf{I}}{T_{iit}} & \left(\frac{\mathbf{I}}{T_{lh}} + \frac{\mathbf{I}}{T_{id}} \right) & \mathbf{0} & \mathbf{0} \\ \mathbf{0} & \mathbf{0} & \left(\frac{\mathbf{I}}{T_{lv}} + \frac{\mathbf{I}}{T_{eit}} \right) & \mathbf{0} \\ \mathbf{0} & \mathbf{0} & -\frac{\mathbf{I}}{T_{eit}} & \frac{\mathbf{I}}{T_{lv}} \end{pmatrix} \tag{43}
$$

with $\boldsymbol{\mathcal{C}'_{vh}} = diag(c^1_{vh}, ..., c^j_{vh}, ...)$, $\boldsymbol{\mathcal{C}_{hv}} = diag(\frac{c^1_{hv}}{N^1_h}, ..., \frac{c^j_{hv}}{N^j_h}, ...)$,$\mathbf{0}$ a $N_{patch} \times N_{patch}$ null matrix and $\mathbf{I}$, the associated unit matrix. The next-generation matrix is defined as $\mathbf{K} = \mathbf{F}\mathbf{V}^{-1}$. We note that the matrix $\mathbf{F}$ is the same for both models (see Eq.30) but the form of $\mathbf{V}$ differs. Following the same canvas as in the diffusion model case we obtain the expression of $\mathbf{K}$:

$$\mathbf{K} = \begin{pmatrix} \mathbf{0} & \mathbf{0} & \boldsymbol{\mathcal{C}'_{vh}}\boldsymbol{\mathcal{K}_h^\top} \frac{T_{lv}^2}{T_{eit}+T_{lv}}\mathbf{I} & T_{lv}\boldsymbol{\mathcal{C}'_{vh}}\boldsymbol{\mathcal{K}_h^\top}\mathbf{I} \\ \mathbf{0} & \mathbf{0} & \mathbf{0} & \mathbf{0} \\ \boldsymbol{\mathcal{C}_{hv}}(\boldsymbol{\mathcal{K}_v^\top} \odot \boldsymbol{\kappa}^\top)\left(\frac{T_{id}T_{lh}^2}{(T_{id}+T_{lh})(T_{iit}+T_{lh})}\mathbf{I}\right) & \boldsymbol{\mathcal{C}_{hv}}\boldsymbol{\mathcal{K}_v^\top} \odot \boldsymbol{\kappa}^\top\left(\frac{T_{id}T_{lh}}{T_{id}+T_{lh}}\mathbf{I}\right) & \mathbf{0} & \mathbf{0} \\ \mathbf{0} & \mathbf{0} & \mathbf{0} & \mathbf{0} \end{pmatrix} \tag{44}$$

We consider the square of the previous matrix. This lead to matrices $\boldsymbol{\mathcal{A}}, \boldsymbol{\mathcal{B}}$ whose eigenvalues give the spectral radius.

$$\mathbf{K}^2 = \begin{pmatrix} \boldsymbol{\mathcal{A}} & \boldsymbol{\mathcal{F}} & \mathbf{0} & \mathbf{0} \\ \mathbf{0} & \mathbf{0} & \mathbf{0} & \mathbf{0} \\ \mathbf{0} & \mathbf{0} & \boldsymbol{\mathcal{B}} & \boldsymbol{\mathcal{G}} \\ \mathbf{0} & \mathbf{0} & \mathbf{0} & \mathbf{0} \end{pmatrix} \tag{45}$$

$$\boldsymbol{\mathcal{A}} = \boldsymbol{\mathcal{C}'_{vh}}\boldsymbol{\mathcal{K}_h^\top}\frac{T_{lv}^2}{T_{eit}+T_{lv}}\boldsymbol{\mathcal{C}_{hv}}(\boldsymbol{\mathcal{K}_v^\top} \odot \boldsymbol{\kappa}^\top)\left(\frac{T_{id}T_{lh}^2}{(T_{id}+T_{lh})(T_{iit}+T_{lh})}\right) \tag{46}$$

$$\boldsymbol{\mathcal{B}} = \boldsymbol{\mathcal{C}_{hv}}(\boldsymbol{\mathcal{K}_v^\top} \odot \boldsymbol{\kappa}^\top)\left(\frac{T_{id}T_{lh}^2}{(T_{id}+T_{lh})(T_{iit}+T_{lh})}\right)C'_{vh}\mathcal{K}_h^\top\frac{T_{lv}^2}{T_{eit}+T_{lv}} \tag{47}$$

D Analysis of R_0 for the FoI model

For the FoI model, we need to find the spectral radius of $\mathcal{A}$ and $\mathcal{B}$ to find R_0:

$$\boldsymbol{\mathcal{A}} = \boldsymbol{\mathcal{C}'_{vh}}\boldsymbol{\mathcal{K}_h^\top}\boldsymbol{\mathcal{C}_{hv}}(\boldsymbol{\mathcal{K}_v^\top} \odot \boldsymbol{\kappa})\left(\frac{T_{id}T_{lh}^2T_{lv}^2}{(T_{id}+T_{lh})(T_{iit}+T_{lh})(T_{eit}+T_{lv})}\right) \tag{48}$$

$$\boldsymbol{\mathcal{B}} = \boldsymbol{\mathcal{C}_{hv}}(\boldsymbol{\mathcal{K}_v^\top} \odot \boldsymbol{\kappa})\boldsymbol{\mathcal{C}'_{vh}}\boldsymbol{\mathcal{K}_h^\top}\left(\frac{T_{id}T_{lh}^2T_{lv}^2}{(T_{id}+T_{lh})(T_{iit}+T_{lh})(T_{eit}+T_{lv})}\right) \tag{49}$$

, with the infection kernel having the following form in our case study (autonomous vector dispersal disregarded):

$$K^{\beta\alpha}(x) = \frac{\delta_{\alpha\beta} + xP_{\beta\alpha}}{1+x} \tag{50}$$

, with $x = X = \frac{1}{\epsilon}$ $x = X_0 = \frac{p_0}{\epsilon}$ for the host kernel and $x = 1$ for vector kernel,respectively.

D.1 Case $X \ll 1$

In the case $X \ll 1$ (when the system tends to no interactions), the kernels can be rewritten as:

$$\boldsymbol{K}^{\top}(X) = \boldsymbol{I} + \boldsymbol{X}(\boldsymbol{P}^{\top} - \boldsymbol{I})(\boldsymbol{I} + \boldsymbol{X})^{-1} \tag{51}$$

$$= \boldsymbol{I} - \boldsymbol{X}\boldsymbol{\mathcal{L}}^{\top}(\boldsymbol{I} + \boldsymbol{X})^{-1} \tag{52}$$

Let us define:

$$\mathbf{D} = I \tag{53}$$

$$\boldsymbol{\mathcal{Y}}^{\top} = \boldsymbol{X}(\boldsymbol{P}^{\top} - \boldsymbol{I})(\boldsymbol{I} + \boldsymbol{X})^{-1} \tag{54}$$

$$\boldsymbol{\mathcal{Y}_{\wr}}^{\top} = \boldsymbol{X_0}(\boldsymbol{P}^{\top} - \boldsymbol{I})(\boldsymbol{I} + \boldsymbol{X_0})^{-1} \tag{55}$$

D.2 Development of the matrices A and B

From Eq. 48 the development of matrix read (the constants are skipped for readability):

$$\boldsymbol{\mathcal{A}} \sim \boldsymbol{\mathcal{C}'_{vh}}(\mathbf{D} + \boldsymbol{\mathcal{Y}}_{\mathbf{0}}^{\top})\boldsymbol{\mathcal{C}_{hv}}(\mathbf{D} + \boldsymbol{\mathcal{Y}}^{\top}) \odot \boldsymbol{\kappa}^{\top} \tag{56}$$

$$\sim \left(\underbrace{\boldsymbol{\mathcal{C}'_{vh}}\mathbf{D}\boldsymbol{\mathcal{C}_{hv}}\mathbf{D}}_{\boldsymbol{A_0}} + \underbrace{\boldsymbol{\mathcal{C}'_{vh}}(\mathbf{D}\boldsymbol{\mathcal{C}_{hv}}\boldsymbol{\mathcal{Y}}^{\top} + \boldsymbol{\mathcal{Y}}_{\mathbf{0}}^{\top}\boldsymbol{\mathcal{C}_{hv}}\mathbf{D})}_{\boldsymbol{A_1}} + \underbrace{\boldsymbol{\mathcal{C}'_{vh}}\boldsymbol{\mathcal{Y}}_{\mathbf{0}}^{\top}\boldsymbol{\mathcal{C}_{hv}}\boldsymbol{\mathcal{Y}}^{\top}}_{\boldsymbol{A_2}} \right) \odot \boldsymbol{\kappa}^{\top} \tag{57}$$

, with A_0, being the zeroth solution of the perturbation analysis, $\boldsymbol{A_1}$ and $\boldsymbol{A_2}$, the first order perturbation terms(that naturally appears).

$$\boldsymbol{\mathcal{B}} \sim \boldsymbol{\mathcal{C}_{hv}}(\mathbf{D} + \boldsymbol{\mathcal{Y}}^{\top}) \odot \boldsymbol{\kappa}^{\top}\boldsymbol{\mathcal{C}'_{vh}}(\mathbf{D} + \boldsymbol{\mathcal{Y}}_{\mathbf{0}}^{\top}) \tag{58}$$

$$\sim \boldsymbol{\mathcal{C}_{hv}}\mathbf{D} \odot \boldsymbol{\kappa}^{\top}\boldsymbol{\mathcal{C}'_{vh}}\mathbf{D} + \boldsymbol{C_{hv}}\boldsymbol{\mathcal{Y}}^{\top} \odot \boldsymbol{\kappa}^{\top}\boldsymbol{\mathcal{C}'_{vh}}\mathbf{D} + \boldsymbol{\mathcal{C}_{hv}}\mathbf{D} \odot \boldsymbol{\kappa}^{\top}\boldsymbol{\mathcal{C}'_{vh}}\boldsymbol{\mathcal{Y}}_{\mathbf{0}}^{\top} + \boldsymbol{\mathcal{C}_{hv}}\boldsymbol{\mathcal{Y}}^{\top} \odot \boldsymbol{\kappa}^{\top}\boldsymbol{\mathcal{C}'_{vh}}\boldsymbol{\mathcal{Y}}_{\mathbf{0}}^{\top} \tag{59}$$

D.2.1 Explicit form of the exact solution

It is easy to show that A_0 is a diagonal matrix whose diagonal elements are:

$$A_0^{ii} = \left(\frac{T_{id}T_{lh}^2 T_{lv}^2}{(T_{id} + T_{lh})(T_{iit} + T_{lh})(T_{eit} + T_{lv})} \right) \frac{c_{vh}^i c_{hv}^i \kappa_i}{N_h^i} \tag{60}$$

Since A_0 is diagonal, the diagonal elements are its eigenvalues whose eigenvectors forming the canonical basis $\{e_i\}$. Regarding only the matrix A_0 it leads to the following unperturbed R_0:

$$R_0 = \sqrt{\frac{T_{id}T_{lh}^2 T_{lv}^2}{(T_{id} + T_{lh})(T_{iit} + T_{lh})(T_{eit} + T_{lv})}} \max_i\left(\sqrt{\frac{c_{vh}^i c_{hv}^i \kappa_i}{N_h^i}}\right) \tag{61}$$

, which is consistent with the classical SEIR model with no mobility interaction [19].

D.2.2 First order perturbation eigenvalue

In the following we suppose $P_{ii} = 0$. All matrix but the mobility ones are diagonal. The first order perturbation eigenvalue as the form (constant not shown):

$$\lambda_1^i(\boldsymbol{\mathcal{A}}) \sim e_i^{\top} \left(\boldsymbol{\mathcal{C}'_{vh}}(\mathbf{D}\boldsymbol{\mathcal{C}_{hv}}\boldsymbol{\mathcal{Y}}^{\top} + \boldsymbol{\mathcal{Y}}_{\mathbf{0}}^{\top}\boldsymbol{\mathcal{C}_{hv}}\mathbf{D}) + \boldsymbol{\mathcal{C}'_{vh}}\boldsymbol{\mathcal{Y}}_{\mathbf{0}}^{\top}\boldsymbol{\mathcal{C}_{hv}}\boldsymbol{\mathcal{Y}}^{\top} \odot \boldsymbol{\kappa}^{\top} \right) e_i X X_0 \tag{62}$$

$$\sim \frac{(c_{vh}^i \sum_{l \neq i} (\boldsymbol{P}^{\top})_{il} \frac{c_{hv}^l \kappa^l}{N_h^l} (\boldsymbol{P}^{\top})_{li}) X X_0 - \frac{c_{vh}^i c_{hv}^i \kappa^i}{N_h^i}(X + X_0)}{(1 + X)(1 + X_0)} \tag{63}$$

At the first order it leads to:

$$\lambda^i(\boldsymbol{\mathcal{A}}) = \lambda_0^i(\boldsymbol{\mathcal{A}}) + \lambda_1^i(\boldsymbol{\mathcal{A}}) \tag{64}$$

$$= \frac{T_{id}T_{lh}^2 T_{lv}^2}{(T_{id}+T_{lh})(T_{iit}+T_{lh})(T_{eit}+T_{lv})}\left(\frac{\frac{c_{vh}^i c_{hv}^i \kappa^i}{N_h^i} + (c_{vh}^i \sum_{l\neq i}(\boldsymbol{P}^\top)_{il}\frac{c_{hv}^l \kappa^l}{N_h^l}(\boldsymbol{P}^\top)_{li})XX_0}{(1+X)(1+X_0)}\right) \tag{65}$$

For $\boldsymbol{\mathcal{B}}$, the first order eigenvalue read:

$$\lambda^i(\boldsymbol{\mathcal{B}}) \sim e_i^\top(\mathbf{D}+\boldsymbol{\mathcal{Y}}^\top)\boldsymbol{\mathcal{C}_{hv}}\odot\boldsymbol{\kappa}^\top\boldsymbol{\mathcal{C}'_{vh}}(\mathbf{D}+\boldsymbol{\mathcal{Y}_0}^\top)e_i \tag{66}$$

$$\sim \frac{\frac{c_{vh}^i c_{hv}^i \kappa^i}{N_h^i} + (\frac{c_{hv}^i \kappa^i}{N_h^i}\sum_{l\neq i}(\boldsymbol{P}^\top)_{il}c_{vh}^l(\boldsymbol{P}^\top)_{li})XX_0}{(1+X)(1+X_0)} \tag{67}$$

In the case of $\lambda^i(\boldsymbol{\mathcal{A}}) > \lambda^i(\boldsymbol{\mathcal{B}})$,we can say that R_0 at the first order read:

$$R_0^1 = max_i\left(\sqrt{\frac{T_{id}T_{lh}^2 T_{lv}^2}{(T_{id}+T_{lh})(T_{iit}+T_{lh})(T_{eit}+T_{lv})}\left(\frac{\frac{c_{vh}^i c_{hv}^i \kappa^i}{N_h^i} + (c_{vh}^i \sum_{l\neq i}(\boldsymbol{P}^\top)_{il}\frac{c_{hv}^l \kappa^l}{N_h^l}(\boldsymbol{P}^\top)_{li})XX_0}{(1+X)(1+X_0)}\right)}\right) \tag{68}$$

D.3 Case $X \gg 1$ and $X_0 \ll 1$

In the bottom left of the diagram (ϵ, p_0), the vector kernel is in the regime where $X \gg 1$ (full interaction) i.e. $\boldsymbol{\mathcal{K}_v^\top} = \mathbf{P}^\top$ and the host kernel is in the regime $X_0 \ll 1$ i.e. $\boldsymbol{\mathcal{K}_h^\top} = \mathbf{I}$. Therefore we can rewrite the matrix $\boldsymbol{\mathcal{A}}$ and $\boldsymbol{\mathcal{B}}$ as follows (constant terms are overlooked for the sake of clarity):

$$\boldsymbol{\mathcal{A}} \sim \boldsymbol{C'_{vh}}(\boldsymbol{C_{hv}}\mathbf{P}^\top \odot \boldsymbol{\kappa}^\top) \tag{69}$$

$$\boldsymbol{\mathcal{B}} \sim (\boldsymbol{C_{hv}}\mathbf{P}^\top \odot \boldsymbol{\kappa}^\top)\boldsymbol{C'_{vh}} \tag{70}$$

In terms of matrix element this reduces to:

$$\mathcal{A}_{ij} \sim \frac{c_{vh}^i c_{hv}^i \kappa^i}{N_h^i}P_{ji} \tag{71}$$

$$\mathcal{B}_{ij} \sim \frac{c_{hv}^i \kappa^i}{N_h^i}P_{ji}c_{vh}^j \tag{72}$$

In our case study $c_{vh}^i = c_{vh}$, $c_{hv}^i = c_{hv}$ and $\frac{\kappa_i}{N_h^i} = M$ $\mathcal{A}_{ij} \sim \mathcal{B}_{ij}$. It leads to $\mathcal{A}_{ij} \sim Mc_{vh}c_{hv}P_{ji} \ \sim (\mathbf{P}^\top)_{ij}$. Therefore in this particular case the matrix $\mathcal{A}$ has the same eigenvalue as $\mathbf{P}^\top$. Since $\mathbf{P}$ is a non-negative matrix, according to the Perron-Frobenius theorem the lower bond of its spectral radius $\rho(P)$ is $\rho(P) \leq max_i \sum_j P_{ij} = 1$. Actually it reaches 1. Since $\mathbf{P}^\top$ has the same eigenvalue as $\mathbf{P}$, the spectral radius of A is:

$$\rho(\boldsymbol{\mathcal{A}}) \sim Mc_{vh}c_{hv} \tag{73}$$

Adding the constants, we prove that under $X \gg 1$ and $X_0 \ll 1$, R_0 reduces to the one of the non-interacting case in our case study.

D.4 Case $X \gg 1$

In the case $X \gg 1$ and $X_0 \gg 1$ (when the system tends to full interactions), the kernels can be rewritten as $\mathcal{K}_h^\top = \mathbf{P}^\top$ and $\mathcal{K}_v^\top = \mathbf{P}^\top$. Therefore we can rewrite the matrix $\mathcal{A}$ and $\mathcal{B}$ as follows (constant terms are overlooked for the sake of clarity):

$$\mathcal{A} \sim \mathcal{C}'_{vh}\mathbf{P}^\top(\mathcal{C}_{hv}\mathbf{P}^\top \odot \kappa) \tag{74}$$

$$\mathcal{B} \sim (\mathcal{C}_{hv}\mathbf{P}^\top \odot \kappa)\mathcal{C}'_{vh}\mathbf{P}^\top \tag{75}$$

In terms of matrix element this reduces to:

$$\mathcal{A}_{ij} \sim c^i_{vh} \sum_k \frac{P_{ki}P_{jk}c^k_{hv}\kappa^k}{N^k_h} \tag{76}$$

$$\mathcal{B}_{ij} \sim \frac{c^i_{hv}\kappa^i}{N^i_h} \sum_k P_{ki}P_{jk}c^k_{vh} \tag{77}$$

In our case study $c^i_{vh} = c_{vh}$, $c^i_{hv} = c_{hv}$ and $\frac{\kappa_i}{N^i_h} = M$. It leads to $\mathcal{A}_{ij} \sim \mathcal{B}_{ij} \sim Mc_{vh}c_{hv}(\mathbf{P}^\top)^2_{ij}$. Since $\rho(\mathbf{P}^\top) = \rho((\mathbf{P}^\top)^2) = 1$, R_0 reduces to the one of the non-interacting case in our case study.

D.5 Transition between the regimes $X, X_0 \ll 1$ and $X, X_0 \gg 1$

To investigate the transition between the no interacting regime ($X, X_0 \ll 1$) and the full interacting regime ($X, X_0 \gg 1$). We write $(\mathbf{P}^\top)^2 = \mathbf{D} + \mathbf{R}$ with $\mathbf{D}$ a diagonal matrix with elements $\mathbf{D_{ii}} = (\mathbf{P}^\top)^2_{ii}$ i.e it represents the sum of weights of the reciprocal interaction between point i and any other point. Similarly $\mathbf{R}_{ij}$ represent the sum of the weight of the path of length 2 between i and j over any other points. Since $(\mathbf{P}^\top)^2$ is a positive matrix then $\rho(\mathbf{D}) \leq \rho((\mathbf{P}^\top)^2)$. Therefore replacing $(\mathbf{P}^\top)^2$ by $\mathbf{D}$ provides a lower bound for the value of R_0 in the case $X, X_0 \gg 1$. in this case, $\mathcal{A}_{ii} = \mathcal{B}_{ii} \sim (\mathbf{P}^\top)^2_{ii}c^i_{vh}c^i_{hv}\frac{\kappa^i}{N^i_h}$(off-diagonal terms are zeros). Therefore, as first approximation the R_0^F is:

$$R_0^F \approx \sqrt{\frac{T_{id}T^2_{lh}T^2_{lv}}{(T_{id}+T_{lh})(T_{iit}+T_{lh})(T_{eit}+T_{lv})} \max_i((\mathbf{P}^\top)^2_{ii}c^i_{vh}c^i_{hv}\frac{\kappa^i}{N^i_h})} \tag{78}$$

By making it equal to the approximation of R_0^1 at the first order when $X, X_0 \ll 1$ in the case it leads to:

$$P^{2\top}_{ii} = \frac{1 + XX_0(P^\top_{ii})^2}{(1+X)(1+X_0)} \tag{79}$$

$$\Leftrightarrow p^*_0 = \epsilon(\frac{1 - P^{\top 2}_{ii}}{P^{\top 2}_{ii}}) - 1 \tag{80}$$

If there $p^*_0 = 0$, we have $\epsilon^* = \frac{P^{\top 2}_{ii}}{1-P^{\top 2}_{ii}}$. The previous equation gives us an approximation of the threshold between the full interacting regimes (fast mobility) and the no interacting one (slow mobility).

E Analysis of R_0 for the diffusion model

E.1 Case $\epsilon \to 0$

We remind the expression of the matrix V:

$$\mathbf{V}\Big|_{\mathcal{X}_s} = \begin{pmatrix} \left(\frac{\mathbf{I}}{T_{lh}} + \frac{\mathbf{I}}{T_{iit}} + \frac{1}{\epsilon}\boldsymbol{\mathcal{L}}^\top\right) & \mathbf{0} & \mathbf{0} & \mathbf{0} \\ -\frac{\mathbf{I}}{T_{iit}} & \left(\frac{\mathbf{I}}{T_{lh}} + \frac{\mathbf{I}}{T_{id}} + \frac{1}{\epsilon}\boldsymbol{\mathcal{L}}^\top\right) & \mathbf{0} & \mathbf{0} \\ \mathbf{0} & \mathbf{0} & \left(\frac{\mathbf{I}}{T_{lv}} + \frac{\mathbf{I}}{T_{eit}}\right) & \mathbf{0} \\ \mathbf{0} & \mathbf{0} & -\frac{\mathbf{I}}{T_{eit}} & \frac{\mathbf{I}}{T_{lv}} \end{pmatrix} \tag{81}$$

We focus on the first two terms of the block diagonals that are of the form $\mathbf{U} = \alpha\mathbf{I} + \frac{\boldsymbol{\mathcal{L}}^\top}{\epsilon}$. In general, this matrix is not symmetric and therefore the right and left eigenvectors are different. Since $\mathbf{U}$ is a shifted matrix of $\boldsymbol{\mathcal{L}}^\top$, they have the same eigenvectors and a shifted eigenvalues: $\nu^{(n)} = \alpha + \frac{\lambda^{(n)}}{\epsilon}$ with $\nu^{(n)}$, and$\lambda^{(n)}$ the eigenvalues of $\mathbf{U}$ and $\boldsymbol{\mathcal{L}}^\top$,respectively. We can write therefore:

$$\sum_j \mathbf{U}_{ij}\varphi_j^{(n)} = \nu^{(n)}\varphi_i^{(n)} \tag{82}$$

$$\sum_j \psi_j^{(n)}\mathbf{U}_{ji} = \nu^{(n)}\psi_i^{(n)} \tag{83}$$

where $\varphi^{(n)}$ and $\psi^{(n)}$ are respectively the right and left eigenvectors of $\mathbf{U}$. We note that it can decomposed according to : $\mathbf{U}_{ij} = \sum_n \nu^{(n)}\varphi_i^{(n)}\psi_j^{(n)}$. Since $\alpha \neq 0$, the eigenvalues of $\mathbf{U}$ are all different from zero therefore $\mathbf{U}$ is invertible:

$$(\mathbf{U^{-1}})_{ij} = \sum_n \frac{1}{\nu^{(n)}}\varphi_i^{(n)}\psi_j^{(n)} \tag{84}$$

$$= \sum_n \frac{\epsilon}{\epsilon\alpha + \lambda^{(n)}}\varphi_i^{(n)}\psi_j^{(n)} \tag{85}$$

$$\underset{\epsilon\to 0}{=} \frac{1}{\alpha}\varphi_i^{(1)}\psi_j^{(1)} \tag{86}$$

The above result being obtained by noting that the only terms different from zeros in the sum is the one associated with the eigenvalue $\lambda^1 = 0$ from $\boldsymbol{\mathcal{L}}^\top$. Therefore we also know the associated right and left eigenvectors (the stationary states of the associated diffusion with the Laplacian):

$$\varphi^{(1)} = \frac{1}{2m}\begin{pmatrix} s_1 \\ \vdots \\ s_n \end{pmatrix} \tag{87}$$

$$\psi^{(1)} = \begin{pmatrix} 1 & \dots & 1 \end{pmatrix} \tag{88}$$

The term $2m$ ensures that the $\varphi^{(1)}$ is normalized. Therefore the if we set $\mathbf{S} = \varphi^{(1)}\psi^{(1)}$, the matrix $\mathbf{U}^{-1}$ read:

$$\mathbf{U}^{-1}(\alpha) = \frac{1}{\alpha}\mathbf{S} = \frac{1}{2m\alpha}\begin{pmatrix} \varphi^{(1)} & \dots \varphi^{(1)} \end{pmatrix} \tag{89}$$

Therefore $\mathbf{S}$ is a matrix with all identical columns. It means that its eigenvalues are all zeros but one whose value is $\lambda_s = \sum_i S_{ij} = \sum_i \frac{si}{2m} = 1$. associated with the left eigenvector $\psi^{(1)}$. Furthermore, in the stationary state for every nodes l, $N_h^l(t) = N_{tot}\mathbf{S_l} = \frac{N_{tot}s_l}{2m}$ with $N_{tot} = \sum_j N_h^j$. By noting that $\left(\frac{T_{id}+T_{lh}}{T_{id}T_{lh}}\mathbf{I} + \frac{1}{\epsilon}\boldsymbol{\mathcal{L}}^\top\right)^{-1} \underset{\epsilon\to 0}{=} \frac{T_{id}T_{lh}}{T_{id}+T_{lh}}\mathbf{S}$ and

$\left(\frac{T_{iit}+T_{lh}}{T_{iit}T_{lh}}\mathbf{I} + \frac{1}{\epsilon}\boldsymbol{\mathcal{L}}^\top\right)^{-1} \underset{\epsilon\to 0}{=} \frac{T_{iit}T_{lh}}{T_{iit}+T_{lh}}\mathbf{S}$, the matrix $\boldsymbol{\mathcal{C}}$ and $\boldsymbol{\mathcal{D}}$ read (the constants are omitted for the sake of clarity):

$$\boldsymbol{\mathcal{C}} \sim \boldsymbol{\mathcal{C}'_{vh}}\boldsymbol{\mathcal{K}_h^\top}(\boldsymbol{\mathcal{C}_{hv}} \odot \boldsymbol{\kappa}^\top)\mathbf{S}^2 \tag{90}$$

$$\boldsymbol{\mathcal{D}} \sim (\boldsymbol{\mathcal{C}_{hv}} \odot \boldsymbol{\kappa}^\top)\mathbf{S^2}\boldsymbol{\mathcal{C}'_{vh}}\boldsymbol{\mathcal{K}_h^\top} \tag{91}$$

where $\mathcal{K}_v^\top = \mathbf{I}$ in our case study. We note that $\mathbf{S}^2 = \mathbf{S}$.In terms of matrix elements it read:

$$\mathcal{C}_{ij} \sim \sum_l c^i_{vh}\kappa_l\mathcal{K}^\top_{h,il}\frac{c^l_{hv}s_l}{2mN_h^l} \sim \sum_l \frac{c^i_{vh}\kappa_l\mathcal{K}^\top_{h,il}c^l_{hv}}{N_{tot}} \tag{92}$$

$$\mathcal{D}_{ij} \sim \sum_l \frac{c^i_{hv}\kappa_i s_i c^l_{vh}\mathcal{K}^\top_{h,lj}}{2mN_h^i} \sim \sum_l \frac{c^i_{hv}\kappa_i c^l_{vh}\mathcal{K}^\top_{h,lj}}{N_{tot}} \tag{93}$$

If $\mathcal{K}_h^\top = \mathbf{I}$ i.e. in the case $X \gg 1$ and $X_0 \ll 1$:

$$\mathcal{C}_{ij} \sim \frac{c^i_{vh}\kappa_i c^i_{hv}}{N_{tot}} \tag{94}$$

$$\mathcal{D}_{ij} \sim \frac{c^i_{hv}\kappa_i c^j_{vh}}{N_{tot}} \tag{95}$$

Since $\boldsymbol{\mathcal{C}}$ has the same columns, similarly to $\mathbf{S}$, its only non-negative eigenvalue is given by $\lambda(\boldsymbol{\mathcal{C}}) \sim \sum_i \mathcal{C}_{ij} \sim \sum_i \frac{c^i_{vh}\kappa_i c^i_{hv}}{N_{tot}} =< c_{vh}\kappa c_{hv} >$. If there is no nodes above threshold in isolation then the previous average would not go over the threshold and therefore the mobility cannot induce an epidemic in this case. Actually in our case study, $\lambda(\boldsymbol{\mathcal{C}}) \sim Mc_{vh}c_{hv}$ which reduces to the non interacting case. Regarding $\boldsymbol{\mathcal{D}}$, we notice that is positive and irreducible matrix and therefore its radius $\rho(\boldsymbol{\mathcal{D}})$ respects the following inequality $min_i \sum_j \mathcal{D}_{ij} \leq \rho(\boldsymbol{\mathcal{D}}) \leq max_i \sum_j \mathcal{D}_{ij}$ according to the Perron-Frobenius theorem. Accordingly since the spectral radius of $\boldsymbol{\mathcal{D}}^\top$ is the same as the one of $\boldsymbol{\mathcal{D}}$ one can write

$$min_i \sum_j \mathcal{D}^\top_{ij} \leq \rho(\boldsymbol{\mathcal{D}}) \leq max_i \sum_j \mathcal{D}^\top_{ij} \tag{96}$$

$$min_i(c^i_{vh}) \sum_j \frac{c^j_{hv}\kappa_j}{N_{tot}} \leq \frac{(T_{id}+T_{lh})(T_{iit}+T_{lh})(T_{eit}+T_{lv})}{T_{id}T^2_{lh}T^2_{lv}}\rho(\boldsymbol{\mathcal{D}}) \leq max_i(c^i_{vh}) \sum_j \frac{c^j_{hv}\kappa_j}{N_{tot}} \tag{97}$$

In our case study, $c^i_{vh} = c_{vh}$, $c^i_{hv} = c_{hv}$ and $\frac{\kappa_i}{N_h^i} = M$ it leads to $\rho(\boldsymbol{\mathcal{D}}) \sim Mc_{hv}c_{vh}$ which reduces to the non interacting case.

If $\mathcal{K}_h^\top = \mathbf{P}^\top$ i.e. in the case $X \gg 1$ and $X_0 \gg 1$, $\lambda(\mathcal{C}) \sim \sum_i \mathcal{C}_{ij} \sim \sum_{l,i} \frac{c^i_{vh}\kappa_l\mathbf{P}^\top_{il}c^l_{hv}}{N_{tot}}$. In our case study and using $\sum_{l,i} N_h^l\mathbf{P}^\top_{il} = \sum_l N_h^l \sum_i \mathbf{P}_{li} = \sum_l N_h^l = N_{tot}$ it leads to the $\lambda(\boldsymbol{\mathcal{C}}) \sim Mc_{hv}c_{vh}$ which reduces to the non-interacting case. The elements of $\boldsymbol{\mathcal{D}}$ become $\mathcal{D}_{ij} \sim \sum_l \frac{c^i_{hv}\kappa_i c^l_{vh}\mathbf{P}^\top_{h,lj}}{N_{tot}}$ which under the condition of the case study become $\mathcal{D}_{ij} \sim Mc_{vh}c_{hv}\sum_l \frac{N_h^i\mathbf{P}_{jl}}{N_{tot}} \sim Mc_{vh}c_{hv}\frac{N_h^i}{N_{tot}}$. Since it becomes matrix where all columns are equal the spectral radius of $\boldsymbol{\mathcal{D}}$ read $\rho(\boldsymbol{\mathcal{D}}) \sim \sum_i \mathcal{D}_{ij} \sim Mc_{vh}c_{hv}$ which reduces to the non-interacting case.

References

1. Soneira RM, Peebles PJE. A computer model universe: simulation of the nature of the galaxy distribution in the lick catalog. The Astronomical Journal. 1978;83(7):845.
2. González-Val R. Lognormal city size distribution and distance. Economics Letters. 2019;181:7–10. doi:10.1016/j.econlet.2019.04.026.
3. Arshad S, Hu S, Ashraf BN. Zipf's law and city size distribution: A survey of the literature and future research agenda. Physica A: Statistical Mechanics and its Applications. 2018;492:75–92. doi:10.1016/j.physa.2017.10.005.
4. Simini F, González MC, Maritan A, Barabási AL. A universal model for mobility and migration patterns. Nature. 2012;484(7392):96–100. doi:10.1038/nature10856.
5. Otero M, Schweigmann N, Solari HG. A Stochastic Spatial Dynamical Model for Aedes Aegypti. Bulletin of Mathematical Biology. 2008;70(5):1297–1325. doi:10.1007/s11538-008-9300-y.
6. Manore CA, Hickmann KS, Xu S, Wearing HJ, Hyman JM. Comparing dengue and chikungunya emergence and endemic transmission in *A. aegypti* and *A. albopictus*. Journal of Theoretical Biology. 2014;356:174–191. doi:10.1016/j.jtbi.2014.04.033.
7. Ferguson NM, Cucunubá ZM, Dorigatti I, Nedjati-Gilani GL, Donnelly CA, Basáñez MG, et al. Countering the Zika epidemic in Latin America. Science. 2016;353(6297):353–354. doi:10.1126/science.aag0219.
8. Kucharski AJ, Funk S, Eggo RM, Mallet HP, Edmunds WJ, Nilles EJ. Transmission Dynamics of Zika Virus in Island Populations: A Modelling Analysis of the 2013–14 French Polynesia Outbreak. PLOS Neglected Tropical Diseases. 2016;10(5):e0004726. doi:10.1371/journal.pntd.0004726.
9. Zardini A, Menegale F, Gobbi A, Manica M, Guzzetta G, d'Andrea V, et al. Estimating the potential risk of transmission of arboviruses in the Americas and Europe: a modelling study. The Lancet Planetary Health. 2024;8(1):e30–e40. doi:10.1016/S2542-5196(23)00252-8.
10. Marini F, Caputo B, Pombi M, Travaglio M, Montarsi F, Drago A, et al. Estimating Spatio-Temporal Dynamics of Aedes Albopictus Dispersal to Guide Control Interventions in Case of Exotic Arboviruses in Temperate Regions. Scientific Reports. 2019;9(1):10281. doi:10.1038/s41598-019-46466-4.
11. Eritja R, Palmer JRB, Roiz D, Sanpera-Calbet I, Bartumeus F. Direct Evidence of Adult Aedes albopictus Dispersal by Car. Scientific Reports. 2017;7(1):14399. doi:10.1038/s41598-017-12652-5.
12. Newton EAC, Reiter P. A Model of the Transmission of Dengue Fever with an Evaluation of the Impact of Ultra-Low Volume (ULV) Insecticide Applications on Dengue Epidemics. The American Journal of Tropical Medicine and Hygiene. 1992;47(6):709–720. doi:10.4269/ajtmh.1992.47.709.
13. Kao YH, Eisenberg MC. Practical unidentifiability of a simple vector-borne disease model: Implications for parameter estimation and intervention assessment. Epidemics. 2018;25:89–100. doi:10.1016/j.epidem.2018.05.010.
14. Johansson MA, Powers AM, Pesik N, Cohen NJ, Staples JE. Nowcasting the Spread of Chikungunya Virus in the Americas. PLOS ONE. 2014;9(8):e104915. doi:10.1371/journal.pone.0104915.
15. Poletti P, Messeri G, Ajelli M, Vallorani R, Rizzo C, Merler S. Transmission Potential of Chikungunya Virus and Control Measures: The Case of Italy. PLOS ONE. 2011;6(5):1–12. doi:10.1371/journal.pone.0018860.
16. van den Driessche P, Watmough J. Reproduction numbers and sub-threshold endemic equilibria for compartmental models of disease transmission. Mathematical Biosciences. 2002;180(1):29–48. doi:10.1016/S0025-5564(02)00108-6.
17. Bernstein DS. Matrix Mathematics. 2nd ed. Princeton University Press; 2009.

18. Taboga M. Matrix Algebra;. Available from: https://www.statlect.com/matrix-algebra/.

19. Tennant W, Recker M. Robustness of the reproductive number estimates in vector-borne disease systems. PLoS Neglected Tropical Diseases. 2018;12(12):e0006999. doi:10.1371/journal.pntd.0006999.

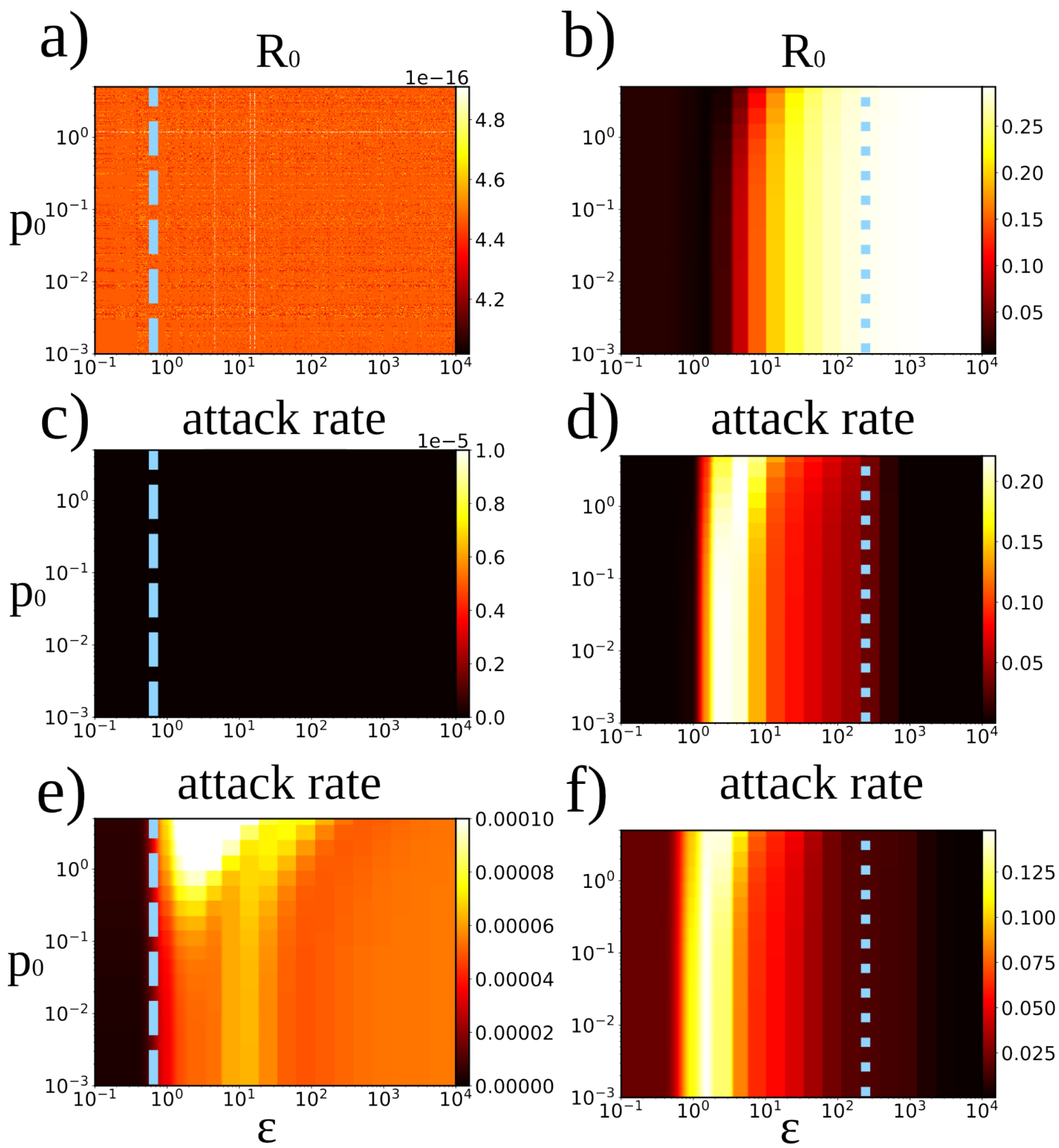

Fig A. Standard deviation of the synthetic network ensemble in the (ϵ, p_0) space. Phase diagram of the basic reproduction number R_0 for the force of infection model **a)** and the diffusion model **b)**. Attack rate without infection reservoir for the force of infection model **c)** and the diffusion model **d)**. Attack rate with infection reservoir for the force of infection model **e)** and the diffusion model **f)**. ϵ is the variable associated with human mobility and p_0 is the one associated with human-mediated vector mobility. The blue dashed line is the estimated threshold between slow and fast mobility interactions for the FoI model. The blue dotted line corresponds to $\epsilon = \frac{t_{max}}{3} = 243$, the limit after which the host mobility diffusion does not reach equilibrium within realistic time within 2 years ($t_{max} = 730$). Individual $R_0 = 0.98$ for each node.The basic reproduction number for the infection reservoir is set to $R_0^W = 2$. Results are averaged over 100 synthetic networks.

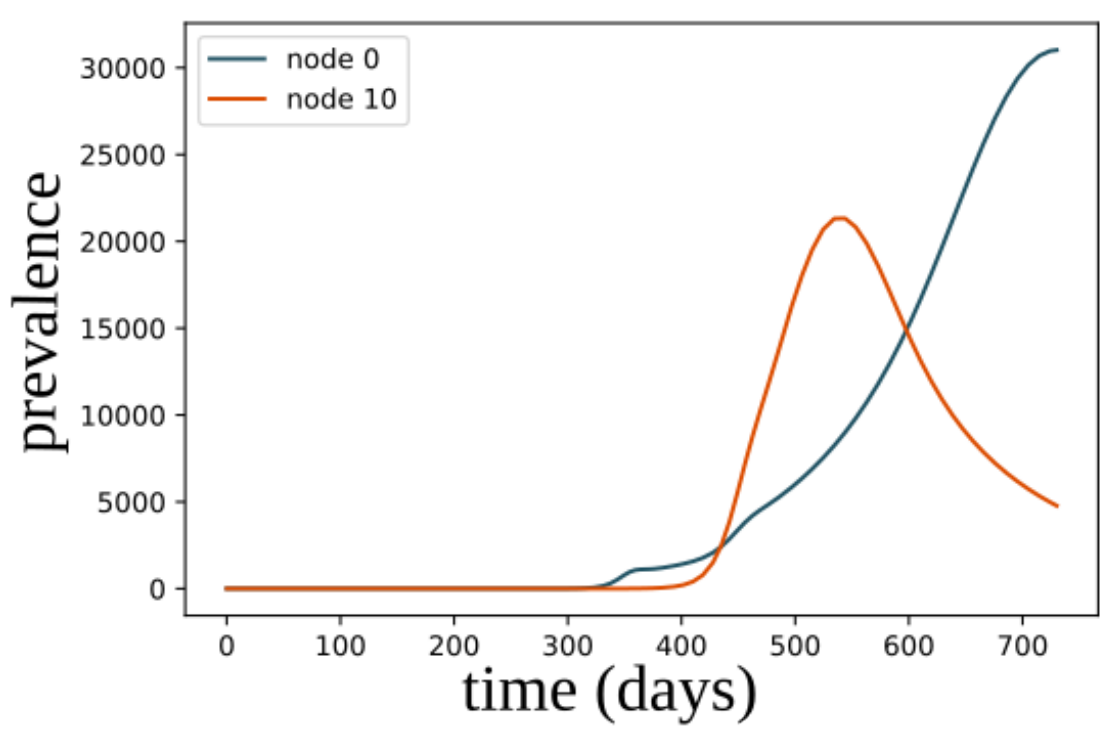

Fig B. Unfinished dynamics in realistic time for the Italian regions (NUTS2). Prevalence for hosts in function of time with $\epsilon = 100$ and $p_0 = 0.005$ obtained by the diffusion model. Individual $R_0 = 0.98$ for each node.

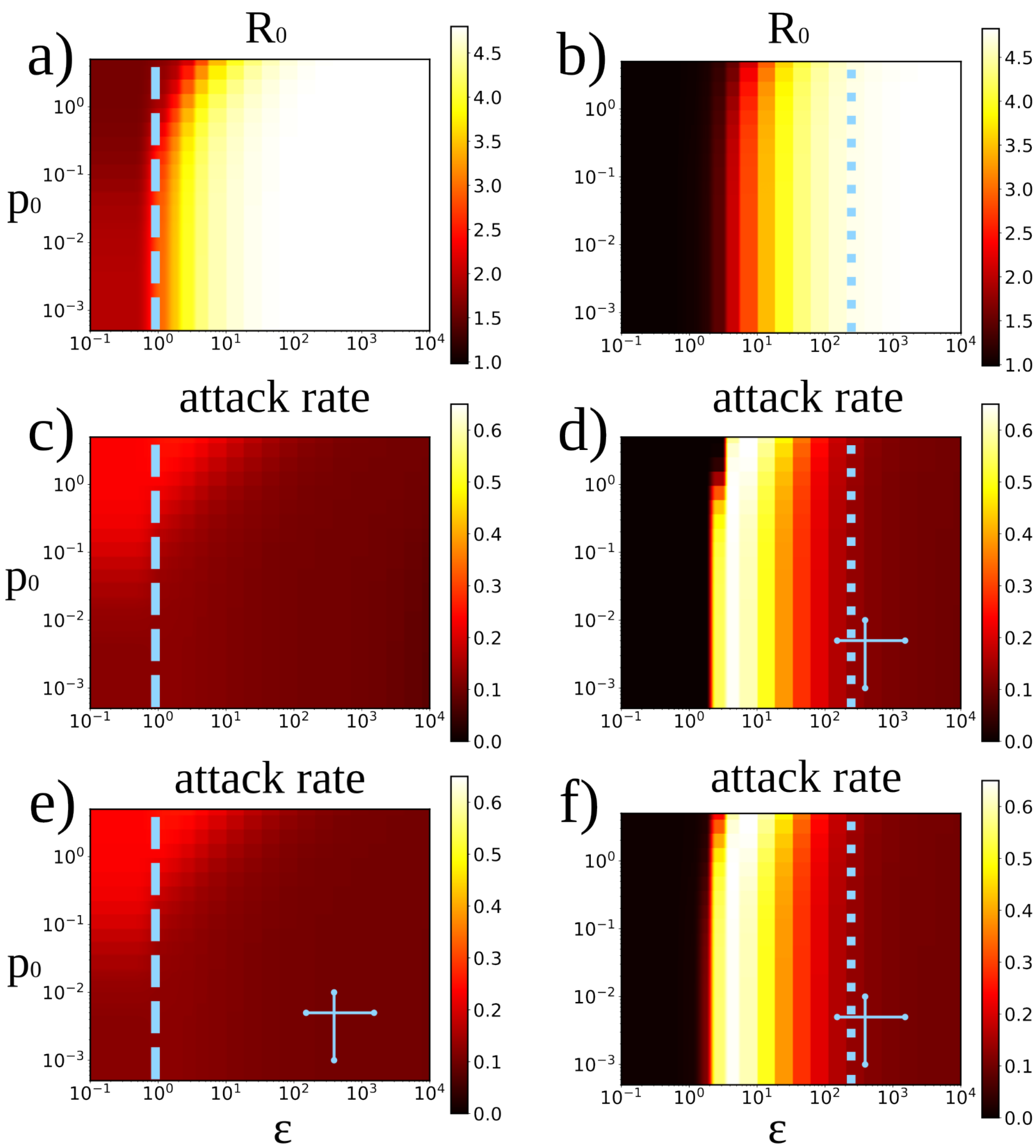

Fig C. Phase diagrams in the (ϵ, p_0) space for Italian regions (NUTS3) where host population is at equilibrium. R_0 for the force of infection model **a)** and the diffusion model **b)**. Attack rate without infection reservoir for the force of infection model **c)** and for the diffusion model **d)**. Attack rate with infection reservoir for the force of infection model **e)** and for the diffusion model **f)**. ϵ is the variable associated with human mobility and p_0 is the one associated with human-mediated vector mobility. Horizontal and vertical plain lines are the estimation of the uncertainty range for empirical ϵ and p_0. The blue dashed line is the estimated threshold between slow and fast mobility. The blue dotted line corresponds to $\epsilon = \frac{t_{max}}{3} = 243$, the limit after which the host mobility diffusion does not reach equilibrium. The host population of the FoI and diffusion model are constant equal to its equilibrium value for the diffusion model. $R_0^W = 2$ for the infection reservoir.

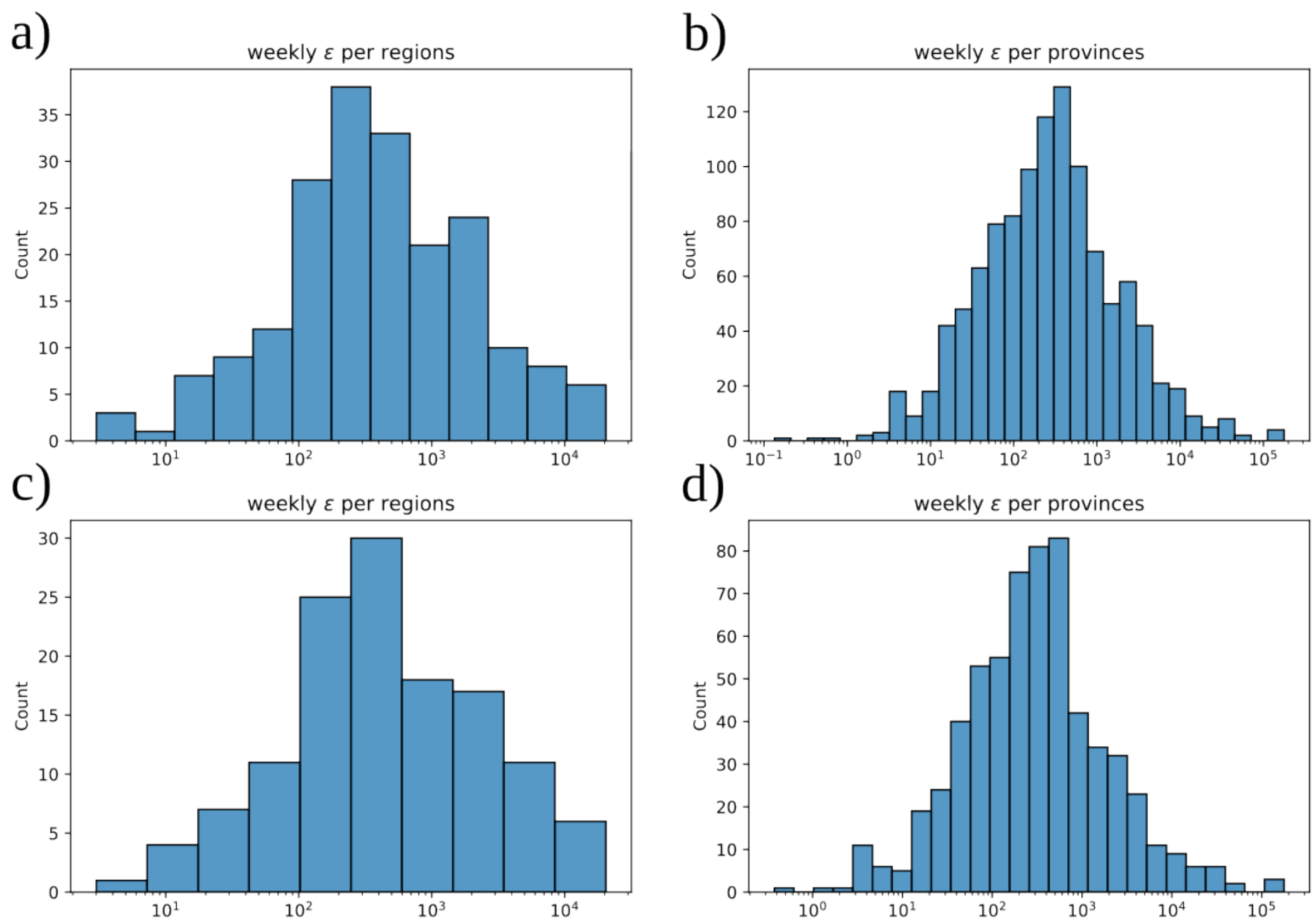

Fig D. Distribution of weekly ϵ per nodes from 16th June 2020 to 7th September 2020. Absolute values of all the ϵ obtained for **a)** Italian regions and **b)** provinces. Positive values of ϵ obtained for **c)** Italian regions and **d)** provinces.

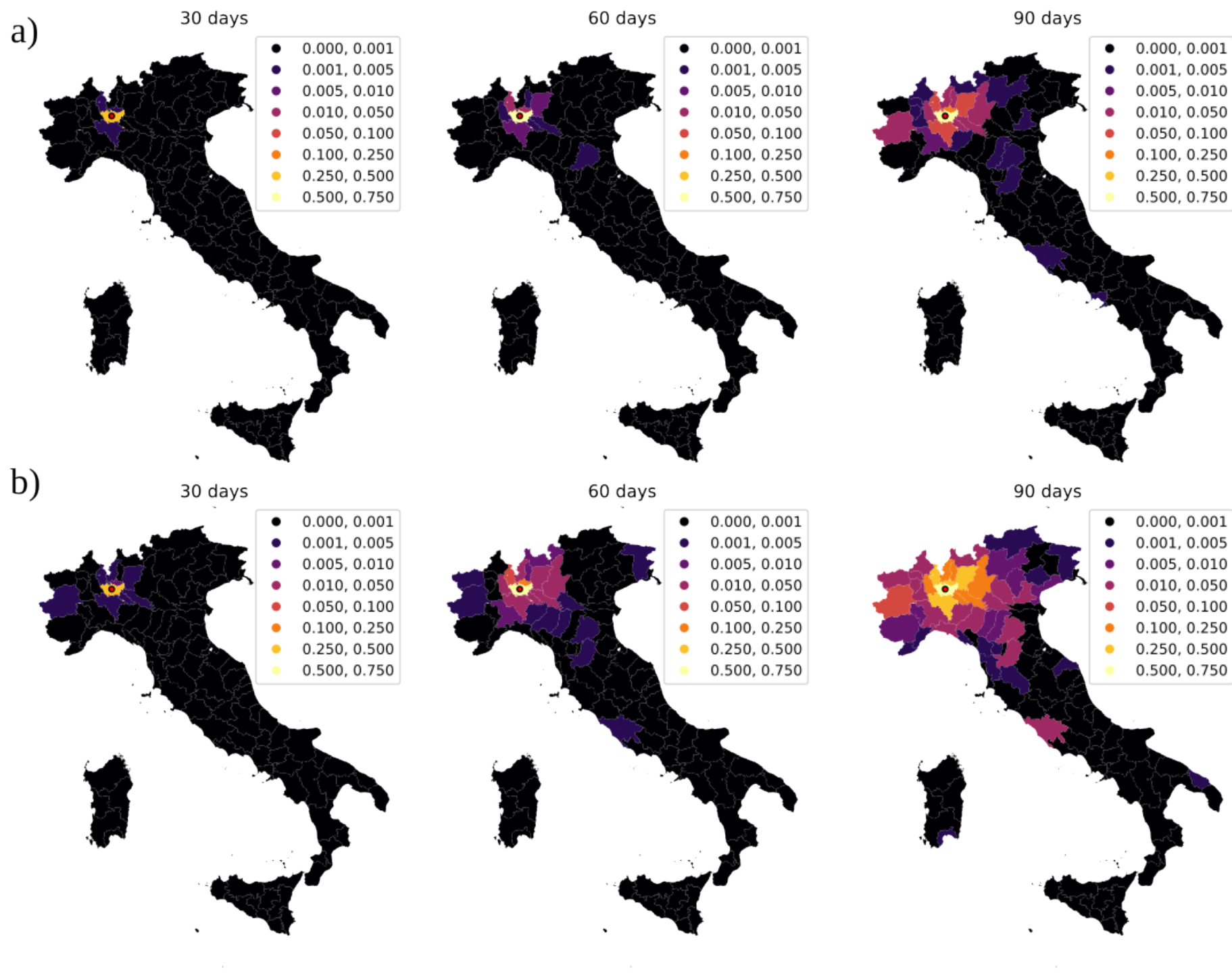

Fig E. Local importation risk for Italian provinces (NUTS3) in function of time knowing that the infection is seeded in Milan province. Probability for a province to be infected 30, 60 and 90 days after the arrival of an infected individual in Milan for the **a)** FoI model and the **b)** diffusion model. $R_0 = 2$ for each province. Fixed airline injection rate in Milan is 0.01 people per week. Province is considered infected if at least 2 individuals are present. The red points indicate where the infection is seeded. Probabilities are computed over 1000 simulations. Basemap administrative boundaries are realized by ISTAT data, via the Openpolis geojson-italy repository under the Creative Commons Attribution 4.0 (CC BY 4.0) license.

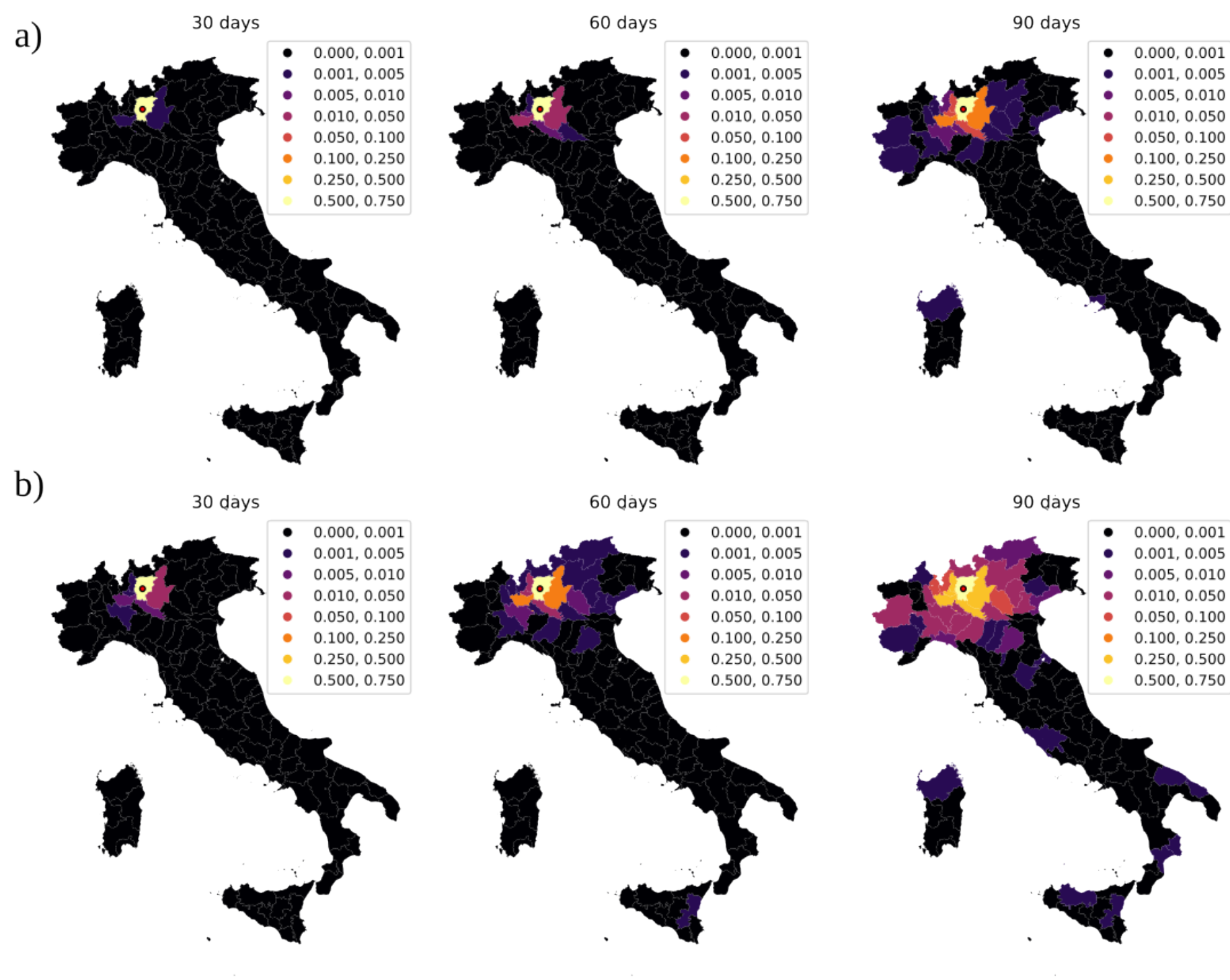

Fig F. Local importation risk for Italian provinces (NUTS3) in function of time knowing that the infection is seeded in Bergamo province. Probability for a province to be infected 30, 60 and 90 days after the arrival of an infected individual in Bergamo for the **a)** FoI model and the **b)** diffusion model. $R_0 = 2$ for each province. Fixed airline injection rate in Bergamo is 0.01 people per week. Province is considered infected if at least 2 individuals are present. The red points indicate where the infection is seeded. Probability are computed over 1000 simulations. Basemap administrative boundaries are realized by ISTAT data, via the Openpolis geojson-italy repository under the Creative Commons Attribution 4.0 (CC BY 4.0) license.

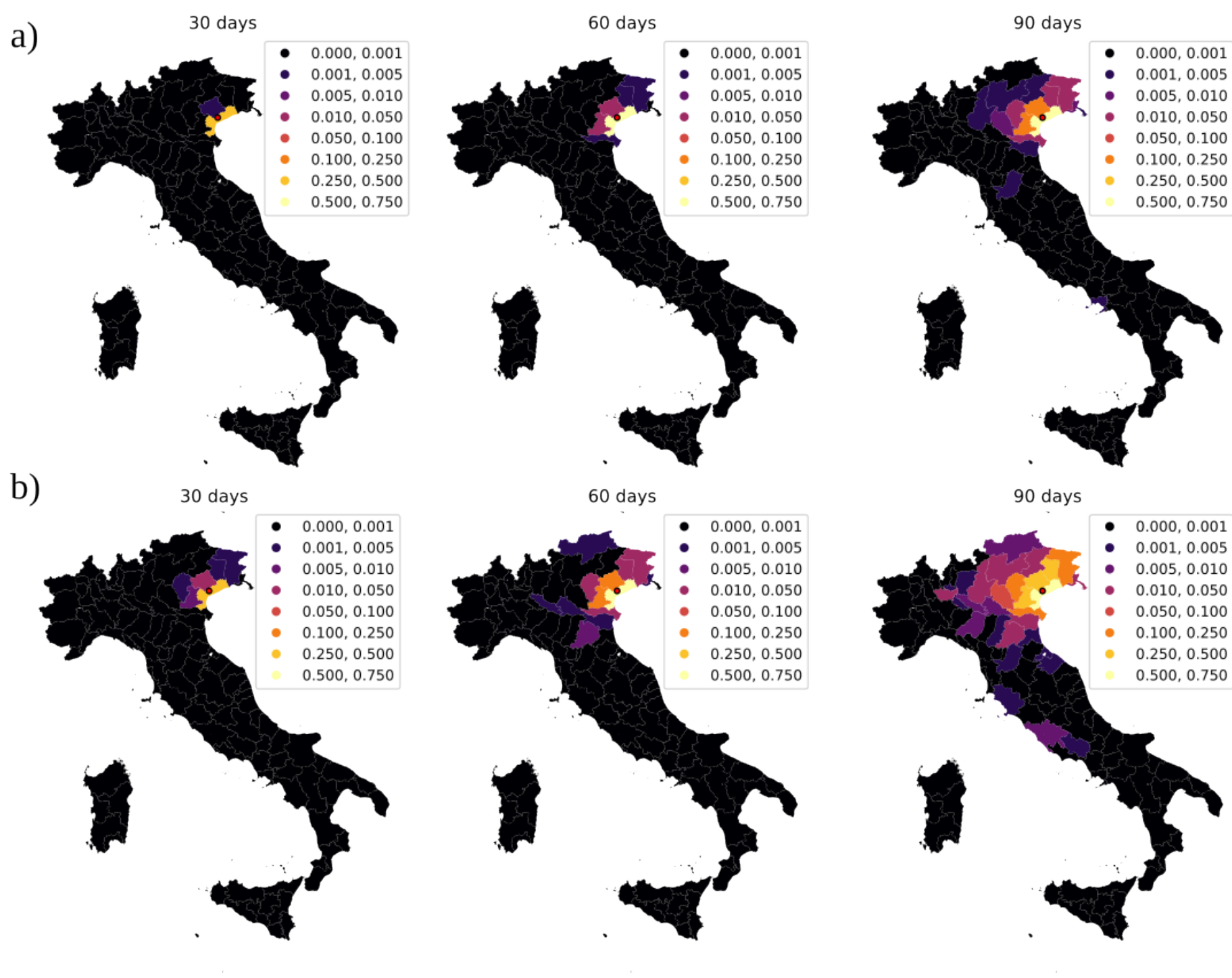

Fig G. Local importation risk for Italian provinces (NUTS3) in function of time knowing that the infection is seeded in Venice province. Probability for a province to be infected 30, 60 and 90 days after the arrival of an infected individual in Venice for the **a)** FoI model and the **b)** diffusion model. $R_0 = 2$ for each province. Fixed airline injection rate in Venice is 0.01 people per week. Province is considered infected if at least 2 individuals are present.The red points indicate where the infection is seeded. Probabilities are computed over 1000 simulations. Basemap administrative boundaries are realized by ISTAT data, via the Openpolis geojson-italy repository under the Creative Commons Attribution 4.0 (CC BY 4.0) license.

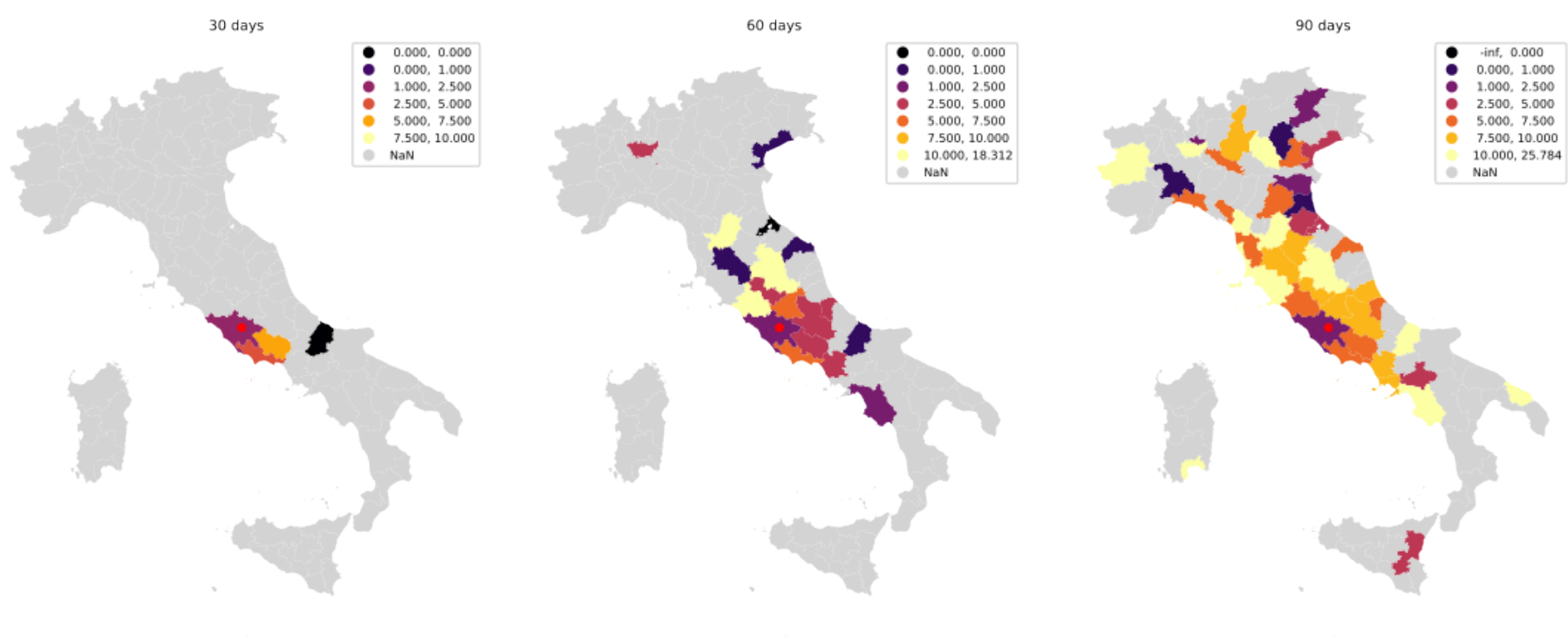

Fig H. Odds ratio of being infected in the diffusion model over the FOI model for Italian provinces (NUTS3) in function of time knowing that the infection is seeded according to the polycentric scenario. Odds ratio for a province to be infected ar represented for 30,60 and 90 days after the arrival of an infected individual. Fixed airline injection rate is 0.01 people per week distributed across the international airports according to their weight. Province is considered infected if at least 2 individuals are present. Odds are computed over 1000 simulations. Basemap administrative boundaries are realized by ISTAT data, via the Openpolis geojson-italy repository under the Creative Commons Attribution 4.0 (CC BY 4.0) license.
