# Supplementary material for: How host mobility formulations shape estimates of pathogen dispersal and epidemic risk in non endemic regions
Source: PLoS Comput Biol. 2026 Aug 24;22(8):e1014646. doi: 10.1371/journal.pcbi.1014646 (PMC13533434; doi:10.1371/journal.pcbi.1014646)
Supplement: Supplementary file 2 [file pcbi.1014646.s003.pdf]

Dear Editor,

Thank you for editing our manuscript. We have greatly appreciated the comments of the reviewers that provided invaluable insights which significantly improved the manuscript in terms of framing, clarity and scope of the results.

Accordingly, we have revised the manuscript to emphasize its focus on the main question of our study: how the two mobility formulations compare in shaping estimates of epidemic risk, in non-endemic regions in particular. To reflect this focus we proposed the following revised title: "*How host mobility formulations shape estimates of pathogen dispersal and epidemic risk in non endemic regions*".

We significantly revised the manuscript with the following enhancements:

- The Abstract was restructured to convey more precisely the main results of the study, aligned with the research question. Author Summary was revised accordingly.
- In Introduction, we revised the literature on force-of-infection (FoI) models by including existing studies developing the concept of "residence-time matrix". Furthermore, we emphasize the comparative qualitative characteristics of diffusion and FoI to describe particular mobility patterns.
- In Methods, we normalized and corrected the terminology that describes the variables of the models and we justify our choice to present part of the models in the main text.
- In Results, we enhanced the results presented in Fig 2 using an ensemble of 100 synthetic networks instead of 10 in the previous version of the manuscript. Standard deviations associated with this ensemble are presented in Fig.S1.
- In Results, we merge the results obtained with the attack rate and the basic reproduction number in one section.
- In Results, we added the section "Comparison between mobility formulations with fixed host population", where we compare the diffusion and force models where the host population is kept fixed in both models. In this section we quantify and compare estimates from the two formulations and provide a mechanistic explanation of their divergence (where they diverge).
- In Results, we merge the estimation of the value of the mobility parameters in the case of Italy with the results on the timing of epidemic spread. We revised this section to emphasize the temporal dynamics of epidemic spread following importation under realistic conditions, while excluding the PageRank-based results, thereby removing narrative elements aimed at formally estimating risk in Italy. To insist on this aspect we rename the section: "Importation risk of vector-borne diseases in real-world conditions "
- The Discussion was thoroughly reframed to integrate the new results of the section "Comparison between mobility formulations with fixed host population", to emphasize results on Italy as illustrative of realistic conditions, to make explicit where each formulation would be a priori more relevant (e.g. diffusion with migrations) and to provide streamlined conclusions and suggestions regarding the main question of the study.

The code for the analysis will be made available upon publication. Detailed point-by-point responses follow.

Reviewer #1

The study compares the effect of various types of human mobility and vectors on vector-borne diseases. To achieve this, it uses two metapopulation models that account for vector mobility and performs computational simulations to contrast the differences between a "Lagrangian" force of infection model and a diffusion model.

The approach and comparison are interesting and relevant; however, the work should also be framed in relation to other studies on control and analysis of similar metapopulations, such as: Controlling Dispersal Dynamics of Aedes aegypti. Mathematical Population Studies. 2006; 13(4):215–236; Perspectives on the role of mobility, behavior, and time scales in the spread of diseases. Proceedings of the National Academy of Sciences. 2016; 113(51):14582–14588; Vector-borne diseases models with residence times—a Lagrangian perspective. Mathematical Biosciences. 2016; 281:128–138; Vector-borne disease risk indexes in spatially structured populations. PLOS Neglected Tropical Diseases. 2018; The risk matrix of vector-borne diseases in metapopulation networks and its relation with local and global R0. Communications in Nonlinear Science and Numerical Simulation (2018); Final size of the epidemic for metapopulation vector-borne diseases. Journal of Mathematical Analysis and Applications. 2023.

We appreciate the positive feedback on the relevance of our approach.

We thank the reviewer for having put us in contact with the literature related to the residence time matrix that is indeed relevant to provide a fair state-of-the-art on “Lagrangian” force of infection model in the context of vector-borne disease. We added in Introduction the following lines: “A notable example is provided by vector-borne disease models incorporating residence times, where host mobility is represented by the fraction of time individuals from a given patch spend in others [30, 31].This framework captures the resulting effective population in each patch and has been applied to derive epidemiological metrics such as risk indices and epidemic final sizes [32, 33].” The first mentioned reference is cited when introducing the metapopulation framework.

The models are not properly explained. For example, on line 82, it is mentioned that the elements of matrix P_ij represent the fraction of individuals leaving node i and traveling to j. However, in the next line, it is confused with the flow of individuals. Flow is something different and has different units than fractions of individuals. Therefore, I consider the assumption P_ii=0 to be incorrect, since it represents the fraction of individuals, and there are always individuals who remain in their same node. That is, P_ii=0 is not the same as no flow, since P_ii does not represent flow in the model.

We are grateful to the reviewer for pointing out this confusion between flow and fraction of individuals that was present in the manuscript . Indeed, P_ij is the fraction of moving individuals departing from i to travel to j. In this regard, P_ii=0 is correct as P accounts only for the moving fraction of individuals. In the section “Metapopulation models”, we corrected and systematised the terminology for the parameters. In particular, in the text and in Table 1 we define P_ij as “the conditional probability that an individual departing from node i travels to node j”.

The models are very relevant for evaluating the conclusions, so both models should be explained term by term in more detail and in their complete form. Then, justify why it would only be necessary to focus on two equations.

We thank the reviewer for having raised this point that can be confusing to the reader. The models are based on a classical SEIR-SEI including birth-death processes from Newton and Ritter (1992). In comparison, the novelty and main focus of our models lie into the definition of the infection kernels, the infection reservoir and the diffusion term. These terms are only present in the transition from susceptible to infectious states for both host and vector population. Therefore, we show only the variation of the susceptible states for the sake of clarity and to put the emphasis on the main contribution of this article, leaving the interested reader to the Supplementary material for the complete model. To avoid further confusion, we added the following sentence is the text(Line 112-116): "Since the mechanistic differences between the two modeling hypotheses are primarily reflected in the infection rates of susceptible entities, we focus our description on the equations for susceptible hosts (S_h^i) and susceptible vectors (S_v^i); the complete SEIR-SEI systems are detailed in Text S1):"

The estimates and results are based on averages of simulations. However, 10 simulations (Fig. 2) are extremely few to find a significant result in this type of model with multiple sources of randomness. It is possible that more simulations are needed, and confidence intervals should be calculated.

We thank the reviewer for pointing out this statistical weakness in our results on the synthetic data. We extended our results to include the average of 100 simulations (see Fig.2). The results stay qualitatively unchanged. In Fig.S1, we show the standard deviation of the 100 simulations ensemble on the phase diagram. In line 354-356, we added: "The standard deviation of the attack rate for the synthetic networks ensemble is maximal at the transition since each network undergoes it for slightly different parameters(see Fig. S1)."

Reviewer #2: The objective of this manuscript is to compare alternative models of human and vector mobility on the occurrence of vector-borne disease (VBD) outbreaks in non-endemic areas. Specifically, it compares the use of a model that represents the impact of mobility via the Force of Infection (FoI) function, in which individual hosts do not explicitly move between locations, with one that represents the impact of mobility via explicit relocation of individual hosts between locations.

I very much appreciate the effort this study to better understand how the choices made in the design of a model impact the interpretation of its resulting behaviour. The work appears to be methodologically sound. However, I have some concerns about the manuscript in its current form.

We thank the reviewer for acknowledging the relevance of the question and the soundness of our approach.

Major concerns:

1. My primary concern with this manuscript is that the model used feels more complex than is necessary to achieve this comparison. While the (applied) question of estimating importation risk in Italy is undoubtedly important, I'm not convinced that it adds much insight to the (methodological) question that motivates the paper. It would perhaps be a different situation if there was empirical data on the location of importations/outbreaks that could be used to validate the choice of one model over another, but I imagine that such data is, for now at least, fortunately scarce.
A related concern is the choice of the diffusion model as the baseline for comparison with the FoI model. As acknowledged in the introduction of your manuscript, it implies unrealistic assumptions about people's behaviour and much more realistic models based on diverse empirical data sources have subsequently been put forward.
This comparison could be warranted if there was some specific insight derived from the difference between these two models. However, it appears that the mechanistic explanation for the observed differences in behaviour is the fact that the diffusion model alters the population distribution, and hence the vector-host ratio, across the metapopulations. Aside from a few specific instances (seasonal migration, or long-term demographic change) this seems like an example of the unrealistic behaviour implied by the diffusion model.

We highly appreciate the reviewer's comment which, we believe, greatly improves the manuscript. We agree that the framing of the manuscript was confusing as it could be interpreted as an attempt to estimate the vector-borne risk in Italy. Rather, we think about the analysis on Italy as a continuation of the comparison between the two mobility formulations, in real-world conditions. While our theoretical analysis is interested in the comparative assessment in the epidemic emergence risk, our application to Italy is interested in the risk of epidemic spread and timing in real-world conditions. In this analysis, our objective is to show that, far from being only a theoretical analysis whose conclusions are not relevant in realistic settings, differences (but also similarities) persist between the two formulations in real-world conditions. We thoroughly reframed the Abstract, Introduction, the section "Importation risk of vector-borne diseases in real-world conditions " and the Discussion to make clearer the role of the analysis on Italy in the comparison of the two mobility formulations.

The choice of the comparison between the force of infection model and the diffusion one arises from the fact that both are used by a broad community interested in the interplay between mobility and vector-borne disease dynamics in particular. Both models present the same number of parameters, are both parsimonious in representing the mobility interaction which allows some analytical derivation to be performed. With the introduction of Italy and the related estimation of mobility patterns, we show that both models can be also used in data-driven studies.

We acknowledge that the effective change of the host population in the diffusion model narrows the applicability of our conclusions to specific instances where important movement of population can play a role in the epidemic outcome. That is why, following the reviewer's suggestion, we performed another series of analysis where the host population is fixed at the diffusion equilibrium in both models. Results on the basic reproduction number and attack rate show that both models still exhibit critical differences in their epidemic outcome, in particular at epsilon<<1 and epsilon of the order of the unit (see Fig5 and FigS3). We interpret this difference by the ability for the diffusion model to allow the effective mobility of infected hosts that maintain growth in high-transmission areas, and to

allow the effective mobility of susceptible hosts that continuously move into nodes where transmission potential is high, sustaining the epidemic. On the contrary, the force of infection model is not accounting for the physical movement of susceptible hosts by definition. We believe that this new analysis extends the scope of our conclusions on the mobility-induced difference between the two models.

We introduced the section "Comparison between mobility formulations with fixed host population" to account for these new results and we adapted the Discussion accordingly.

2. The discussion of PageRank as an algorithm to assess relative importation risk feels tangential to the primary question, particularly given that it displays similar results under both mobility models.

We agree with the reviewer that the discussion on the PageRank algorithm is too tangential to the main question of the article. Therefore, we decided to withdraw any mention of these results in the Results and Discussion part. Following the reviewer's comment, we believe that it recenters the work towards its main question.

3. Overall, while I appreciate the effort to improve our understanding of key modelling decisions, I feel that the manuscript currently seeks to do two distinct things: (1) investigate the impact of a key modelling decision and (2) evaluate the risk of VBD outbreaks in a realistic setting. I feel that the manuscript would be stronger if it focused on one or the other of these.

We agree with the reviewer that the framing of the results regarding the risk of VBD in Italy was confusing and not perfectly aligned with the main question of the manuscript. We addressed their concerns in point 1.

Specific comments:

Check for consistency of terminology: "carriers", "vectors" (from abstract); capitalisation of "C/chikungunya", "D/dengue", "I/italian"; italicisation of "Aedes albopictus" (and noting that this is a mosquito species)

We made the mentioned terminology consistent across the text and Supporting Information. We now mention that Aedes albopictus is a mosquito species.

Title: The title is nice, but it seems to me it does not precisely match the body of the manuscript, which is focused on the impact of decisions about model structure on estimates of outbreak characteristics.

We agree with the reviewer that the title can be more aligned with the content of the manuscript. To insist on the impact of the mobility assumptions we propose the following title: " How host mobility formulations shape estimates of pathogen dispersal and epidemic risk in non endemic regions"

Abstract: The structure of the argument could be conveyed more clearly. Phrases such as "the impact of specific modeling hypotheses on human mobility remain elusive" are imprecise, as modelling hypotheses have no impact on (empirical) human mobility.

We thank the reviewer for the remark. The quoted sentence was indeed imprecise and we replaced it by the sentence: "In this context, the impact of specific modeling formulations of human mobility on the epidemic predictions remains elusive". We thoroughly revised the Abstract to convey more clearly our approach and our findings regarding our goal to compare the epidemic estimate arising from the two different human mobility formulations.

Introduction:

P2; L31: Do you also consider models that incorporate "more realistic mobility patterns" (ie, data-informed diffusion?)

In the manuscript, the mobility pattern of the diffusion model is not purely random (so as the force-of-infection model). Actually the conditional probability P_ij that an individual departing from node i travels to node j is data-driven and constrains the diffusion. In this sense we consider a heterogeneous diffusion model. In the Introduction we mention the existence of more sophisticated models that include specifically commuting patterns, memory effects or differential patterns according to the socio-economical class to provide a fair state-of-the-art to the reader.

To avoid this confusion, in Introduction, we do not introduce diffusion-like models as *random diffusion* but simply as diffusion.

P3; L41: "long-range airline travel" would seem to be a subset of "host mobility"

We agree with the reviewer and we changed "host mobility" into "host mobility within the country" to emphasize the shorter range of this mobility as compared with the airline one, which operates out of the metapopulation system.

P3; L46: given the limitations of the random diffusion assumption that are noted in the previous paragraph, what are the arguments for using it in a model?

As mentioned in the Introduction, the diffusion framework is widespread in the broad community interested in the interplay between mobility and (vector-borne) disease dynamics in particular. Its simplicity allows it to perform analytical computation and it is easily aligned, in terms of parameters, with the force of the infection model. In the diffusion model that we consider the probability to move from one node to another depends on the node which means that the diffusion is heterogeneous and not purely random. Actually, when studying Italy, we constraint this probability with data.

Importantly, the diffusion model seems adequate in modeling movements that are temporary but prolonged (e.g., much longer than the average generation time), such as long tourist stays, seasonal relocation to secondary residences, or migration flows. In such

cases, mobility can effectively relocate infectious individuals for most or all of their infectious period, making the diffusion approximation more meaningful.

To suppress any ambiguity in the diffusion model we propose, we replace any mention of "random diffusion" in the text by "diffusion". To put the emphasis on the important cases where the diffusion model is relevant, we add the following sentence in the Discussion:

"For instance, the ability of the diffusion model to modify the local vector-to-host ratio may become realistic for exploring the effects of temporary but sustained host redistribution on epidemic dynamics (e.g. seasonal labor migrations, touristic stays or migration flows), although it may overestimate short-term mobility effects. In such case, mobility can effectively relocate infectious individuals for most or all of their infectious period, making the diffusion approximation more meaningful."

Method:

P3; L74: Ensure terminology is fully defined in this section – i.e., what does a "node" refer to here?
The terminology of the section Material and Methods has been revised in order to precise and clean the terminology. In particular we added a definition of the node in the sentence: "Subpopulation of hosts and vectors interact locally within nodes that are discrete and spatially distributed units."

P6; L152: While there may have been no lockdown between June—September 2020, it seems likely that mobility patterns were affected by the COVID-19 pandemic at that time. Given that the research question is about VBDs, would it be possible to use mobility data from a period less likely to be affected by COVID-19?

We apologize for the lack of clarity on this point.

The Facebook data that covers June-September were used for the derivation of the value of epsilon, only. In terms of "who goes where" i.e. the conditional probability from people departing from one point to another point, the mobility matrices for the Italian systems were derived based on pre-lockdown mobility data collected on 18th January 2020 as described in section "Construction of the italian mobility networks". Therefore, this aspect of mobility is not affected by the posterior COVID-19 pandemic response. Although winter mobility patterns may differ from summer ones, we believe pre-pandemic mobility data are more appropriate than data collected during summer periods already affected by COVID-19 circulation.

To avoid confusion on this important point, we add the following sentence in the section "Estimating the time-scale separation parameter": "..;all other analyses in the Results section are based on the pre-lockdown mobility matrix derived from Cuebiq data, as described in the previous section."

P6; L143: epsilon was not previously defined as "velocity", and I'm not persuaded that is the best

term for the role it plays (time-scale separation). At least, it is surely always a relative measure whose value could change based upon either changes in host behaviour or changes in pathogen transmission characteristics.

We agree with the reviewer that the term "velocity" is inappropriate. In order to provide a name that suggests its function in the ODEs. we revised the text in order to rename it "time-scale separation parameter" (see Tab.1, section "estimating the time-scale separation parameter")

P6; L147: What is the "Data 4 Good Program"? Not previously mentioned.

We thank the reviewer for pointing out the absence of definition for the Data 4 Good Program. In the revised manuscript we added the following sentence: "The Meta Data 4 Good Program is a collaborative initiative that provides aggregated GPS-based datasets of Facebook users that opted-in for the collection of their data.

Results:

P9; Fig 2: I don't understand the "estimated threshold between slow and fast mobility interactions" – what does this threshold refer to and how was it estimated? Is it related to the epidemic threshold mentioned in the body of the paper?

We thank the reviewer for pointing out this element of our results that was confusing and poorly introduced in the text. On one hand, the fast mobility refers to the situation epsilon << 1 where host mobility is much faster than the infection process. On the other hand, the slow mobility refers to the situation epsilon >>1 where host mobility is slow relative to the disease spread. In the case of the force-of-infection model, we derive an analytical expression of the basic reproduction number in these two extremal regimes (see Text S3). In text S4, we use those analytical expressions of the basic reproduction number to derive the values of epsilon that constitutes the threshold between the two regimes (see Eq.11).

In the revised manuscript, we introduced the fast and slow mobility regime at the beginning of the section "Analysis of the basic reproduction number R0 and the attack rate" with the following sentences: "The parameter ϵ represents the time-scale separation between mobility and epidemic dynamics. When $\epsilon \ll 1$, host mobility is much faster than the infection process (fast mobility regime). Conversely, when $\epsilon \gg 1$, host mobility is slow relative to disease spread (slow mobility regime), and local epidemiological dynamics dominate over spatial displacement"

In addition, we revised the paragraph introducing the threshold value for epsilon in order to make it clearer for the reader.

P11; L285: I am concerned that the explanation for the observed differences in behaviour between the two models apparently arises from changes in the vector-to-host ratio (in the diffusion model). This suggests a fundamental restructuring of the population, beyond what one might expect to see over the timeframe of the simulation, i.e., that flows in one direction are balanced out by flows in another direction such that population distribution remains broadly constant. Potential exceptions to this include seasonal variation, or longer-term changes in population distribution. Have you

investigated whether these differences between model formulations persist if you control for changes in population distribution?

We are very grateful to the reviewer for this comment and the related suggestion. We totally agree that the presented results could quite limit the scope of the comparison between the two formulations to the particular cases mentioned by the reviewer. That is why we investigated the differences between the two formulations where the population is kept fixed in the diffusion model. We show that the differences persist even in this framework. In particular, for intermediate values of epsilon, we suggest that the difference is driven by the combined effect of the displacement of infected hosts which maintain the epidemic growth in high-risk areas and the displacement of susceptible hosts that replenishes the pool of susceptible in highly epidemic areas. We are grateful to the reviewer for this suggestion as we believe it widens the scope of our results. In the revised manuscript, we added the section "Comparison between formulations with fixed host population" and Figure 5,Figure S3 to describe these new results.
