# Supplementary material for: How host mobility formulations shape estimates of pathogen dispersal and epidemic risk in non endemic regions
Source: PLoS Comput Biol. 2026 Aug 24;22(8):e1014646. doi: 10.1371/journal.pcbi.1014646 (PMC13533434; doi:10.1371/journal.pcbi.1014646)
Supplement: Supplementary file 3 [file pcbi.1014646.s004.pdf]

Dear Editor,

Thank you for handling the revised version of our manuscript. We greatly appreciated the reviewers' comments, which helped to improve the reproducibility and clarity of the manuscript.

Accordingly, we provide links to:

- the GitHub repository for the code (https://github.com/CoMuNeLab/host_mobility_formulations)
- the Zenodo repository containing the publicly available datasets and generated data associated with the manuscript (https://doi.org/10.5281/zenodo.21355910).

For the remaining data with restricted access, the README or directly the Data Availability Statement provide the way to request them at third-party providers.

We update the Data Availability Statement: "All code written in support of this publication is available at https://github.com/CoMuNeLab/host_mobility_formulations. Publicly available raw data and generated data are available at the Zenodo repository. Access to restricted third-party data can be requested for the raw population data (https://simplemaps.com/data/world-cities) and for the time-resolved mobility data (https://ai.meta.com/ai-for-good/datasets/facebook-movement-during-crisis/)"

Detailed point-by-point responses follow.

Reviewer #1: The authors have addressed all of my comments.

We thank once again the reviewer for their participation in the improvement of the manuscript.

Reviewer #2: Thank you for your considered response to reviewer comments. Overall, I find the use of the Italy case study clearer and appreciate the additional results using a variant of the diffusion model in which populations are fixed.

We are delighted to have addressed all the comments of the reviewer and thank them again for their participation in the improvement of the manuscript, in particular by suggesting the fixed population setup.

My remaining comments relate to the clarity of the manuscript. Please do a thorough review of the text checking for grammatical errors, and consistent and precise use of terminology.

Note: all line numbers refer to the version of the manuscript with changes marked, which was the one that I focused on when reviewing.

We greatly appreciated the reviewer's effort to spot grammatical mistakes and inconsistencies in the terminology which dampen the clarity of the manuscript. In what follows, we address each comment separately.

- Check for single/plural agreement, eg: (in abstract) “…applications to disease such as…”; (line 22) “One of the widely adopted method…”; (line 362) “value of \epsilon and p_o”, etc.

We made a thorough review of the grammatical mistakes in the manuscript and corrected the ones indicated by the reviewers as well as other remaining ones.

- Check for precise references, eg: (line28) “the fraction of time individuals from a given patch spend in others.” would be clearer as “…spend in other patches.”

We agree with the reviewer and we included their correction to improve the clarity of the statement.

- The terms “hypothesis” and “representation” are used on several occasions (eg, lines 114, 376, 511, 597 for hypothesis and lines 501, 589, 616, 644 for representation) in a way which seems equivalent to “formulation” (ie, being “choice of how to represent mobility in a model”). The manuscript would be clearer if consistent terminology was used. (By contrast, the use of “hypothesize” on line 570 does genuinely refer to a hypothesis.)

We agree with the reviewer that the terms “hypothesis” and “representation” are equivalent terms for "formulation". In order to avoid a multiplicity of terms we replaced every relevant mention of “hypothesis” and “representation” by the word “formulation”.

-Check for consistent use of “frameworks”, “methods”, “approaches”, “models” – some of these seem to be referring to the same thing as “formulation, etc” in my previous comment. If I read two different words, I think they must refer to two distinct things – if they don’t this is confusing. I recognise that the English language is partly at fault here, as it has so many synonyms.

We agree with the reviewer that the multiplicity of terms to describe the same thing is confusing. We made a thorough review of the manuscript and reduced the terminology to the words “model” and “formulation”. The word “model” refers to the two metapopulation models including four routes of mobility presented in the manuscript. The word “formulation” refers more specifically to the different ways to represent the host mobility. More broadly, it also refers to the host mobility component in the presented metapopulation models.

- Check for other minor errors, eg: (line 162) “…probability that and individual departing…”; (line 300) “italian", (line 302) “…observe and absence of dynamics happen in this case”, (line 451) “…the attack rate substantially higher…”, (line 623) “…we focused on Italy we combining…”, etc.

We corrected the errors spotted by the reviewer. We conducted a thorough review of the manuscript to correct the remaining errors of this type. In particular, we systematically capitalized the word “italian” -> “Italian”.

- The extra info on Meta Data 4 Good Program is useful (is there a reference?) – I’m

assuming that this is the source of the “time-resolved mobility matrices” mentioned in the first sentence of this section (line 179) but this link could be made more clearly.

We agree with the reviewer that a more direct link can be drawn between the time-resolved mobility matrices and the source of the data. The introduction of the data in the manuscript now read: *The mobility parameter ϵ is estimated from time-resolved mobility matrices provided by the Meta Data 4 Good Program [57].* (line 156). The reference [57] refers to the direct link where the data can be requested from Meta.

I note the response that code will be made available on publication. For modelling papers, I would very much prefer to see code as part of the review process. PLOS Computational Biology's statement on code availability is that "Relevant code should be available to editors and reviewers at the time of submission."
(https://journals.plos.org/ploscompbiol/s/code-availability)

We thank the reviewer for mentioning this important aspect of the reproducibility of the results presented in the manuscript. The code is now available on GitHub (https://github.com/CoMuNeLab/host_mobility_formulations**). The related publicly available raw data and generated data have also been deposited on Zenodo (**https://doi.org/10.5281/zenodo.21355910**).**
