# Supplementary material for: How host mobility formulations shape estimates of pathogen dispersal and epidemic risk in non endemic regions
Source: PLoS Comput Biol. 2026 Aug 24;22(8):e1014646. doi: 10.1371/journal.pcbi.1014646 (PMC13533434; doi:10.1371/journal.pcbi.1014646)
Supplement: Peer review history

# Peer review: Decision Letter 0

**DOI:** 10.1371/journal.pcbi.1014646.r001

**Authors:** Yamir Moreno, Benjamin Althouse

2 Mar 2026

PCOMPBIOL-D-25-02326

Impact of host and pathogen dispersal on vector-borne disease risk in non-endemic areas

PLOS Computational Biology

Dear Dr. Presigny,

Thank you for submitting your manuscript to PLOS Computational Biology. After careful consideration, we have concluded that, in its current form, the manuscript does not fully meet the journal’s publication criteria. As you will see in the reports below, neither reviewer is able to recommend publication at this stage. Both indicate that substantial revisions would be required for the work to be considered suitable for PLOS Computational Biology. Accordingly, we invite you to submit a thoroughly revised version of the manuscript that addresses all points raised during the review process. Please note that without a comprehensive and carefully reasoned response to the reviewers’ comments, we will not be able to proceed further with the evaluation of the manuscript. If you choose not to address specific concerns, we ask that you provide a clear and detailed justification in your response letter. We look forward to receiving your revised submission should you decide to proceed.

Please submit your revised manuscript by May 02 2026 11:59PM. If you will need more time than this to complete your revisions, please reply to this message or contact the journal office at ploscompbiol@plos.org. When you're ready to submit your revision, log on to https://www.editorialmanager.com/pcompbiol/ and select the 'Submissions Needing Revision' folder to locate your manuscript file.

Please include the following items when submitting your revised manuscript:

* A letter that responds to each point raised by the editor and reviewer(s). You should upload this letter as a separate file labeled 'Response to Reviewers'. This file does not need to include responses to formatting updates and technical items listed in the 'Journal Requirements' section below.

* A marked-up copy of your manuscript that highlights changes made to the original version. You should upload this as a separate file labeled 'Revised Manuscript with Track Changes'.

* An unmarked version of your revised paper without tracked changes. You should upload this as a separate file labeled 'Manuscript'.

If you would like to make changes to your financial disclosure, competing interests statement, or data availability statement, please make these updates within the submission form at the time of resubmission. Guidelines for resubmitting your figure files are available below the reviewer comments at the end of this letter

We look forward to receiving your revised manuscript.

Kind regards,

Yamir Moreno

Academic Editor

PLOS Computational Biology

Benjamin Althouse

Section Editor

PLOS Computational Biology

**Journal Requirements:**

1) Please ensure that the CRediT author contributions listed for every co-author are completed accurately and in full.

At this stage, the following Authors/Authors require contributions: Charley Presigny, Piero Poletti, Stefano Merler, and Manlio De Domenico. Please ensure that the full contributions of each author are acknowledged in the "Add/Edit/Remove Authors" section of our submission form.

The list of CRediT author contributions may be found here: https://journals.plos.org/ploscompbiol/s/authorship#loc-author-contributions

2) We ask that a manuscript source file is provided at Revision. Please upload your manuscript file as a .doc, .docx, .rtf or .tex. If you are providing a .tex file, please upload it under the item type u2018LaTeX Source Fileu2019 and leave your .pdf version as the item type u2018Manuscriptu2019.

3) Tables should not be uploaded as individual files. Please remove these files and include the Tables in your manuscript file as editable, cell-based objects. For more information about how to format tables, see our guidelines:

https://journals.plos.org/ploscompbiol/s/tables

4) Some material included in your submission may be copyrighted. According to PLOSu2019s copyright policy, authors who use figures or other material (e.g., graphics, clipart, maps) from another author or copyright holder must demonstrate or obtain permission to publish this material under the Creative Commons Attribution 4.0 International (CC BY 4.0) License used by PLOS journals. Please closely review the details of PLOSu2019s copyright requirements here: PLOS Licenses and Copyright. If you need to request permissions from a copyright holder, you may use PLOS's Copyright Content Permission form.

Please respond directly to this email and provide any known details concerning your material's license terms and permissions required for reuse, even if you have not yet obtained copyright permissions or are unsure of your material's copyright compatibility. Once you have responded and addressed all other outstanding technical requirements, you may resubmit your manuscript within Editorial Manager.

Potential Copyright Issues:

- Figure 1: Please confirm whether you drew the images / clip-art within the figure panels by hand. If you did not draw the images, please provide (a) a link to the source of the images or icons and their license / terms of use; or (b) written permission from the copyright holder to publish the images or icons under our CC BY 4.0 license. Alternatively, you may replace the images with open source alternatives. See these open source resources you may use to replace images / clip-art:

- https://commons.wikimedia.org

- https://openclipart.org/.

- Figures 3, 4, 5, 6, S3, S4, S5, and S6: Please (a) provide a direct link to the base layer of the map (i.e., the country or region border shape) and ensure this is also included in the figure legend; and (b) provide a link to the terms of use / license information for the base layer image or shapefile. We cannot publish proprietary or copyrighted maps (e.g. Google Maps, Mapquest) and the terms of use for your map base layer must be compatible with our CC BY 4.0 license.

Note: if you created the map in a software program like R or ArcGIS, please locate and indicate the source of the basemap shapefile onto which data has been plotted.

If your map was obtained from a copyrighted source please amend the figure so that the base map used is from an openly available source. Alternatively, please provide explicit written permission from the copyright holder granting you the right to publish the material under our CC BY 4.0 license.

If you are unsure whether you can use a map or not, please do reach out and we will be able to help you. The following websites are good examples of where you can source open access or public domain maps:

* U.S. Geological Survey (USGS) - All maps are in the public domain. (http://www.usgs.gov)

* PlaniGlobe - All maps are published under a Creative Commons license so please cite u201cPlaniGlobe, http://www.planiglobe.com, CC BY 2.0u201d in the image credit after the caption. (http://www.planiglobe.com/?lang=enl)

* Natural Earth - All maps are public domain. (http://www.naturalearthdata.com/about/terms-of-use/).

**Reviewers' comments:**

Reviewer's Responses to Questions

**Comments to the Authors:**

**Please note here if the review is uploaded as an attachment.**

Reviewer #1: The study compares the effect of various types of human mobility and vectors on vector-borne diseases. To achieve this, it uses two metapopulation models that account for vector mobility and performs computational simulations to contrast the differences between a "Lagrangian" force of infection model and a diffusion model.

The approach and comparison are interesting and relevant; however, the work should also be framed in relation to other studies on control and analysis of similar metapopulations, such as: Controlling Dispersal Dynamics of Aedes aegypti. Mathematical Population Studies. 2006; 13(4):215–236; Perspectives on the role of mobility, behavior, and time scales in the spread of diseases. Proceedings of the National Academy of Sciences. 2016; 113(51):14582–14588; Vector-borne diseases models with residence times—a Lagrangian perspective. Mathematical Biosciences. 2016; 281:128–138; Vector-borne disease risk indexes in spatially structured populations. PLOS Neglected Tropical Diseases. 2018; The risk matrix of vector-borne diseases in metapopulation networks and its relation with local and global R0. Communications in Nonlinear Science and Numerical Simulation (2018); Final size of the epidemic for metapopulation vector-borne diseases. Journal of Mathematical Analysis and Applications. 2023.

The models are not properly explained. For example, on line 82, it is mentioned that the elements of matrix P_ij represent the fraction of individuals leaving node i and traveling to j. However, in the next line, it is confused with the flow of individuals. Flow is something different and has different units than fractions of individuals. Therefore, I consider the assumption P_ii=0 to be incorrect, since it represents the fraction of individuals, and there are always individuals who remain in their same node. That is, P_ii=0 is not the same as no flow, since P_ii does not represent flow in the model.

The models are very relevant for evaluating the conclusions, so both models should be explained term by term in more detail and in their complete form. Then, justify why it would only be necessary to focus on two equations.

The estimates and results are based on averages of simulations. However, 10 simulations (Fig. 2) are extremely few to find a significant result in this type of model with multiple sources of randomness. It is possible that more simulations are needed, and confidence intervals should be calculated.

Reviewer #2: The objective of this manuscript is to compare alternative models of human and vector mobility on the occurrence of vector-borne disease (VBD) outbreaks in non-endemic areas. Specifically, it compares the use of a model that represents the impact of mobility via the Force of Infection (FoI) function, in which individual hosts do not explicitly move between locations, with one that represents the impact of mobility via explicit relocation of individual hosts between locations.

I very much appreciate the effort this study to better understand how the choices made in the design of a model impact the interpretation of its resulting behaviour. The work appears to be methodologically sound. However, I have some concerns about the manuscript in its current form.

Major concerns:

1. My primary concern with this manuscript is that the model used feels more complex than is necessary to achieve this comparison. While the (applied) question of estimating importation risk in Italy is undoubtedly important, I’m not convinced that it adds much insight to the (methodological) question that motivates the paper. It would perhaps be a different situation if there was empirical data on the location of importations/outbreaks that could be used to validate the choice of one model over another, but I imagine that such data is, for now at least, fortunately scarce.

A related concern is the choice of the diffusion model as the baseline for comparison with the FoI model. As acknowledged in the introduction of your manuscript, it implies unrealistic assumptions about people’s behaviour and much more realistic models based on diverse empirical data sources have subsequently been put forward.

This comparison could be warranted if there was some specific insight derived from the difference between these two models. However, it appears that the mechanistic explanation for the observed differences in behaviour is the fact that the diffusion model alters the population distribution, and hence the vector-host ratio, across the metapopulations. Aside from a few specific instances (seasonal migration, or long-term demographic change) this seems like an example of the unrealistic behaviour implied by the diffusion model.

2. The discussion of PageRank as an algorithm to assess relative importation risk feels tangential to the primary question, particularly given that it displays similar results under both mobility models.

3. Overall, while I appreciate the effort to improve our understanding of key modelling decisions, I feel that the manuscript currently seeks to do two distinct things: (1) investigate the impact of a key modelling decision and (2) evaluate the risk of VBD outbreaks in a realistic setting. I feel that the manuscript would be stronger if it focused on one or the other of these.

Specific comments:

Check for consistency of terminology: “carriers”, “vectors” (from abstract); capitalisation of “C/chikungunya”, “D/dengue”, “I/italian”; italicisation of “Aedes albopictus” (and noting that this is a mosquito species)

Title: The title is nice, but it seems to me it does not precisely match the body of the manuscript, which is focused on the impact of decisions about model structure on estimates of outbreak characteristics.

Abstract: The structure of the argument could be conveyed more clearly. Phrases such as “the impact of specific modeling hypotheses on human mobility remain elusive” are imprecise, as modelling hypotheses have no impact on (empirical) human mobility.

Introduction:

P2; L31: Do you also consider models that incorporate “more realistic mobility patterns” (ie, data-informed diffusion?)

P3; L41: “long-range airline travel” would seem to be a subset of “host mobility”

P3; L46: given the limitations of the random diffusion assumption that are noted in the previous paragraph, what are the arguments for using it in a model?

Method:

P3; L74: Ensure terminology is fully defined in this section – i.e., what does a “node” refer to here?

P6; L152: While there may have been no lockdown between June—September 2020, it seems likely that mobility patterns were affected by the COVID-19 pandemic at that time. Given that the research question is about VBDs, would it be possible to use mobility data from a period less likely to be affected by COVID-19?

P6; L143: epsilon was not previously defined as “velocity”, and I’m not persuaded that is the best term for the role it plays (time-scale separation). At least, it is surely always a relative measure whose value could change based upon either changes in host behaviour or changes in pathogen transmission characteristics.

P6; L147: What is the “Data 4 Good Program”? Not previously mentioned.

Results:

P9; Fig 2: I don’t understand the “estimated threshold between slow and fast mobility interactions” – what does this threshold refer to and how was it estimated? Is it related to the epidemic threshold mentioned in the body of the paper?

P11; L285: I am concerned that the explanation for the observed differences in behaviour between the two models apparently arises from changes in the vector-to-host ratio (in the diffusion model). This suggests a fundamental restructuring of the population, beyond what one might expect to see over the timeframe of the simulation, i.e., that flows in one direction are balanced out by flows in another direction such that population distribution remains broadly constant. Potential exceptions to this include seasonal variation, or longer-term changes in population distribution. Have you investigated whether these differences between model formulation persist if you control for changes in population distribution?

**********

**Have the authors made all data and (if applicable) computational code underlying the findings in their manuscript fully available?**

The PLOS Data policy requires authors to make all data and code underlying the findings described in their manuscript fully available without restriction, with rare exception (please refer to the Data Availability Statement in the manuscript PDF file). The data and code should be provided as part of the manuscript or its supporting information, or deposited to a public repository. For example, in addition to summary statistics, the data points behind means, medians and variance measures should be available. If there are restrictions on publicly sharing data or code —e.g. participant privacy or use of data from a third party—those must be specified.

Reviewer #1: Yes

Reviewer #2: **No:** While full mathematical details are provided in the supplementary information, I strongly encourage the authors to make their simulation code available (eg, via github repository or similar), as it greatly aids transparency and reproducibility.

**********

PLOS authors have the option to publish the peer review history of their article (what does this mean?). If published, this will include your full peer review and any attached files.

If you choose “no”, your identity will remain anonymous but your review may still be made public.

**Do you want your identity to be public for this peer review?**  For information about this choice, including consent withdrawal, please see our Privacy Policy.

Reviewer #1: No

Reviewer #2: No

[NOTE: If reviewer comments were submitted as an attachment file, they will be attached to this email and accessible via the submission site. Please log into your account, locate the manuscript record, and check for the action link "View Attachments". If this link does not appear, there are no attachment files.]

**Figure resubmission:**

**Reproducibility:**

?>

# Author comment: Author response to Decision Letter 1

**DOI:** 10.1371/journal.pcbi.1014646.r002

13 May 2026

Attachment Submitted filename: response_to_reviewers.pdf

# Peer review: Decision Letter 1

**DOI:** 10.1371/journal.pcbi.1014646.r003

**Authors:** Yamir Moreno, Benjamin Althouse

24 Jun 2026

PCOMPBIOL-D-25-02326R1

How host mobility formulations shape estimates of pathogen dispersal and epidemic risk in non endemic regions

PLOS Computational Biology

Dear Dr. Presigny,

Thank you for submitting your manuscript to PLOS Computational Biology. After careful consideration, we feel that it is likely that we will accept your MS for publication in PLOS Computational Biology if some remaining criticisms are duly addressed. Therefore, we invite you to submit a revised version of the manuscript that addresses the points that you can find in the reviewer's report below.

Please submit your revised manuscript by Aug 24 2026 11:59PM. If you will need more time than this to complete your revisions, please reply to this message or contact the journal office at ploscompbiol@plos.org. When you're ready to submit your revision, log on to https://www.editorialmanager.com/pcompbiol/ and select the 'Submissions Needing Revision' folder to locate your manuscript file.

Please include the following items when submitting your revised manuscript:

* A letter that responds to each point raised by the editor and reviewer(s). You should upload this letter as a separate file labeled 'Response to Reviewers'. This file does not need to include responses to formatting updates and technical items listed in the 'Journal Requirements' section below.

* A marked-up copy of your manuscript that highlights changes made to the original version. You should upload this as a separate file labeled 'Revised Manuscript with Track Changes'.

* An unmarked version of your revised paper without tracked changes. You should upload this as a separate file labeled 'Manuscript'.

If you would like to make changes to your financial disclosure, competing interests statement, or data availability statement, please make these updates within the submission form at the time of resubmission. Guidelines for resubmitting your figure files are available below the reviewer comments at the end of this letter.

As the corresponding author, your ORCID iD is verified in the submission system and will appear in the published article. PLOS supports the use of ORCID, and we encourage all coauthors to register for an ORCID iD and use it as well. Please encourage your coauthors to verify their ORCID iD within the submission system before final acceptance, as unverified ORCID iDs will not appear in the published article. Only the individual author can complete the verification step; PLOS staff cannot verify ORCID iDs on behalf of authors.

We look forward to receiving your revised manuscript.

Kind regards,

Yamir Moreno

Academic Editor

PLOS Computational Biology

Benjamin Althouse

Section Editor

PLOS Computational Biology

**Additional Editor Comments (if provided):**

**Reviewers' comments:**

Reviewer's Responses to Questions

**Comments to the Authors:**

**Please note here if the review is uploaded as an attachment.**

Reviewer #1: The authors have addressed all of my comments.

Reviewer #2: Thank you for your considered response to reviewer comments. Overall, I find the use of the Italy case study clearer and appreciate the additional results using a variant of the diffusion model in which populations are fixed.

My remaining comments relate to the clarity of the manuscript. Please do a thorough review of the text checking for grammatical errors, and consistent and precise use of terminology.

Note: all line numbers refer to the version of the manuscript with changes marked, which was the one that I focused on when reviewing.

- Check for single/plural agreement, eg: (in abstract) “…applications to disease such as…”; (line 22) “One of the widely adopted method…”; (line 362) “value of \epsilon and p_o”, etc.

- Check for precise references, eg: (line28) “the fraction of time individuals from a given patch spend in others.” would be clearer as “…spend in other patches.”

- The terms “hypothesis” and “representation” are used on several occasions (eg, lines 114, 376, 511, 597 for hypothesis and lines 501, 589, 616, 644 for representation) in a way which seems equivalent to “formulation” (ie, being “choice of how to represent mobility in a model”). The manuscript would be clearer if consistent terminology was used. (By contrast, the use of “hypothesize” on line 570 does genuinely refer to a hypothesis.)

-Check for consistent use of “frameworks”, “methods”, “approaches”, “models” – some of these seem to be referring to the same thing as “formulation, etc” in my previous comment. If I read two different words, I think they must refer to two distinct things – if they don’t this is confusing. I recognise that the English language is partly at fault here, as it has so many synonyms.

- Check for other minor errors, eg: (line 162) “…probability that and individual departing…”; (line 300) “italian", (line 302) “…observe and absence of dynamics happen in this case”, (line 451) “…the attack rate substantially higher…”, (line 623) “…we focused on Italy we combining…”, etc.

- The extra info on Meta Data 4 Good Program is useful (is there a reference?) – I’m assuming that this is the source of the “time-resolved mobility matrices” mentioned in the first sentence of this section (line 179) but this link could be made more clearly.

I note the response that code will be made available on publication. For modelling papers, I would very much prefer to see code as part of the review process. PLOS Computational Biology's statement on code availability is that "Relevant code should be available to editors and reviewers at the time of submission." (https://journals.plos.org/ploscompbiol/s/code-availability)

**********

**Have the authors made all data and (if applicable) computational code underlying the findings in their manuscript fully available?**

The PLOS Data policy requires authors to make all data and code underlying the findings described in their manuscript fully available without restriction, with rare exception (please refer to the Data Availability Statement in the manuscript PDF file). The data and code should be provided as part of the manuscript or its supporting information, or deposited to a public repository. For example, in addition to summary statistics, the data points behind means, medians and variance measures should be available. If there are restrictions on publicly sharing data or code —e.g. participant privacy or use of data from a third party—those must be specified.

Reviewer #1: None

Reviewer #2: **No:** Authors state that code will be made available on publication.

**********

PLOS authors have the option to publish the peer review history of their article (what does this mean?). If published, this will include your full peer review and any attached files.

If you choose “no”, your identity will remain anonymous but your review may still be made public.

**Do you want your identity to be public for this peer review?**  For information about this choice, including consent withdrawal, please see our Privacy Policy.

Reviewer #1: No

Reviewer #2: No

[NOTE: If reviewer comments were submitted as an attachment file, they will be attached to this email and accessible via the submission site. Please log into your account, locate the manuscript record, and check for the action link "View Attachments". If this link does not appear, there are no attachment files.]

**Figure resubmission:**

**Reproducibility:**

To enhance the reproducibility of your results, we recommend that authors of applicable studies deposit laboratory protocols in protocols.io, where a protocol can be assigned its own identifier (DOI) such that it can be cited independently in the future. Additionally, PLOS ONE offers an option to publish peer-reviewed clinical study protocols. Read more information on sharing protocols at https://plos.org/protocols?utm_medium=editorial-email&utm_source=authorletters&utm_campaign=protocols

# Author comment: Author response to Decision Letter 2

**DOI:** 10.1371/journal.pcbi.1014646.r004

25 Jul 2026

Attachment Submitted filename: rebuttal_letter.pdf

# Editor report: Decision Letter 2

**DOI:** 10.1371/journal.pcbi.1014646.r005

**Authors:** Yamir Moreno, Benjamin Althouse

28 Jul 2026

Dear Dr Presigny,

We are pleased to inform you that your manuscript 'How host mobility formulations shape estimates of pathogen dispersal and epidemic risk in non endemic regions' has been provisionally accepted for publication in PLOS Computational Biology.

Before your manuscript can be formally accepted you will need to complete some formatting changes, which you will receive in a follow up email. A member of our team will be in touch with a set of requests.

Please note that your manuscript will not be scheduled for publication until you have made the required changes, so a swift response is appreciated.

IMPORTANT: The editorial review process is now complete. PLOS will only permit corrections to spelling, formatting or significant scientific errors from this point onwards. Requests for major changes, or any which affect the scientific understanding of your work, will cause delays to the publication date of your manuscript.

Should you, your institution's press office or the journal office choose to press release your paper, you will automatically be opted out of early publication. We ask that you notify us now if you or your institution is planning to press release the article. All press must be co-ordinated with PLOS.

Thank you again for supporting Open Access publishing; we are looking forward to publishing your work in PLOS Computational Biology.

Best regards,

Yamir Moreno

Academic Editor

PLOS Computational Biology

Benjamin Althouse

Section Editor

PLOS Computational Biology

***********************************************************

# Editor report: Acceptance letter

**DOI:** 10.1371/journal.pcbi.1014646.r006

**Authors:** Yamir Moreno, Benjamin Althouse

PCOMPBIOL-D-25-02326R2

How host mobility formulations shape estimates of pathogen dispersal and epidemic risk in non endemic regions

Dear Dr Presigny,

I am pleased to inform you that your manuscript has been formally accepted for publication in PLOS Computational Biology. Your manuscript is now with our production department and you will be notified of the publication date in due course.

The corresponding author will soon be receiving a typeset proof for review, to ensure errors have not been introduced during production. Please review the PDF proof of your manuscript carefully, as this is the last chance to correct any errors. Please note that major changes, or those which affect the scientific understanding of the work, will likely cause delays to the publication date of your manuscript.

Soon after your final files are uploaded, unless you have opted out, the early version of your manuscript will be published online. The date of the early version will be your article's publication date. The final article will be published to the same URL, and all versions of the paper will be accessible to readers.

For Research, Software, and Methods articles, you will receive an invoice from PLOS for your publication fee after your manuscript has reached the completed accept phase. If you receive an email requesting payment before acceptance or for any other service, this may be a phishing scheme. Learn how to identify phishing emails and protect your accounts at https://explore.plos.org/phishing.

Thank you again for supporting PLOS Computational Biology and open-access publishing. We are looking forward to publishing your work!

With kind regards,

Janani Seenivasan

PLOS Computational Biology | Carlyle House, Carlyle Road, Cambridge CB4 3DN | United Kingdom ploscompbiol@plos.org | Phone +44 (0) 1223-442824 | ploscompbiol.org | @PLOSCompBiol
